# Modeled Global Impacts of Chlorine Oxidation and Temperature Dependence on the Atmospheric Lifetime and Concentrations of Volatile Methyl Siloxanes

**DOI:** 10.1021/acs.est.5c08897

**Published:** 2025-12-17

**Authors:** Christopher E. Brunet, Saeideh Mohammadi, Behrooz Roozitalab, Nora K. Gibson, Rafael P. Fernandez, Alfonso Saiz-Lopez, Keri C. Hornbuckle, Charles O. Stanier

**Affiliations:** † Department of Civil and Environmental Engineering, 4083 University of Iowa, Iowa City, Iowa 52242, United States of America; ‡ IIHR-Hydroscience and Engineering, University of Iowa, Iowa City, Iowa 52242, United States; § Department of Chemical and Biochemical Engineering, University of Iowa, Iowa City, Iowa 52242, United States of America; ∥ 53593 Atmospheric Chemistry Observations and Modeling Laboratory, NSF National Center for Atmospheric Research, Boulder 80301, United States of America; ⊥ Institute for Interdisciplinary Science (ICB), National Research Council (CONICET), FCEN-UNCuyo, Mendoza 5501, Argentina; # Department of Atmospheric Chemistry and Climate, Institute of Physical Chemistry Blas Cabrera, 69568 CSIC, Madrid 28006, Spain

**Keywords:** volatile organic compounds, atmospheric modeling, long-range environmental transport, atmospheric chemistry, personal care products

## Abstract

Volatile methyl siloxanes (VMS) are high production volume chemicals found in a wide range of consumer items such as personal care products. VMS have attracted scrutiny due to long-range environmental transport (LRET) concerns. However, their emissions, lifetimes, and concentrations remain uncertain, in part because of limitations in previous atmospheric modeling. Herein, we describe the global modeling of siloxanes: D4 (octamethylcyclotetrasiloxane), D5 (decamethylcyclopentasiloxane), and D6 (dodecamethylcyclohexasiloxane) in the Community Earth System Model (CESM2-SLH) using an updated chemical mechanism that includes chlorine radical oxidation and temperature-dependent reaction rates. With these previously unconsidered factors, we predicted VMS lifetimes ranging between 2.7 and 6.7 days and annual average D5 near-surface concentrations as high as 4.5 ng m^–3^ in the remote Arctic and 160 ng m^–3^ in urban areas. These lifetimes and the degree of LRET were significantly lower than previously reported. OH remained the largest loss pathway, although it decreased at low temperatures relative to previous modeling. The temperature-induced lifetime increase was outweighed by the previously unconsidered chlorine oxidation channel. Model results and previous measurements agreed relatively well but exhibited negative normalized biases (−0.55 to −0.88), particularly in urban areas not well-resolved at global model resolution, and for passive sampler measurements.

## Graphical abstract

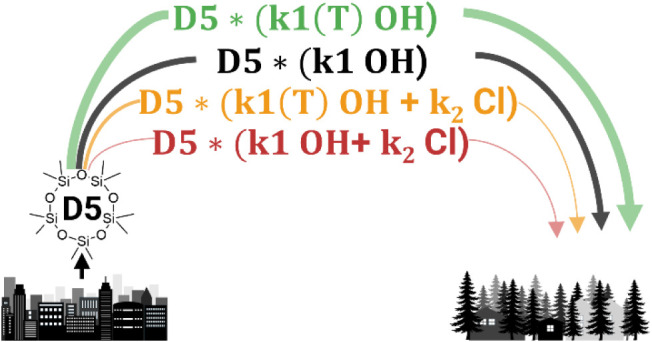

## Introduction

Over the past several decades, urban emissions of volatile organic compounds (VOC) from long-recognized sources such as vehicle emissions have significantly decreased.^1–4^ As a result, volatile chemical products (VCP) used in cleaners, lubricants, and other household products have increasingly become a major source of urban VOC emissions relative to other sources.^5–7^ Today it is estimated that VCP contribute over half of VOC emissions in some U.S. cities and that consumer products alone contribute approximately 38% of these emissions.^5^ One class of VCP-emitted VOC of particular interest is volatile methyl siloxanes (VMS). In regions where the use of VMS is not regulated, they are ubiquitously found in personal care products and are widely used in products such as waxes and lubricants. VMS are also frequently used in the synthesis of larger organosilicon chemicals such as polydimethylsiloxane.^8–14^ Over 90% of many VMS compounds put into production are eventually released into the atmosphere where they undergo reactions with hydroxyl and chlorine radicals to form less volatile, oxidized VMS (oVMS).^13,15–17^

These emissions have garnered significant attention from scientists and regulators interested in their environmental impacts. Chamber studies have repeatedly shown that VMS can form secondary organic aerosols (SOA) which has raised questions about their contributions to atmospheric particulate loading.^18–22^ Furthermore, VMS have attracted interest as VCP tracer compounds due to the fact that they do not have a natural source and are pervasively used in a known set of products.^23^ However, VMS have primarily drawn attention because of concerns that they may undergo long-range environmental transport (LRET) to remote regions such as the Arctic and Antarctic.^24–28^ In 2018, the European Chemicals Agency (ECHA) amended the REACH (Registration, Evaluation, Authorization, and Restriction of Chemicals) regulation to prohibit the use of two VMS, D4 (octamethylcyclotetrasiloxane) and D5 (decamethylcyclopentasiloxane), in wash-off personal care products after 2020 because they were considered to be very persistent and very bioaccumulative (vPvB).^29^ In 2024, a further amendment was passed that expanded these restrictions to include D6 (dodecamethylcyclohexasiloxane) and other uses such as medical devices and solvents beginning in 2026.^30^ ECHA has also drafted a proposal to add these three VMS species to Annex B of the Stockholm Convention because of the evidence for their LRET, persistence in the environment, and bioaccumulation.^31^ However, literature estimates of VMS lifetimes are highly variable and the degree to which LRET of VMS compounds occurs is still unclear. Furthermore, the proposed addition of VMS compounds to the Stockholm Convention has been questioned because of doubts that there is significant atmospheric deposition of VMS in these remote areas.^32^ In addition, focusing entirely on the environmental behavior of the unoxidized parent compound has no scientific basis and a detailed understanding of the fate and transport of both parent compounds and their degradation products (including in the aerosol phase) is needed. Because of the ongoing debate about the LRET of these compounds as well as interest in their contributions to SOA formation and their potential to serve as tracer compounds, there is a pressing need to improve our understanding and predictions of the emissions, concentrations, deposition, and lifetimes of VMS and their oxidized byproducts.

Several chemical transport models have been previously developed to assess the presence of VMS in the environment. Between 2010 and 2011, VMS concentrations were modeled using the Danish Eulerian Hemispheric Model (DEHM) and the Berkeley-Trent Global Contaminant Fate Model (BETR Global).^33,34^ The estimates produced by these models showed relatively good agreement with field measurements and provided initial insights into the range of VMS emissions and concentrations in remote, rural, and urban areas. Around the same time, a compartment model (hereafter referred to “N11”) was used to estimate the atmospheric lifetimes of two VMS, D4 and D5.^35^ More recently, D4, D5, and D6 and their oxidized byproducts were added to the Community Multiscale Air Quality (CMAQ) model (hereafter referred to as “J17”) in order to simulate their concentrations over the continental U.S.^36^ In addition to producing the first estimates of atmospheric oVMS concentrations, the VMS abundances obtained by this model showed good agreement with urban VMS measurements in the U.S.^36–38^ As a result, J17 provided evidence that previously estimated U.S. per capita emissions rates were realistic. In 2021, CMAQ was again used to model VMS, as well as several other VCP-emitted VOC, and their contributions to SOA formation over the U.S. This model was the first to extrapolate VMS-SOA yields obtained from chamber studies to an atmospheric simulation and predicted a maximum VMS-SOA concentration of 21 ng m^–3^ (only ∼0.4% of total measured SOA) for the modeled domain (Los Angeles County).^39^

While these models provided key insights into the fate and transport of VMS, they had several key limitations which must still be addressed. Most notably, the VMS oxidation mechanisms used in these models did not use temperature-dependent reaction rates and did not include reactions with chlorine radicals. Estimates of the temperature dependence of the VMS–OH oxidation rates have only been reported for the first time within the past decade.^40–43^ One of the most recent studies showed that these reaction rates may differ from those measured at room temperature by over 80% across the range of 260–370 K, with slower reaction rates at lower temperatures.^40^ As a result, VMS concentrations and lifetimes predicted in previous models may have been over, or underestimated for regions and time periods with temperatures that deviated significantly from 298 K. In particular, it is possible that the long-range transport of VMS to the Arctic and Antarctic was underestimated due to the fact that the model oxidation rates did not decrease near colder polar regions. In contrast, while it has been estimated, based on VMS-Cl oxidation rates, that chlorine oxidation only contributes approximately 5% of global VMS loss, it may be critically important in urban environments where VMS-Cl may contribute up to 30% of total VMS losses.^13^ As a result, the exclusion of chlorine oxidation in prior models may have led to significant overestimation of VMS in these regions. In addition to these mechanistic omissions, many previous models have been limited in their geographical domain, which made it impossible to compare model results to the full range of available field data or predict concentrations and deposition in regions of interest. Important physicochemical parameters such as the VMS–OH oxidation rates and o-VMS Henry’s law constants have also been refined in recent years, making the values used in previous models outdated.^13,36,44,45^

As a result, there is a need for an updated global model of VMS that incorporates the latest scientific knowledge about their atmospheric behavior, including their reactions with chlorine radicals and the temperature dependence of their reaction rates. One modeling system particularly well-suited to this task is the Community Earth System Model (CESM).^46,47^ CESM version 2 (CESM2) is a global, fully coupled model that includes land, ocean, ice, and atmosphere components. Community Atmosphere Model with Chemistry (CAM-chem) is the atmospheric chemistry configuration of the CESM model and has been extensively validated and is highly customizable, making it well suited to study the behavior of new chemical species. In addition, a recently released version of CESM2 including emissions and reactions of short-lived halogen (SLH) species has made it possible to more accurately explore the effect of chlorine on VMS.^48^ Herein we describe the creation of a global atmospheric model of D4, D5, D6 and their oxidized byproducts within the CESM2-SLH version of CAM-chem. In addition, we provide concentrations and lifetimes of these species in the atmosphere as well as the effect of chlorine oxidation and temperature dependence predicted by this model. Finally, we show comparisons between model concentration estimates and VMS field measurements from the literature to assess the validity of the model and its input parameters.

## Methods

### Model Setup

A recently published version of the Community Earth System Model, CESM2-SLH, which included a complete update of SLH emissions and chemistry, was modified as described below.^48^ The concentrations of relevant non-VMS species in this model (particularly OH and Cl) have been extensively described and validated in previous work.^48–52^ Model cases were created using the FCnudgedvslslh configuration and f09_f09_mg17 (global, 0.9° (lat) × 1.25° (lon)) resolution. The model was comprised of 32 vertical levels between the surface and 40 km.^47^ Meteorological fields were nudged toward NASA’s MERRA-2 reanalysis for winds and temperature with 12-h relaxation time to ensure that the large-scale circulation remained consistent with observed meteorology.^53,54^ Three VMS species (D4, D5, and D6) and their oxidized byproducts were added to the model. Although oVMS consist of a wide array of products, oVMS were defined in the model as siloxanols (single OH substitution) as this was the primary product observed in chamber studies.^16^ Wet scavenging was represented in the model using a Henry’s law-based scavenging scheme for wet deposition, as described previously.^55,56^ Dry deposition was represented using the Wesely resistance parametrization employed by default for all species in CESM, which calculates a unidirectional flux to the surface based on meteorology, land-cover type, and compound specific parameters including the Henry’s law constant (*K*_*H*_) and the reactivity factor (*f0*) (Table S1).^47,56–58^ While this approach accounts for solubility and surface reactivity, it assumes irreversible uptake and therefore does not capture the significant bidirectional exchange that likely occurs for compounds such as VMS which have high air–water and low octanol-air partition coefficients.^25^ As a result, the predicted deposition rates in this model should be interpreted only as gross downward fluxes rather than the total net deposition. While this representation is sufficient for the purposes of this work (predicting atmospheric concentrations and lifetimes) given that deposition is a minor removal pathway (see the sensitivity tests described in the Supporting Information, p. 3), future studies may wish to employ a more sophisticated deposition framework.^59,60^ However, it must be noted that such a framework would require experimental data on the reactivity factor and vegetative uptake of VMS and the rate at which surface sinks are refreshed due to chemical transformation or physical turnover. These experimental values are unavailable or poorly constrained in the literature currently. Modified chemical mechanisms were created by modifying the default mechanism (i.e., chem_mech.in file). In these mechanisms, a single parent VMS (e.g., D5) reacted with a single hydroxyl or chlorine radical to form a single oVMS (Table S2). No other products were formed or consumed in the reaction to avoid perturbing the rest of the model chemistry. oVMS did not undergo further reactions to form secondary oxidation products and were only removed via wet and dry deposition. As a result, the modeled oVMS represented the bulk concentration of all oxidized VMS products. Both atmosphere and land model components were initialized using restart files from a previous simulation.^52^ In addition, the concentration of long-lived species (e.g., CO2, CH4, N2O, chlorofluorocarbons, halons, etc.) were prescribed using latitude-dependent lower boundary condition files from CMIP6.^47^

All model runs were 365 days, covering the year 2020, with a 60-day spin-up to establish initial conditions for VMS species. Sixty days was determined as a sufficient spin-up duration due to the relatively short lifetime of VMS and based on a sensitivity analysis of concentrations in remote areas (Figure S7). Four core model runs were conducted with all conditions kept constant between the models except for the chemical mechanism (Table S2). These runs and the associated chemical mechanisms were named according to their inclusion of hydroxyl radical reaction rate temperature dependence (T) and chlorine radical oxidation (CL). The first, “T:0,CL:0”, included only hydroxyl radical oxidation and did not use temperature-dependent reaction rates making it comparable to the mechanisms used in previous models. The second, “T:0,CL:1”, included both hydroxyl and chlorine radical oxidation but did not include temperature dependence. The third, “T:1,CL:0”, included only hydroxyl oxidation but used temperature-dependent reaction rates. Finally, “T:1,CL:1”, included both chlorine radical oxidation and temperature-dependent hydroxyl radical oxidation rates. Recently published temperature-dependent VMS-Cl reaction rates were not included in this work as they were not available at the time of the model development.^61^ However, compared to OH-VMS reactions, the variation in the rates of Cl-VMS reactions were relatively small. For example, the Cl-D5 reaction rates measured across the range of 273 to 353 K differed from the rate measured at room temperature by a maximum of 2%. Two additional test runs were conducted to assess the sensitivity of modeled VMS concentrations to the concentrations of halogens (Table S2). The first, “T:1,CL:1,SLH:0,” was equivalent to “T:1,CL:1” but excluded short-lived halogen emissions and chemistry. This test was designed to isolate the net impact of SLHs, which can both directly reduce the atmospheric burden of VMS by increasing chlorine radical concentrations and indirectly decrease it by reducing hydroxyl radical concentrations. The second test, “T:1,CL:0.01,” was also comparable to the “T:1,CL:1” case and retained full SLH chemistry but reduced the efficiency of the heterogeneous acid displacement reaction that converts HNO_3_ to HCl (het_ss_9), by modifying the reaction stoichiometry from 1 HNO_3_ → 1 HCl to 1 HNO_3_ → 0.01 HCl. This test was designed to evaluate the sensitivity of VMS concentrations to a lower modeled chlorine radical abundance.

### Emissions

Emissions were predicted assuming that consumer personal care product use was the only emissions source. Point sources such as manufacturing facilities, wastewater treatment plants, and landfills were not included as there was insufficient information on the location and size of these emissions. While personal care product use is believed to represent the overwhelming majority of the atmospheric releases of VMS, the global emissions estimates from this study consequently represent a lower limit.^62^ Emissions were generated at a one km^2^ resolution by multiplying 2020 population density by country-specific emissions rates.^63^ For the United States, the same per capita emissions rates used in J17 were selected.^36^ These values were chosen as they previously produced urban concentration estimates that showed strong agreement with field measurements in Chicago and New York City.^36–38^ Country-specific emissions rates for the rest of the world were calculated by normalizing the U.S. per capita emissions rates by each country’s 2020 per capita personal care product sales revenue (Table S3).^64^ For countries where personal care product sales data were not available, per capita emissions estimates were calculated by normalizing by per capita GDP.^65–74^ Emissions files at the one-degree model resolution were then compiled by averaging the calculated emissions of all one km^2^ grid cells that fell within each grid cell (Figure S1). The resulting total emissions for each compound were 2.5 × 10^7^ kg yr^–1^ (D4), 1.04 × 10^8^ kg yr^–1^ (D5), and 4.7 × 10^6^ kg yr^–1^ (D6).

## Data Analysis

### Analysis of Model Outputs

Near-surface atmospheric concentrations (hereafter referred to as “near-surface concentrations”) of VMS compounds and other chemical species were extracted from the lowest level of the model (surface to approximately 150 m). Tropospheric concentrations were calculated as pressure-weighted averages over all model levels at or below 200 hPa. Average global and regional concentrations were calculated using cosine (latitude) weighting to account for differences in grid cell areas. Deposition rates were saved as the integrated total column rate (kg m^–2^ s^–1^). The total column mass of each chemical species, *TC_g_* (kg m^–2^), was calculated by vertically summing the number of molecules within each discrete level of the model between the surface and the top of the atmosphere according to eq 1.^75^

1 $$TC_{g}=\sum x_{i}\times \frac{10\times \Delta P}{\phi \times M_{air}}\times M_{x}$$

In this equation, *x*_*i*_ is the volume mixing ratio (mol mol^–1^) of the given species within model level *i*, Δ*P* is the pressure differential (Pa) between the top and bottom of the model level, ϕ is the gravitational force (m s^–2^) for model level *i*, *M*_air_ is the molar mass of air (kg mol^–1^), and $M_{x}$ is the molar mass of the species (kg mol^–1^). Daily, seasonal, and annual atmospheric lifetimes for D4, D5 and D6 were calculated according to eq 2.

2 $$\tau_{i}=\frac{\overset{¯}{M_{VMS}\;}i}{\overset{¯}{R_{OH-VMS}\;}i+\overset{¯}{R_{CL-VMS}\;}i+\overset{¯}{WD_{VMS}}i+\overset{¯}{DD_{VMS}}i}$$

Here, *τ*_*i*_ is the average atmospheric lifetime (days) of the compound over the period of interest i , $\overset{¯}{M_{VMS}\;}i$ is the average mass of the VMS compound in the atmosphere over the same period, and $\overset{¯}{R_{OH-VMS}\;}i+\overset{¯}{R_{CL-VMS}\;}i+\overset{¯}{WD_{VMS}}i+\overset{¯}{DD_{VMS}}i$ are the average loss rates (kg day^–1^) to hydroxyl radical oxidation, chlorine radical oxidation, wet deposition, and dry deposition, respectively, during that period. Net transport (*T*) between “high emissions areas” and “low emissions areas” (as defined below) was calculated as the difference between total emissions to a given area and total losses due to both oxidation and deposition inside the given area, under the assumption that the annual net change in the atmospheric VMS burden was close to zero (i.e., that the system approximates to steady-state over the one-year averaging period for both regions) (eq 3).

3 $$T={E^{D5}}_{H}-{L^{D5}}_{H}=-\left({E^{D5}}_{L}-{L^{D5}}_{L}\right)$$

Here, *E* ^D5^ and *L* ^D5^ denote the total emissions and atmospheric losses (oxidation and deposition) of D5 which occur in or above the “high emissions” (subscript *H*) and “low emissions” (subscript *L*) areas.

### Comparison to Field Measurements

Twenty studies were identified that included measurements of at least one of the target compounds of interest (D4, D5, and D6) in outdoor air, included enough information to determine the location in which sampling was conducted, and included “non-industrial” study sites. Measured air concentrations were obtained from data published with the original studies or from personal correspondence with the authors if data were not publicly available.^23,33,34,37,38,76–91^ Study sites were only classified as “industrial” if the samples obtained specifically targeted a known point source or the authors reported that a point source was present in the vicinity of the study site. In total, these studies contained data from 184 study sites (Table S13). For the purposes of comparison, predicted VMS concentrations were extracted from the surface level of the “T:1,CL:1” model run from each grid cell containing the study sites. Since our model runs only included the year 2020, it was not possible to individually compare every data point in the literature to the model prediction for the exact time period it was collected. However, to capture seasonal concentration changes that would be expected from year to year, concentrations were extracted from the model over the calendar dates that the sampling was conducted (e.g., if sampling was conducted between May 15, 2017, and Jun 17, 2017, model data were extracted for May 15, 2020, to Jun 17, 2020). For data from active samples (typically deployed for 12–24 h), the measurement and model data from each site were grouped by season, averaged, and treated as a single paired comparison. For data from passive samplers, model data were extracted for the full period of the sample deployment, averaged, and then paired with the reported concentration value for that measurement since these passive samples were typically deployed for several months with a goal of capturing a seasonal average. Model-observation linear regressions were analyzed and the mean fractional bias (MFB, eq S1), mean fractional error (MFE, eq S2), normalized mean bias (NMB, eq S3), normalized mean error (NME, eq S4), and mean absolute percent error (MAPE, eq S5) were calculated.

## Results and Discussion

### Predictions of VMS Emissions, Concentrations, and Lifetimes

Considering the ongoing debate about whether the use of VMS compounds should be regulated at the state and national level, accurate predictions of the lifetimes and concentrations of these species in the environment are critically important.^31,32^ Based on current knowledge, we consider the results from the “T:1,CL:1” model, which includes both the effects of chlorine oxidation and the temperature dependence of OH oxidation, to be the most accurate reflection of these values out of our model cases. This model showed that predicted VMS emissions were highly localized, with 84% (8.8 × 10^7^ kg yr^–1^) of global D5 emissions occurring from the 6% of Earth’s surface area with a population density greater than 50 capita km^–2^. Only 4.1 × 10^7^ kg yr^–1^ of these emissions were lost to oxidation or deposition within these grid cells while the majority, 4.7 × 10^7^ kg yr^–1^, were transported to sparsely populated areas with low local emissions (Figure 1). Within these high emissions areas, the primary loss pathway was OH oxidation (2.8 × 10^7^ kg yr^–1^, 27% of global emissions), followed by deposition (9.0 × 10^6^ kg yr^–1^, 9% of global emissions) and Cl oxidation (3.8 × 10^6^ kg yr^–1^, 4% of global emissions). The total flux of D5 to the sparsely populated regions (<50 capita km^–2^) from transport (74%) and local emissions (26%) was 6.3 × 10^7^ kg yr^–1^. Within these regions, oxidation by OH (4.7 × 10^7^ kg yr^–1^, 45% of global emissions) was again responsible for the majority of the D5 loss while oxidation by chlorine (1.5 × 10^7^ kg yr^–1^, 14% of global emissions) was more impactful than deposition (4.1 × 10^6^ kg yr^–1^, 4% of global emissions). Within both the “high emission” and “low emissions” regions, deposition was comprised almost entirely (99%) of dry deposition and wet deposition was not a significant loss pathway. These results suggest that VMS concentrations in rural and unpopulated areas, including remote regions such as the Arctic (>66.5° latitude) and Antarctic (<−66.5° latitude), are primarily driven by emissions from a small number of densely populated regions but that a majority of VMS loss actually occurs downwind of these populous areas. Furthermore, they show that while the majority of VMS oxidation can be explained by OH, chlorine contributes a much larger share of oxidation (18% globally) than previously predicted.

**Figure 1:**
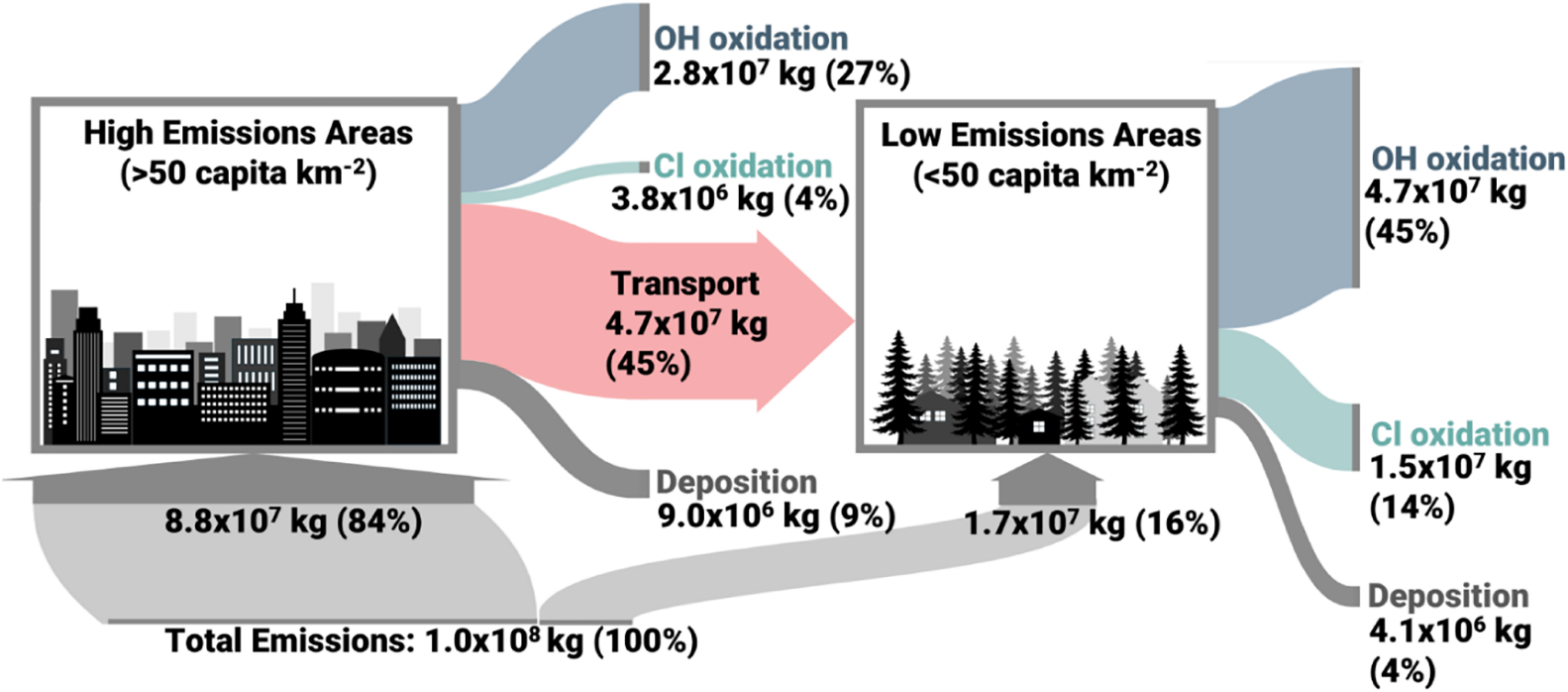
Flow diagram of D5 emissions, transport, and loss processes for the year 2020 in the “T:1,CL:1” model. The size of the flow lines and the accompanying values show the mass in kg (and as a percentage of total global emissions) of D5 emitted, transported, or lost via a given pathway over the course of the full year within all model levels. Emissions and subsequent loss and transport processes are separated into “high emissions” grid cells (where the average population density was greater than 50 capita km^–2^) and “low emissions” grid cells (where the average population density was less than 50 capita km^–2^).

The average global predicted near-surface concentrations of D4, D5, and D6 were 0.38, 1.6, and 0.077 ng m^–3^. The mean predicted near-surface concentration of D5 in the northern hemisphere (2.9 ng m^–3^) was an order of magnitude higher than in the southern hemisphere (0.31 ng m^–3^) because VMS lifetimes are not long enough for significant interhemispheric exchange to occur and approximately 87% of total D5 emissions occur in the northern hemisphere (Figure 2, Figures S1, S4, S5, S6, and Tables S8–S10).^92,93^ VMS concentrations exhibited large seasonal variations (Figure 2, Table 1, Figures S4, S5, S9, and Tables S8–10). For example, predicted D5 near-surface concentrations in the Northern and Southern Hemisphere were 63% and 48% lower in hemispheric summer (June–August and December–February, respectively) than in the hemispheric winter. Furthermore, the highest global average predicted near-surface concentration of D5 (2.9 ng m^–3^) and the highest predicted D5 near-surface concentrations at a single point (760 ng m^–3^, New York City) both occurred in January. These temporal trends can be explained by seasonal changes in the concentrations of hydroxyl radicals which peak during the summer months in both hemispheres (Table 1, Figure S3, and Table S6). Although the concentrations of chlorine radicals are also subject to seasonal changes (typically peaking in early spring), hydroxyl radicals contribute to the majority of VMS oxidation and thus it follows that their seasonal changes have the largest impact on VMS concentrations (Table 1).

**Table 1:** Global Concentrations and Lifetimes of VMS and Oxidative Species from the “T:1,CL:1” Model by Season

	Annual	Dec–Feb	Mar–May	Jun–Aug	Sep–Nov
Predicted D4 near-surface concentration (ng m^–3^)	0.38	0.57	0.40	0.25	0.30
Predicted D5 near-surface concentration (ng m^–3^)	1.6	2.4	1.7	1.1	1.3
Predicted D6 near-surface concentration (ng m^–3^)	0.077	0.12	0.078	0.053	0.062
Predicted D4 lifetime (Days)	6.7	9.8	5.5	4.3	7.1
Predicted D5 lifetime (Days)	4.0	6.2	3.1	2.4	4.3
Predicted D6 lifetime (Days)	2.7	4.4	2.1	1.6	2.9
Predicted tropospheric OH concentration (ppt)	0.077	0.071	0.073	0.090	0.075
Predicted tropospheric Cl concentration (ppt)	1.6 × 10^–4^	1.6 × 10^–4^	1.7 × 10^–4^	1.5 × 10^–4^	1.4 × 10^–4^

**Figure 2:**
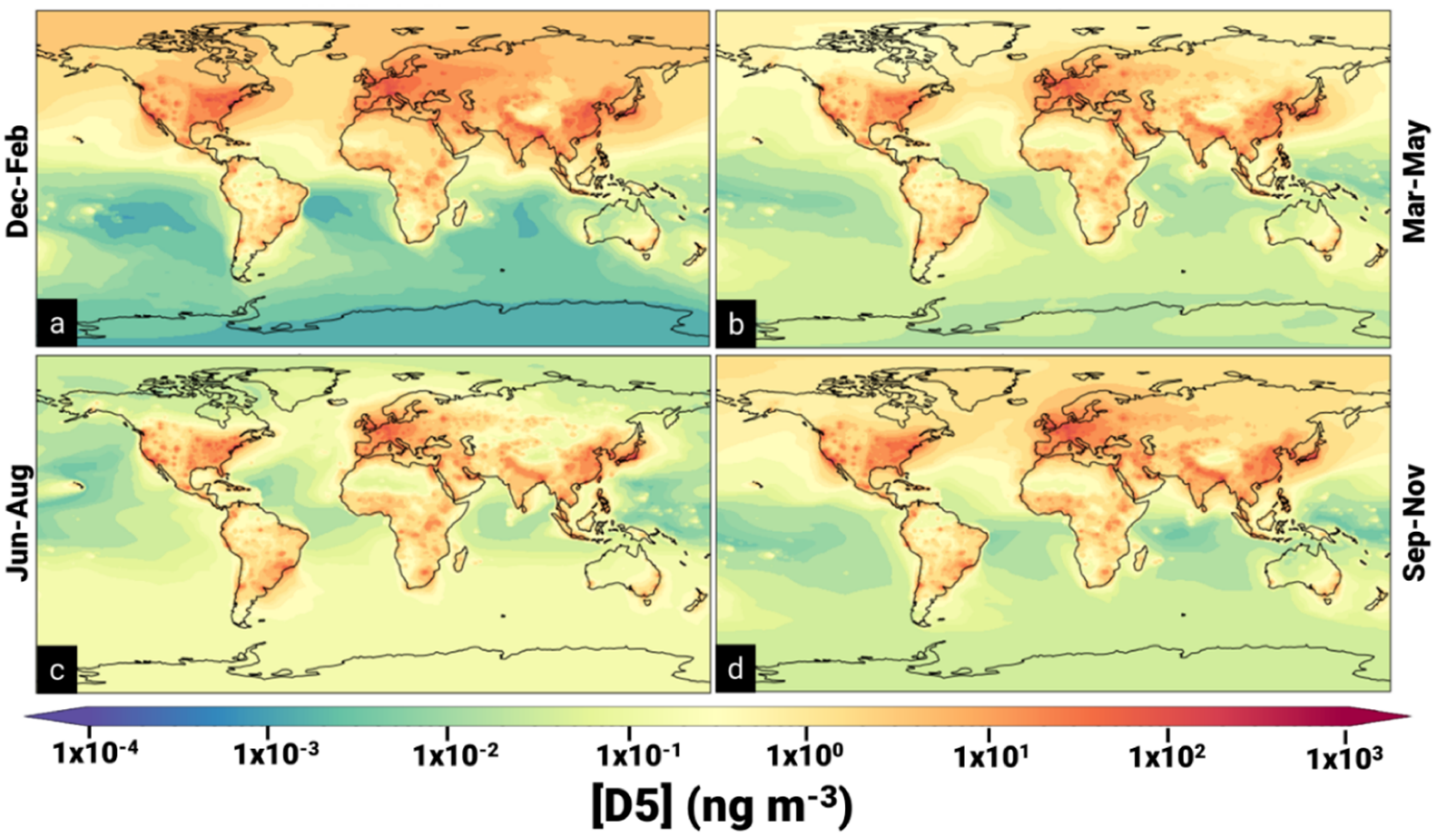
Average predicted seasonal near-surface concentrations of D5 from the “T:1,CL:1” model in ng m^–3^.

The calculated atmospheric lifetimes for D4, D5, and D6 were 6.7, 4.0, and 2.7 days (Table 1, Table S11). The difference in lifetimes between the three compounds is primarily a consequence of the fact that larger VMS both oxidize and deposit more quickly. Our predicted D5 lifetime was 47% lower than the estimate of 7.5 days which was previously calculated in “N11” without the assumption of temperature dependence, chlorine oxidation, or deposition. It was much closer (but still lower by 9%) to the estimate of 4.4 days which was previously calculated in Alton and Browne, 2020 with the assumption of both hydroxyl and chlorine radical oxidation but no temperature dependence.^35^ However, it should be noted that the tropospheric OH concentration in this work (0.077 ppt), N11 (∼0.041 ppt), and the calculations of Alton and Browne, 2020 (∼0.049 ppt) are not consistent.^13,35^ These discrepancies, rather than mechanistic differences, may account for a significant portion of the differences in calculated lifetimes. VMS lifetimes were also subject to large temporal variations (Table 1, Table S11). For example, the calculated global atmospheric lifetime of D5 was 6.2 days during the period of December to February compared to 2.4 days between June and August when OH concentrations in the northern hemisphere were at a maximum (Table S6, Figure S3).

While these lifetimes are shorter than some predicted in previous literature, they are still long enough for continental emissions to be transported to polar regions via “fast” pathways.^94–96^ Furthermore, the seasonal changes in lifetime suggest that there may be a significant increase in the quantity of VMS transported from urban regions to the poles and other remote regions during the winter. This LRET is supported by the fact that the average model-predicted D5 near-surface concentration of 1.6 ng m^–3^ in the Arctic is comparable to previous measurements, despite the fact that the area surrounded by the Arctic Circle contains only 0.1% of predicted global emissions.^78,80^ However, as those who have questioned the addition of VMS to the Stockholm Convention have pointed out, the presence of these chemicals in the polar atmosphere does not in itself warrant concern as it does not necessarily mean that they are deposited from the atmosphere in a significant quantity.^32^ After all, these chemicals are highly volatile and, as we have already shown, the primary pathway for their removal from the atmosphere is oxidation and not deposition. Our model predicted gross downward D5 fluxes of 1.3 and 0.021 μg m^–2^ yr^–1^ in the Arctic and Antarctic circles, respectively (Table S12). While these deposition fluxes were comparable to those of other chemicals known to undergo LRET such as polychlorinated biphenyls (0.13 to 0.73 μg m^–2^ yr^–1^) and mercury (6.8 to 7.8 μg m^–2^ yr^–1^) it must be highlighted that these results assume irreversible removal and do not account for revolatilization.^28,97–100^ As a result, these predicted rates cannot in themselves be interpreted as evidence of meaningful net accumulation in these regions which previous modeling has predicted to be relatively minor.^25^

While deposition is not the major removal pathway for VMS parent compounds, the byproducts produced in these oxidation reactions are typically far less volatile and are likely to be deposited in significant amounts. Given that our model treated these byproducts as a single species (while in reality VMS oxidation pathways are complex, multigenerational, and not yet fully characterized), the model at this time provides a highly uncertain estimate of the deposition of these oVMS.^16^ Explicit modeling of the oxidized byproducts is recommended to refine knowledge of the environmental fate of VMS. Refinements can build on the CESM implementation in this work as additional mechanistic, aerosol partitioning, and physical parameters become available.

### Impacts of Chlorine and Temperature Dependence

Neglecting to account for either the effect of temperature dependence or chlorine oxidation (as was done in a number of previous VMS models) is likely to introduce biases into predictions of VMS concentrations and lifetimes. However, it was impossible to gauge the exact direction and magnitude of these biases by comparing our results to previous modeling work, as observed discrepancies could be a result of a variety of methodological differences. The sensitivity runs in this work, holding other factors constant, permit isolation of the effects of the chlorine reactions and temperature-dependent OH reactions. When only the effect of adding chlorine oxidation to the temperature-invariant OH oxidation was considered (“T:0,CL:1” vs “T:0,CL:0”), the global predicted D5 near-surface concentration and atmospheric lifetime declined from 1.9 to 1.6 ng m^–3^ (16%) and from 5.2 to 3.3 days (36%) respectively (Figure 3, Tables S8–S11). These decreases were largest in regions with limited or no local emissions sources. For example, predicted D5 near-surface concentrations decreased by 5 to 16% in Europe, Asia, and the contiguous U.S. but decreased by 49% and 36% in the Arctic and Antarctic. Chlorine oxidation had the largest impact on global VMS concentrations during “Winter” in the Northern Hemisphere (Dec-Feb) when the Cl:OH ratio was at a maximum (Figure 3, Tables S8–S11). During this period, global predicted D5 near-surface concentrations declined by 25% relative to when no chlorine oxidation was present.

**Figure 3:**
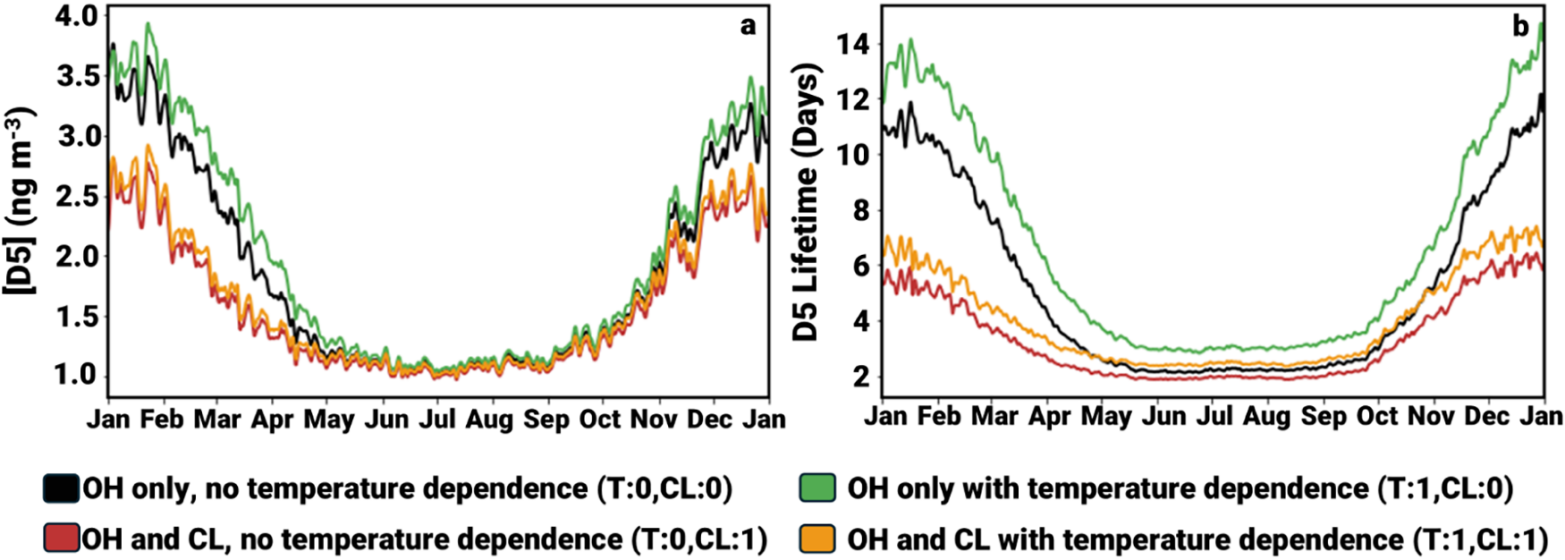
Time series of daily (a) (left) predicted global average D5 near-surface concentration (ng m^–3^) and (b) (right) predicted global D5 lifetime (days) from the four core model configurations. Tick marks indicate the first day of each month.

When only the effect of temperature dependence on OH oxidation was considered (with no chlorine oxidation present) (“T:0,CL:0” vs “T:1,CL:0”), the predicted D5 near-surface concentration and atmospheric lifetime increased from 1.9 to 2.0 ng m^–3^ (7%) and from 5.2 to 6.6 days (28%) respectively (Figure 3, Tables S8–S11). These increases can be explained by the fact that VMS are on average exposed to temperatures below 298 K and are thus oxidized more slowly when temperature dependence is considered. As with chlorine, the largest effects were observed in remote areas. Predicted D5 near-surface concentrations increased by 16% and 37% in the Arctic and Antarctic compared to a range of 4–8% in populated continental regions. These regional differences in the magnitude of change (for both effects) were likely because VMS compounds oxidize relatively slowly and air masses in these remote regions had more time to undergo oxidation. Furthermore, the Arctic and Antarctic are colder than these high emissions areas, which exacerbates the impact of temperature dependence. Temperature dependence had its largest effect between March and May when predicted D5 near-surface concentrations increased by 12% (Figure 3, Tables S8–S11).

These modeled differences demonstrate that failing to account for one of these factors in the prediction of VMS concentrations leads to meaningful enhancements or reductions of VMS concentrations and lifetimes. However, because these two factors are antagonistic (with temperature dependence generally increasing lifetimes and concentrations and chlorine decreasing them), examining their effects independently does not give us a clear picture of their combined impact. When both chlorine oxidation and temperature dependence were considered together (“T:1,CL:1” vs “T:0,CL:0”), the average predicted D4, D5, and D6 near-surface concentrations declined from 0.44 to 0.38, 1.9 to 1.7, and 0.089 to 0.077 ng m^–3^ respectively (Figure 3, Tables S8–S10). Likewise, these species’ atmospheric lifetimes declined from 8.0 to 6.7 days, 5.2 to 4.0 days, and 3.8 to 2.7 days (Table S11). While these decreases were smaller than observed in the model with only the addition of chlorine oxidation (T:0,CL:1), the increased loss due to chlorine outweighed the slower oxidation introduced by temperature dependence. While this is in part due to the relative magnitude of the two effects, it appears that there was some interaction between the two. For example, if the two effects were purely additive, we would expect predicted D5 near-surface concentrations to decline by only 0.16 ng m^–3^ when both were added to the model (the difference between the increase in the “T:1,CL:0” model and the decrease in the “T:0,CL:1” model) rather than the larger 0.24 ng m^–3^ decrease observed. We hypothesize that this may be due to the fact that densely populated urban areas where the majority of VMS emissions occur are frequently located in temperate climates and coastal regions and often have large anthropogenic and natural emissions of chlorine radical precursors (Figure S1).^101–106^ As a result, these large cities are typically warmer and have higher concentrations of chlorine radicals than the rest of the globe. This was observable in our model as grid cells with high population densities (>50 capita km^–2^) had higher than average near-surface temperatures (292 K vs 278 K) and predicted near-surface concentrations of chlorine radicals (5.6 × 10^–5^ vs 2.9 × 10^–5^ ppt). While, as we have previously noted, most VMS oxidation actually occurs outside of these areas, we hypothesize that the interaction we observed was a result of the fact chlorine was able to remove a meaningful portion of the emitted VMS in these urban areas (where temperature dependence has relatively little effect) before they were transported downwind. Furthermore, chlorine levels remain elevated in the warm oceanic and coastal regions surrounding these urban areas (which are not considered part of our “high emissions area”) due to efficient sea-salt aerosol recycling and a significant fraction of Cl-VMS oxidation may occur in the area immediately downwind of these emissions hotspots. As a result, the total mass of VMS that undergoes long-range transport, where temperature dependence exerts most of its influence on VMS oxidation, is reduced. Overall, these results suggest that while VMS lifetimes are still long enough for long-range transport to occur, some previously estimated lifetimes and concentrations available in literature may have been overestimates.

It must be noted that the size of the chlorine effect was obviously dependent on the concentrations of Cl radicals themselves. As noted previously, CESM2-SLH, which has been extensively described and validated in previous work, was selected for this purpose to provide the best representation of these concentrations (see Halogen and Radical Chemistry in the SI).^48–52^ However, there is still some uncertainty associated with these concentrations since they were computed rather than prescribed and because Cl concentrations in the 0.9 × 1.25° resolution used for this work may be biased high relative to the 1.9 × 2.5° resolution used for the validation of CESM2-SLH. As a result, it is possible that the magnitude of the chlorine impact was overestimated in the T:0,CL:1 and T:1,CL:1 model cases. However, we found that when the concentrations of chlorine radicals were lowered in the T:1,CL:0.01 model case, the impact on VMS concentrations was generally small. While the global tropospheric concentration of chlorine radicals was 19% lower in T:1,CL:0.01 than in T:1,CL:1, the global D5 predicted near-surface concentration and lifetime increased by only 3% and 8% (Figures S2 and S8, Tables S7–S11). While larger differences in VMS concentrations and lifetimes were observed for certain regions and time periods (in particular polar regions during their hemispheric springs), they were still almost universally lower than those in the T:0,CL:0 model (Tables S8–S10). The tropospheric OH concentration (which can be indirectly affected by the concentrations of Cl radicals as discussed below) in the T:1,CL:1 case (0.077 ppt) was not meaningfully different when the abundance of chlorine radicals was lowered in the T:1,CL:0.01 case (0.078). However, this tropospheric OH concentration was meaningfully higher than the range of estimates calculated in previous work (0.030 to 0.054 ppt) (Table S6).^107,108^ As a result, while these results provide a robust prediction of the relative changes in cVMS concentrations and lifetimes due to added chlorine and temperature effects, there is still significant uncertainty associated with their chemical losses to OH (which are well-known to be strongly model dependent) and consequently the absolute magnitude of these lifetime and concentration values.^109^ Overall, these results demonstrate that even if Cl concentrations in the T:1,CL:1 model were biased high, the effect of chlorine oxidation was still dominant over the contrasting effect of temperature-dependent OH reactions.

### Direct and Indirect Impacts of Short-Lived Halogens

While the use of short-lived halogen chemistry in these models directly increased the calculated chlorine-driven oxidation of D4, D5, and D6, these additional halogen species may have also indirectly affected the oxidation of VMS by hydroxyl radicals. Halogen radicals react with O_3_, the primary source of hydroxyl radicals in nonpolluted environments with low concentrations of nitrogen oxides and VOCs.^49,110,111^ As a result, the modeled concentration and oxidative impact of hydroxyl radicals are reduced in these regions when additional halogens are present.^109^ This indirect effect on the lifetime of atmospheric trace species has been explored for methane, where the indirect effect (lower OH) outweighed the direct contribution to methane destruction from additional halogens.^49^ However, based on relative reactions rates (*k*_*OH+CH4*_:*k*_*Cl+CH4*_ = 0.26, *k*_*OH+D5*_:*k*_*Cl+D5*_ = 0.012), we expected the size of this indirect effect to be much smaller for VMS species than for methane.^13,49^ To confirm that the direct effect of SLH outweighed this indirect effect, we compared the modeled VMS concentrations and lifetimes produced when these short-halogen species were present in the model (“T:1,CL:1”) as opposed to when they were excluded (“T:1,CL:1,SLH:0). As expected, the average tropospheric concentration of OH for 2020 decreased by 10% (0.086 vs 0.077 ppt) when the short-lived halogens were added to the model (Figure 4, Table S6). However, due to the additional emissions of chlorine-containing species, the tropospheric concentration of chlorine radicals increased (2.4 × 10^–5^ to 1.6 × 10^–4^ ppt) by almost an order of magnitude (Figure 4, Table S7). These factors combined to decrease the average predicted near-surface concentration and atmospheric lifetime of D5 by 16% (1.9 vs 1.6 ng m^–3^) and 33% (5.9 vs 4.0 days) respectively (Figure 4, Tables S8–S11). In other words, the direct impact of the additional chlorine greatly outweighed the indirect effect on hydroxyl radicals. However, this decrease is not as large as would have been predicted if only the direct oxidative effect of short-lived chlorine species were considered. This finding highlights the need to consider both the direct and indirect impact of these short-lived halogen species when predicting the atmospheric fate of VMS and other volatile compounds. Future research studying the oxidative impacts of SLH using prescribed halogen concentrations from this work or others must ensure that they either also prescribe hydroxyl radical concentrations that account for this indirect effect or implement a complete SLH scheme as described in Fernandez et al., 2025.^48^ These results also demonstrate the need to explicitly model these effects as this work and others such as Bossolasco et al. 2025 and Li et al. 2022 have now shown cases where the indirect effect can dominate (e.g., methane) and other cases where the direct effect can dominate (e.g., VMS).^49,109^

**Figure 4:**
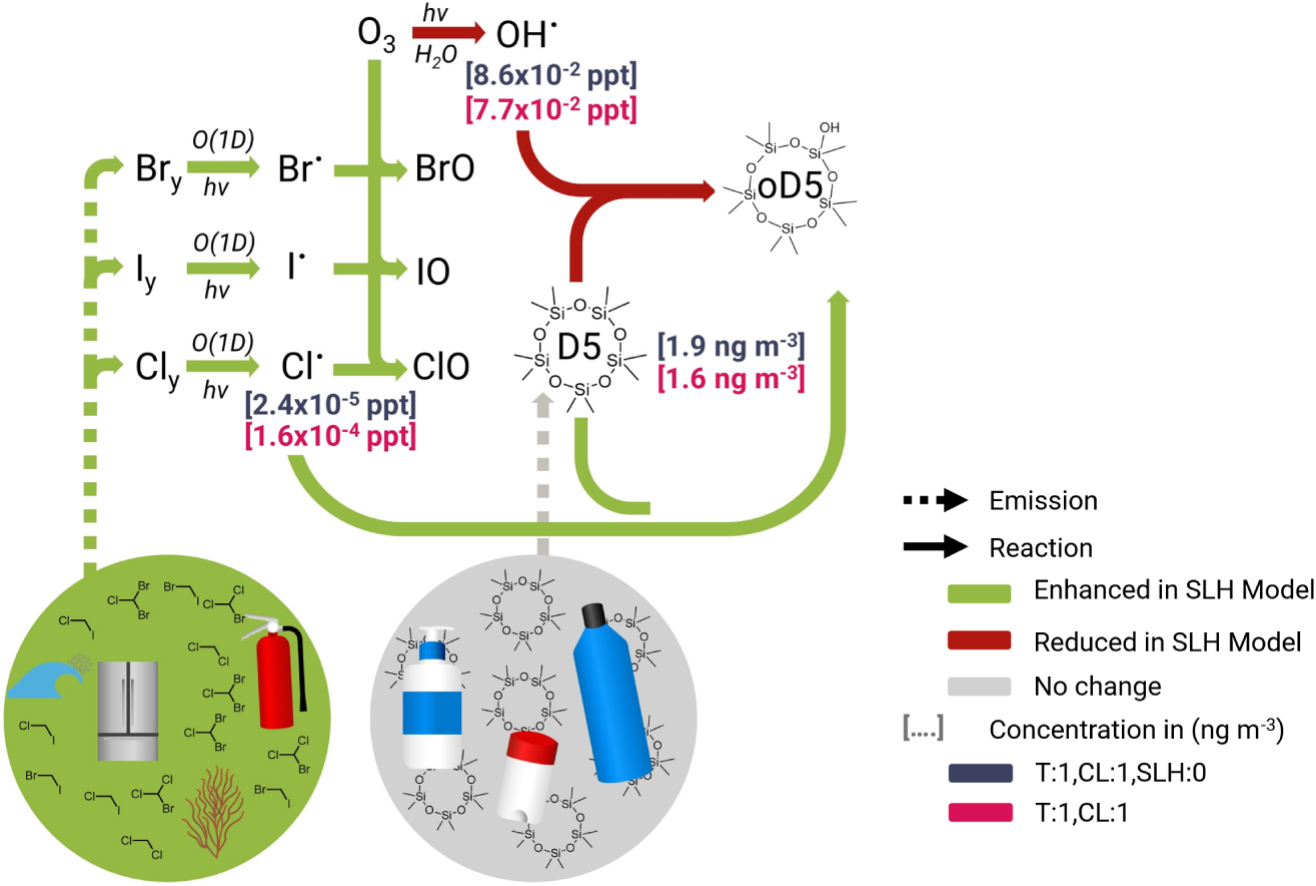
Flow diagram depicting the effect of SLHs on the predicted tropospheric concentration of Cl and OH and the subsequent net impact on the predicted near-surface concentration of D5.

### Model Comparison to Measurement Data

There is a growing database of global VMS concentration measurements available for model evaluation (Table S13). This was especially important because our model outputs were based entirely on predicted VMS emissions, reactivity, and transport and were not fitted or adjusted to match any measurement data prior to their analysis. Model skill for seasonal concentration patterns and remote locations are particularly important for evaluating the accuracy of the enhancements for temperature dependence and Cl oxidation. Measurements from active samplers compared relatively better with T:1,CL:1 model predicted near-surface concentrations of D5 (*R* ^2^=0.56, N = 179) and D6 (*R* ^2^=0.54, N = 170) than for D4 (*R* ^2^=0.41, N = 174) when evaluated on a log–log basis (Figure 5, Table S17). The relatively weaker relationship for D4 may be due to the fact that a large portion of emitted D4 is attributed to its industrial uses (such as polymerization) rather than the use of personal care products (which was the only factor considered in our emissions predictions).^62^ Model predictions of active sampler measurements showed a negative bias with mean concentrations systematically lower than observed values (Table S17). This was particularly true for densely populated areas with high measured concentrations of VMS such as Barcelona, New York City, and Saitama. This is likely due to the inability of the model to represent peak urban concentrations at 1 degree model resolution, a well-known error in low resolution models.^112^ This was also documented in J17 where higher resolution models had better performance for cVMS at urban sites than lower resolution models.^36^ There were two notable clusters of active measurements which differed from the model prediction by more than an order of magnitude. The first (Cluster A, Figure 5) was a set of measurements from Hanoi, Vietnam which our model overpredicted by more than an order of magnitude.^88^ The second (Cluster B, Figure 5) was a set of measurements from large cities in China (Beijing, Kunming, LiJian, and Zhangjiagang) which were underpredicted by an order of magnitude.^77^ While there is no definitive explanation for these outlier groups, they suggest that our estimates of per capita emissions for these countries may have been too high (in the case of Vietnam) or too low (in the case of China) or that there are important industrial sources in the measurement region which were not reflected in our emissions estimates.^113^

**Figure 5:**
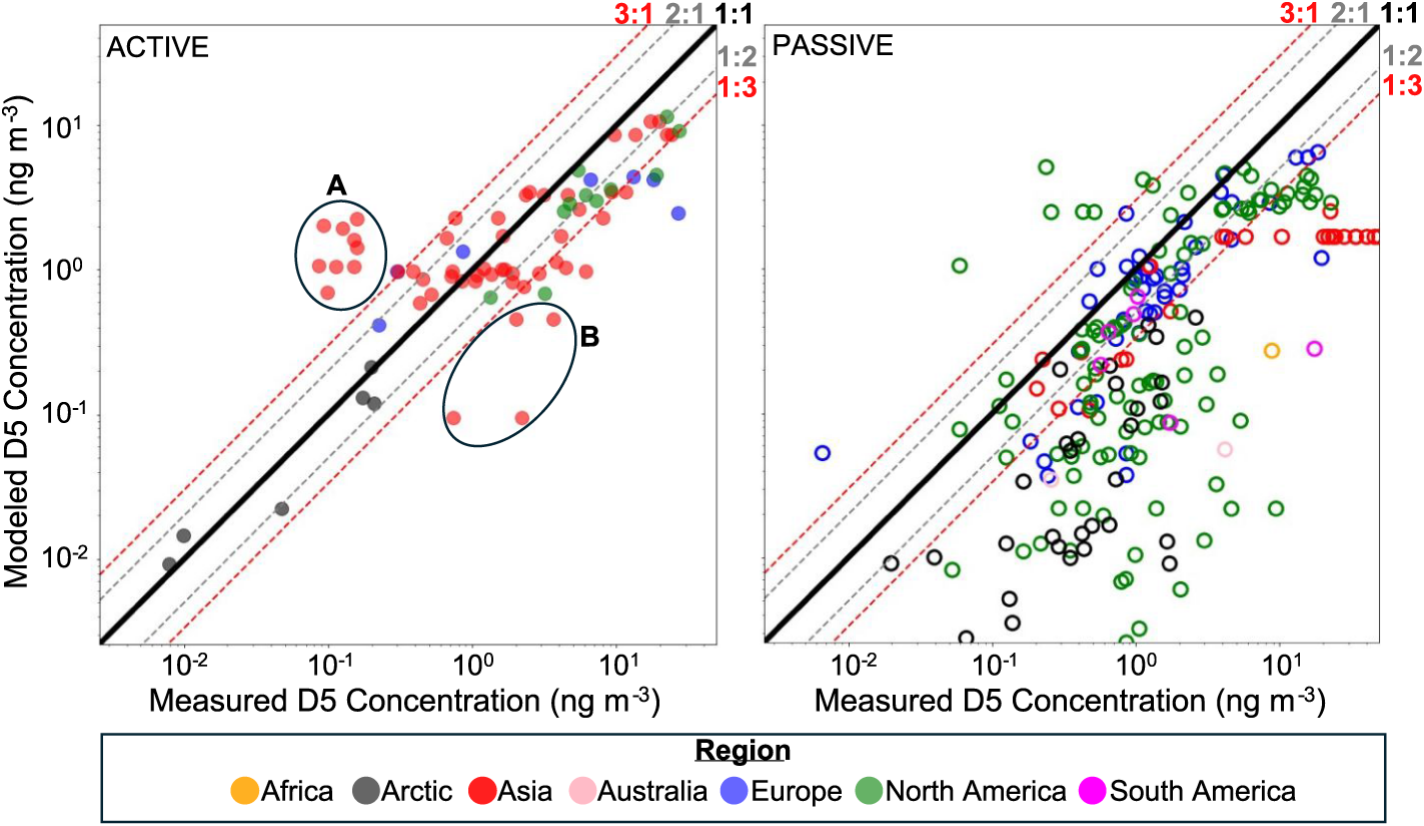
Near-surface concentrations of D5 predicted by the “T:1,CL:1” model as a function of measured concentrations from active samples (left) and passive samples (right). Each dot represents a single paired comparison with the *y*-axis indicating the mean of the log transformed modeled concentrations for the period of interest and the *x*-axis indicating the mean of the log transformed measured concentrations at each site. The region of each site is represented by the color of the dots as indicated in the legend. The solid black line indicates a perfect 1:1 relationship while the dotted gray and red lines indicate the 2:1 and 1:2 relationships and 3:1 and 1:3 relationships, respectively. Circled regions A and B represent two sets of active measurements which showed particularly poor agreement with the model as noted in the text.

The relationship between T:1,CL:1 model results and measurements from passive samplers on a log–log basis was much weaker for all 3 compounds with an *R* ^2^ of 0.38 for D5, 0.37 for D6, and only 0.04 for D4 (Figure 5, Table S17). Furthermore, the degree of model underprediction was much greater for passive sampler data than for with active samples. For example, the NMB for passive measurements of D5 was −0.76 as opposed to only −0.50 for active measurements. While we do not have clear explanation for why the model did a markedly worse job of predicting these passive measurements, we note that it would be impossible for any single-year model run to predict every passive measurement available in the literature to within an order of magnitude. This is because passive concentration measurements reported from the same study site and season (but different measurement years) frequently differed from each other by an order of magnitude or more. For example, the passive measurements of D5 from three studies from approximately the same time of year in Košetice, Czechia ranged between 8.2 and 297 ng m^–3^. This variability in the reported concentrations was frequent enough that a large degree of either overestimation or underestimation was unavoidable. Approximately 40% of all sites with passive measurement data were sampled by multiple studies and, of these, half had measurements which differed by an order of magnitude or more. While it is possible that this variability is due to real year-to-year variations in VMS concentrations at certain sites, an alternate model run done for 2018 (not shown) had nearly identical (*R* ^2^ = 0.97) concentrations relative to the 2020 case. While it is unclear why the disagreement between passive measurements and model predicted concentrations generally trended toward underprediction rather than overprediction, large negative biases (NMB up to −0.63) have been observed in previous comparisons of passive sampler measurements and atmospheric transport models for other chemicals.^114,115^ We also note that while an alternate model configuration might align more closely with these passive measurements (which were underpredicted by the T:1,CL:1 model), it would also likely overpredict the active measurements which our current model configuration shows relatively good agreement with. For example, while the concentrations predicted by the T:1,CL:0 and T:0,CL:0 models had smaller negative biases and lower MAPEs when compared to passive measurements, they had lower R^2^s and higher MAPEs when compared to active measurements (Tables S14 and S15). However, the opposite was true for the T:0,CL:1 model, and in general the differences in the prediction performance of the four model configurations were small (Tables S14–17). To further illustrate the variability present in this data, we have included time series comparisons (based on calendar date) of model outputs and measurement data from all sites where measurement data were reported by at least three studies (Figure 6, Figures S12–S22). The selected comparisons below from Toronto and Svalbard demonstrate the close alignment between the range of concentrations present in some of these samples and the simultaneous underprediction of others.

**Figure 6:**
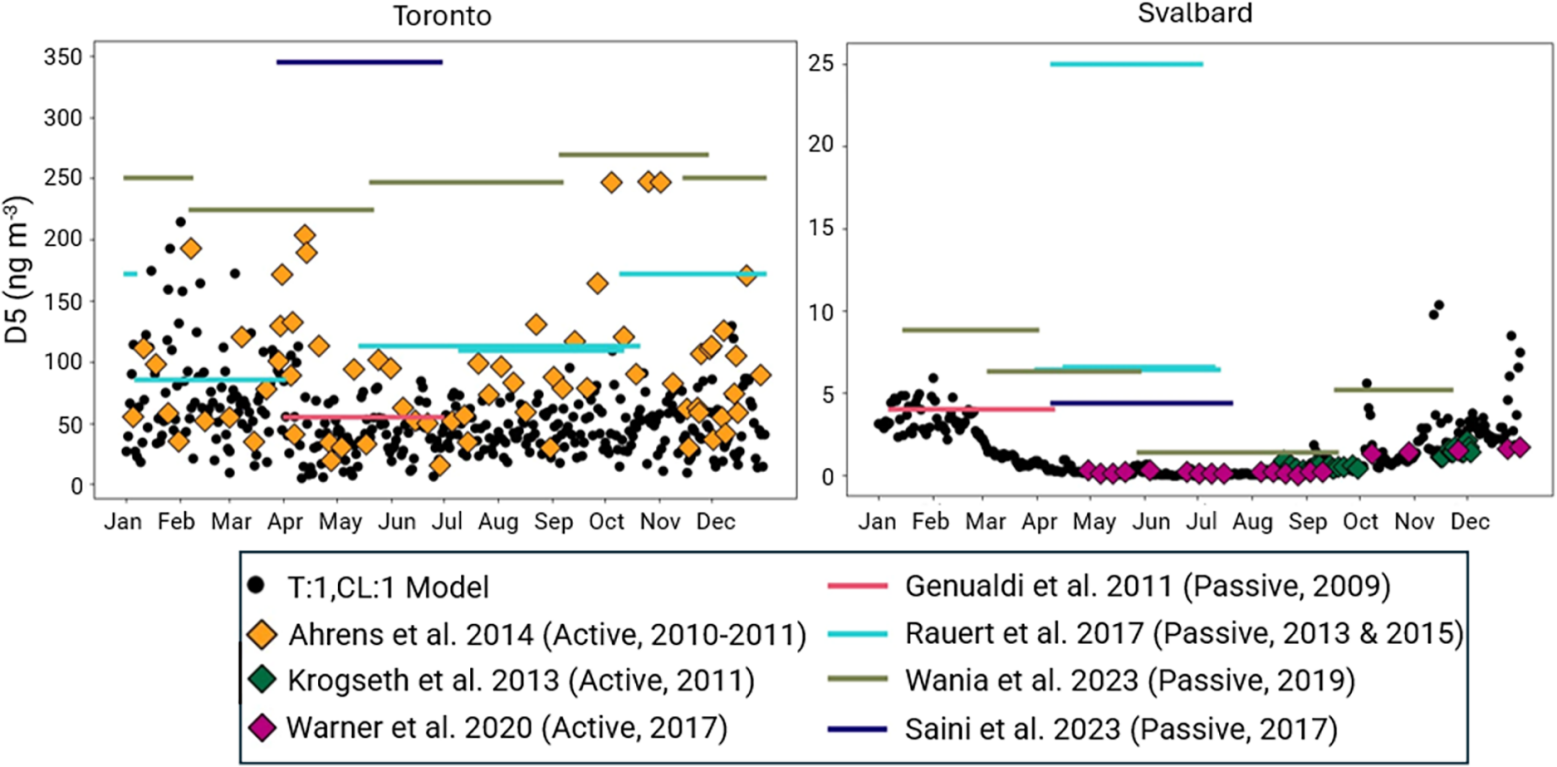
Seasonal time series comparison of D5 concentrations from the T:1,CL:1 model and measurements reported in the literature from Toronto, Ontario, Canada (left) and Svalbard, Norway (right).^33,78–81,83,90^ Model data and active measurements are plotted as points while passive measurements are plotted as solid bars representing the date range that samplers were deployed. Data sources are shown in the legend as the study name followed by the sampling method used and the years that sampling was conducted as (Method, Year). Note that data are plotted based only on their calendar date (e.g., data from 1/6/2015 and 1/6/2017 are plotted at the same point on the *X*-axis.)

## Supplementary Material



## Data Availability

The data underlying this study are openly available in Iowa Research Online at 10.25820/data.007521.

## Notes

Selected model input parameters, regional concentrations of VMS, halogen, and radical species from varying model configurations, seasonal maps of VMS concentrations, time series of VMS concentrations during model spin-up, time series of comparisons between model outputs and measurement data from the literature, and instructions for building and running the models described (PDF)
