# Supplementary material for: Modeled Global Impacts of Chlorine Oxidation and Temperature Dependence on the Atmospheric Lifetime and Concentrations of Volatile Methyl Siloxanes
Source: Environ Sci Technol. 2025 Dec 17;60(1):873–86. doi: 10.1021/acs.est.5c08897 (PMC12810245; doi:10.1021/acs.est.5c08897)
Supplement: Supplementary file 1 [file es5c08897_si_001.pdf]

Supporting Information for Modeled Global Impacts of Chlorine Oxidation and Temperature Dependence on the Atmospheric Lifetimes and Concentrations of Volatile Methyl Siloxanes

Christopher E. Brunet[1,2], Saeideh Mohammadi[2,3], Behrooz Roozitalab[4], Nora K. Gibson[1], Rafael P. Fernandez[5], Alfonso Saiz-Lopez[6], Keri C. Hornbuckle[1,2], Charles O. Stanier*[2,3]

[1]Department of Civil and Environmental Engineering, University of Iowa, Iowa City, 52242, United States of America
[2]IIHR-Hydroscience and Engineering, University of Iowa, Iowa City, Iowa 52242, United States
[3]Department of Chemical and Biochemical Engineering, University of Iowa, Iowa City, 52242, United States of America
[4]National Center for Atmospheric Research, Boulder, 80301, United States of America
[5]Institute for Interdisciplinary Science (ICB), National Research Council (CONICET), FCEN-UNCuyo, Mendoza 5501, Argentina.
[6]Department of Atmospheric Chemistry and Climate, Institute of Physical Chemistry Blas Cabrera, CSIC, Madrid, 28006, Spain

**Correspondence to*: Charles O. Stanier (charles-stanier@uiowa.edu)

The data underlying this study are openly available in Iowa Research Online at 10.25820/data.007521

Number of pages: 46

Number of tables: 18

Number of figures: 22

Table of Contents

Modeling Parameters.. 3

Halogen and Radical Chemistry 12

VMS Chemistry.. 19

Model Comparison to Measurement Data 29

Instructions For Running the Model. 41

Modeling Parameters

A full list of selected modeling values can be found in Table S1. VMS and oVMS Henry's law coefficients were estimated using HENRYWIN (v3.21) updated with the new EPI Suite files released in 2017.[1] Enthalpies of vaporization (ΔHvap) for D4, D5, and D6 were obtained from previously reported experimental data.[2, 3] Predicted ΔHvap for oD5 and oD6 were obtained from ChemSpider records of previously generated predictions (Advanced Chemistry Development, Inc. PhysChem Module, version: 14.00).[4-6] ΔHvap for oD4 was not available and was estimated by multiplying the experimentally determined value for D4 by the ΔHvap ratio of oD5 and D5. Non-temperature-dependent rate coefficients for hydroxyl and chlorine radical oxidation were obtained from previously published relative rate analyses with methyl ethyl ketone as the reference compound.[7] The Arrhenius rate parameters were derived from a prior laboratory study.[8] To capture an upper limit of the effect of temperature dependence, the largest reported activation energy (Ea) was selected and used for all three VMS compounds. The Arrhenius pre-exponential factor (A) was then selected such that at 298 K, the temperature-dependent rates were equal to the non-temperature-dependent rates. The reactivity factor (f0), which governs the loss of the chemical after entering plant stomata (and thus the rate of dry deposition), was set to 0.1 for all VMS and oVMS. This value was selected because dry deposition rates of other VOCs modeled using the Wesley parameterization employed in CESM and f0=0.1 have shown relatively good agreement with measured values.[9] To quantify the uncertainty introduced to the model by the selection of this modeling parameter, four 5-day sensitivity tests were performed using the "T:1,CL:1" VMS chemistry with one case having no deposition and the other three including both wet and dry deposition and with f0 alternately set to 0, 0.1, and 1. Across the range of tested reactivity factors, modeled deposition ranged between 8.8×10^{-3} and 1.8×10^{-1} kg km-2 yr-1 (dry) and 6.9×10^{-10} and 9.4×10^{-10} 1 kg km^{-2} yr^{-1} (wet). While there was more than an order of magnitude difference in total deposition between the f0=0.0 and f0=1.0 cases, the average predicted D5 concentration in f0=1.0 case was only 5% lower than in the f0=0.0 case. The results from these tests indicated that while the selection of the reactivity factor had a significant impact on the predicted deposition rates, it did not meaningfully impact the predicted concentrations of VMS compounds. It is worth noting that the updated version of HENRYWIN (which included organosilicon data for the first time) did not become available until after the creation of J17.[10] As a result, the Henry's law constants for the oVMS compounds we used are two orders of magnitude larger than those used previously. However, the modeled Henry's law constants of the VMS parent compounds we selected are within one order of magnitude of those used in J17 (which were obtained from experimental data). In addition, the non-temperature-dependent hydroxyl radical oxidation rates in our model were not published until 2020 and are approximately 30% faster than those used in J17.

Table S1: Selected modeling parameters

Species	Parameter	Value	Units
D4	Henry's Law Constant (KH_{298})[1]	$5.5x10^{-5}$	M atm^{-1}
D5	Henry's Law Constant (KH_{298})[1]	$2.4x10^{-5}$	M atm^{-1}
D6	Henry's Law Constant (KH_{298})[1]	$1.0x10^{-5}$	M atm^{-1}
OD4	Henry's Law Constant (KH_{298})[1]	$1.0x10^{0}$	M atm^{-1}
OD5	Henry's Law Constant (KH_{298})[1]	$4.6x10^{-1}$	M atm^{-1}
OD6	Henry's Law Constant (KH_{298})[1]	$2.0x10^{-1}$	M atm^{-1}
D4	Enthalpy of Vaporization (ΔH/R)[2]	6682	K
D5	Enthalpy of Vaporization (ΔH/R)[2]	7096	K
D6	Enthalpy of Vaporization (ΔH/R)[3]	7530	K
OD4	Enthalpy of Vaporization (ΔH/R)[4]	6570	K
OD5	Enthalpy of Vaporization (ΔH/R)[4]	6916	K
OD6	Enthalpy of Vaporization (ΔH/R)[4]	7601	K
D4	Molecular Weight	296.08	g mol^{-1}
D5	Molecular Weight	370.09	g mol^{-1}
D6	Molecular Weight	444.11	g mol^{-1}
OD4	Molecular Weight	298.05	g mol^{-1}
OD5	Molecular Weight	372.07	g mol^{-1}
OD6	Molecular Weight	446.09	g mol^{-1}
D4	Reactivity Factor (f_0)	0.1	Unitless
D5	Reactivity Factor (f_0)	0.1	Unitless
D6	Reactivity Factor (f_0)	0.1	Unitless
OD4	Reactivity Factor (f_0)	0.1	Unitless
OD5	Reactivity Factor (f_0)	0.1	Unitless
OD6	Reactivity Factor (f_0)	0.1	Unitless
D4	Hydroxyl Radical Reaction Rate (K_{OH})[7]	$1.30x10^{-12}$	cm^3 $molecule^{-1}$ s^{-1}
D5	Hydroxyl Radical Reaction Rate (K_{OH})[7]	$2.20x10^{-12}$	cm^3 $molecule^{-1}$ s^{-1}
D6	Hydroxyl Radical Reaction Rate (K_{OH})[7]	$3.20x10^{-12}$	cm^3 $molecule^{-1}$ s^{-1}
D4	Chlorine Radical Reaction Rate (K_{CL})[7]	$1.14x10^{-10}$	cm^3 $molecule^{-1}$ s^{-1}
D5	Chlorine Radical Reaction Rate (K_{CL})[7]	$1.80x10^{-10}$	cm^3 $molecule^{-1}$ s^{-1}
D6	Chlorine Radical Reaction Rate (K_{CL})[7]	$2.60x10^{-10}$	cm^3 $molecule^{-1}$ s^{-1}
D4	Hydroxyl Radical Pre-Exponential Arrhenius Term (A) [Δ]	$2.33x10^{-11}$	cm^3 $molecule^{-1}$ s^{-1}
D5	Hydroxyl Radical Pre-Exponential Arrhenius Term (A) [Δ]	$3.93x10^{-11}$	cm^3 $molecule^{-1}$ s^{-1}
D6	Hydroxyl Radical Pre-Exponential Arrhenius Term (A) [Δ]	$5.72x10^{-11}$	cm^3 $molecule^{-1}$ s^{-1}
D4	Hydroxyl Radical Exponential Arrhenius Term ($-E_a$)[8]	-859	K
D5	Hydroxyl Radical Exponential Arrhenius Term ($-E_a$)[8]	-859	K
D6	Hydroxyl Radical Exponential Arrhenius Term ($-E_a$)[8]	-859	K

Δ: Iteratively solved so that at 298k the temperature dependent reaction rate was equal to the non-temperature dependent reaction rate.

Table S2: Model Configurations and Reaction Expressions

Model Configuration	Short-Lived Halogen Emissions+Chemistry	VMS Reactions	[het_ss_9] stoichiometry
T:0,CL:0	Included	D4 + OH· → OD4; $k=1.3x10^{-12}$<br>D5 + OH· → OD5; $k=2.2x10^{-12}$<br>D6 + OH· → OD6; $k=3.2x10^{-12}$	HNO_3 → HCl
T:1,CL:0	Included	D4 + OH· → OD4; $k=2.33x10^{-11}\ e^{(-859/RT)}$<br>D5 + OH· → OD5; $k=3.93x10^{-11}\ e^{(-859/RT)}$<br>D6 + OH· → OD6; $k=5.72x10^{-11}\ e^{(-859/RT)}$	HNO_3 → HCl
T:0,CL:1	Included	D4 + OH· → OD4; $k=1.3x10^{-12}$<br>D5 + OH· → OD5; $k=2.2x10^{-12}$<br>D6 + OH· → OD6; $k=3.2x10^{-12}$<br>D4 + Cl· → OD4; $k=1.14x10^{-10}$<br>D5 + Cl· → OD5; $k=1.8x10^{-10}$<br>D6 + Cl· → OD6; $k=2.6x10^{-10}$	HNO_3 → HCl
T:1,CL:1	Included	D4 + OH· → OD4; $k=2.33x10^{-11}\ e^{(-859/RT)}$<br>D5 + OH· → OD5; $k=3.93x10^{-11}\ e^{(-859/RT)}$<br>D6 + OH· → OD6; $k=5.72x10^{-11}\ e^{(-859/RT)}$<br>D4 + Cl· → OD4; $k=1.14x10^{-10}$<br>D5 + Cl· → OD5; $k=1.8x10^{-10}$<br>D6 + Cl· → OD6; $k=2.6x10^{-10}$	HNO_3 → HCl
T:1,CL:1,SLH:0	Not Included	D4 + OH· → OD4; $k=2.33x10^{-11}\ e^{(-859/RT)}$<br>D5 + OH· → OD5; $k=3.93x10^{-11}\ e^{(-859/RT)}$<br>D6 + OH· → OD6; $k=5.72x10^{-11}\ e^{(-859/RT)}$<br>D4 + Cl· → OD4; $k=1.14x10^{-10}$<br>D5 + Cl· → OD5; $k=1.8x10^{-10}$<br>D6 + Cl· → OD6; $k=2.6x10^{-10}$	Not Included
T:1,CL:0.01	Included	D4 + OH· → OD4; $k=2.33x10^{-11}\ e^{(-859/RT)}$<br>D5 + OH· → OD5; $k=3.93x10^{-11}\ e^{(-859/RT)}$<br>D6 + OH· → OD6; $k=5.72x10^{-11}\ e^{(-859/RT)}$<br>D4 + Cl· → OD4; $k=1.14x10^{-10}$<br>D5 + Cl· → OD5; $k=1.8x10^{-10}$<br>D6 + Cl· → OD6; $k=2.6x10^{-10}$	HNO_3 → 0.01*HCl

Table S3: Country specific, per-capita emissions rate (PCER) and per-capita personal care (PC-PCP) product sales revenue for 2020.

Country	Country Code	PC-PCP Revenue (USD)	D4 PCER (mg day^{-1})	D5 PCER (mg day^{-1})	D6 PCER (mg day^{-1})	GDP (USD)
Afghanistan	afg	22.9*	6.1	25.1	1.2	512
Aland Islands	ala	106*	28.2	116	5.3	55829
Albania	alb	38.3	10.2	41.9	1.9	5343
Algeria	dza	24.0*	6.4	26.3	1.2	3354
American Samoa	asm	45.6*	12.1	49.9	2.3	15610
Andorra	and	78.0*	20.7	85.4	3.9	37207
Angola	ago	19.2*	5.1	21	1.0	1451
Anguilla	aia	47.0*	12.5	51.4	2.4	16538
Antigua and Barbuda	atg	45.0*	12.0	49.3	2.3	15225
Argentina	arg	39.6	10.5	43.3	2.0	8501
Armenia	arm	30.1	8.0	32.9	1.5	4506
Aruba	abw	58.2*	15.5	63.7	2.9	24008
Australia	aus	92.1	24.5	101	4.6	51868
Austria	aut	112	29.7	122	5.6	48789
Azerbaijan	aze	31.0	8.2	33.9	1.6	4230
Bahamas	bhs	58.2*	15.5	63.7	2.9	23998
Bahrain	bhr	62.6	16.7	68.5	3.1	23433
Bangladesh	bgd	25.6	6.8	28.1	1.3	2233
Barbados	brb	47.5*	12.6	52	2.4	16883
Belarus	blr	30.0	8.0	32.8	1.5	6543
Belgium	bel	83.7	22.3	91.7	4.2	45609
Belize	blz	31.6	8.4	34.6	1.6	5185
Benin	ben	21.8	5.8	23.9	1.1	1241
Bermuda	bmu	184*	48.9	201	9.2	107792
Bhutan	btn	28.8	7.7	31.5	1.4	3181
Bolivia	bol	27.6	7.3	30.2	1.4	3069
Bonaire, Sint Eustat	bes	57.7*	15.4	63.2	2.9	23700
Bosnia-and-Herzegovina	bih	35.5	9.4	38.9	1.8	6095
Botswana	bwa	31.9	8.5	35	1.6	5875
Brazil	bra	52.0	13.8	56.9	2.6	6924
British Virgin Islands	vgb	94.6*	25.2	104	4.8	48273
Brunei Darussalam	brn	39.0	10.4	42.7	2.0	27179
Bulgaria	bgr	37.0	9.8	40.5	1.9	10148
Burkina Faso	bfa	20.6	5.5	22.6	1.0	824
Burundi	bdi	17.5	4.7	19.2	0.9	217
Cambodia	khm	25.0	6.6	27.3	1.3	1578
Cameroon	cmr	24.4	6.5	26.7	1.2	1539
Canada	can	99.2	26.4	109	5.0	43350

Cape Verde	cpv	26.9*	7.1	29.4	1.4	3126
Cayman Islands	cym	148*	39.4	162	7.4	83898
Central African Republic	caf	22.8*	6.1	25	1.1	435
Chad	tcd	21.5	5.7	23.5	1.1	644
Chile	chl	55.7	14.8	61	2.8	13174
China	chn	18.1	4.8	19.8	0.9	10409
Colombia	col	33.5	8.9	36.6	1.7	5304
Comoros	com	24.5*	6.5	26.8	1.2	1520
Cook Islands	cok	47.1*	12.5	51.6	2.4	16642
Costa Rica	cri	43.5	11.6	47.6	2.2	12179
Croatia	hrv	44.5	11.8	48.8	2.2	14270
Cuba	cub	42.8	11.4	46.8	2.2	9500
Curacao	cuw	46.7*	12.4	51.1	2.3	16356
Cyprus	cyp	59.6	15.9	65.3	3.0	28281
Czechia	cze	63.3	16.8	69.3	3.2	22993
Denmark	dnk	113	30.2	124	5.7	60837
Djibouti	dji	26.6*	7.1	29.1	1.3	2922
Dominica	dma	32.7*	8.7	35.8	1.6	7003
Dominican Republic	dom	34.5	9.2	37.8	1.7	7168
Democratic Republic of the Congo	cod	23.0*	6.1	25.1	1.2	525
Ecuador	ecu	29.9	7.9	32.7	1.5	5645
Egypt	egy	22.7	6.0	24.9	1.1	3572
El Salvador	slv	30.7	8.2	33.6	1.5	3962
Equatorial Guinea	gnq	33.4	8.9	36.5	1.7	6199
Eritrea	eri	23.0*	6.1	25.2	1.2	566
Estonia	est	54.8	14.6	60	2.8	23595
Eswatini	swz	27.2*	7.2	29.8	1.4	3373
Ethiopia	eth	21.6	5.7	23.7	1.1	919
Falkland Islands	flk	128*	34.2	141	6.5	70800
Faroe Islands	fro	116*	30.7	127	5.8	62235
Fiji	fji	30.4	8.1	33.2	1.5	4816
Finland	fin	118	31.4	129	5.9	49170
France	fra	97.7	26.0	107	4.9	39180
French Guiana	guf	34.6*	9.2	37.9	1.7	8300
French Polynesia	pyf	51.0*	13.6	55.8	2.6	19186
Gabon	gab	31.2	8.3	34.2	1.6	6680
Gambia	gmb	20.2	5.4	22.1	1.0	704
Georgia	geo	25.7	6.8	28.2	1.3	4256
Germany	deu	109	28.9	119	5.5	46749
Ghana	gha	22.3	5.9	24.4	1.1	2177
Gibraltar	gib	115*	30.5	126	5.8	61700

Greece	grc	44.2	11.8	48.4	2.2	17617
Greenland	grl	104*	27.7	114	5.2	54693
Grenada	grd	34.8*	9.3	38.1	1.8	8438
Guadeloupe	glp	56.7*	15.1	62.1	2.8	22999
Guam	gum	74.3*	19.8	81.4	3.7	34781
Guatemala	gtm	36.2	9.6	39.7	1.8	4610
Guernsey	ggy	105*	27.8	114	5.3	54884
Guinea	gin	28.6	7.6	31.3	1.4	1074
Guinea-Bissau	gnb	23.2*	6.2	25.4	1.2	710
Guyana	guy	28.1	7.5	30.8	1.4	6863
Haiti	hti	23.4	6.2	25.6	1.2	1283
Holy See	vat	54.0*	14.4	59.1	2.7	21198
Honduras	hnd	25.3	6.7	27.7	1.3	2354
Hong Kong	hkg	123	32.6	134	6.2	46109
Hungary	hun	43.6	11.6	47.7	2.2	16126
Iceland	isl	135	35.9	148	6.8	58848
India	ind	8.6	2.3	9.5	0.4	1913
Indonesia	idn	11.0	2.9	12	0.6	3896
Iran	irn	41.4	11.0	45.3	2.1	2746
Iraq	irq	28.5	7.6	31.2	1.4	4251
Ireland	irl	113	30.0	124	5.7	85973
Isle of Man	imn	142*	37.6	155	7.1	79531
Israel	isr	105	28.0	115	5.3	44847
Italy	ita	86.8	23.1	95	4.4	31923
ivory-coast	civ	23.8	6.3	26	1.2	2349
Jamaica	jam	32.9	8.8	36.1	1.7	4897
Japan	jpn	115	30.5	126	5.8	39987
Jersey	jey	105*	28.0	115	5.3	55324
Jordan	jor	29.5	7.8	32.3	1.5	3999
Kazakhstan	kaz	36.6	9.7	40.1	1.8	9122
Kenya	ken	17.1	4.5	18.7	0.9	1936
Kiribati	kir	24.3*	6.5	26.6	1.2	1404
Kosovo	kos	28.2*	7.5	30.8	1.4	3990
Kuwait	kwt	59.1	15.7	64.7	3.0	24298
Kyrgyzstan	kgz	22.3	5.9	24.4	1.1	1257
Laos	lao	23.8	6.3	26	1.2	2593
Latvia	lva	52.1	13.8	57	2.6	18096
Lebanon	lbn	26.6	7.1	29.1	1.3	5600
Lesotho	lso	23.6	6.3	25.9	1.2	917
Liberia	lbr	23.1*	6.1	25.3	1.2	598
Libya	lby	32.7*	8.7	35.8	1.6	7035

Liechtenstein	lie	270*	71.9	296	14.0	165287
Lithuania	ltu	50.4	13.4	55.1	2.5	20382
Luxembourg	lux	198	52.5	216	9.9	116905
Macao	mac	78.4*	20.9	85.8	3.9	37475
Madagascar	mdg	21.1	5.6	23	1.1	462
Malawi	mwi	28.0	7.5	30.7	1.4	622
Malaysia	mys	36.4	9.7	39.8	1.8	10164
Maldives	mdv	33.0*	8.8	36.1	1.7	7217
Mali	mli	23.4*	6.2	25.6	1.2	823
Malta	mlt	57.7	15.4	63.2	2.9	29598
Marshall Islands	mhl	30.5*	8.1	33.4	1.5	5546
Martinique	mtq	62.2*	16.5	68.1	3.1	26666
Mauritania	mrt	24.9*	6.6	27.3	1.3	1836
Mauritius	mus	45.7	12.1	50	2.3	9011
Mayotte	myt	52.2*	13.9	57.1	2.6	20008
Mexico	mex	27.5	7.3	30.1	1.4	8895
Micronesia	fsm	27.6*	7.4	30.3	1.4	3639
Moldova	mda	44.2	11.8	48.4	2.2	4376
Monaco	mco	296*	78.7	324	15.0	182537
Mongolia	mng	23.3	6.2	25.5	1.2	4041
Montenegro	mne	40.9	10.9	44.8	2.1	7677
Montserrat	msr	44.8*	11.9	49	2.3	15067
Morocco	mar	27.1	7.2	29.6	1.4	3258
Mozambique	moz	24.2	6.4	26.5	1.2	457
Myanmar	mmr	23.4	6.2	25.6	1.2	1480
Namibia	nam	38.5	10.3	42.2	1.9	4252
Nauru	nru	37.4*	9.9	40.9	1.9	10125
Nepal	npl	24.2	6.4	26.5	1.2	1139
Netherlands	nld	111	29.4	121	5.6	52163
New Caledonia	ncl	74.5*	19.8	81.6	3.7	34878
New Zealand	nzl	95.6	25.4	105	4.8	41761
Nicaragua	nic	22.5	6.0	24.6	1.1	1877
Niger	ner	20.8	5.5	22.8	1.0	565
Nigeria	nga	11.0	2.9	12	0.6	2075
Niue	niu	50.9*	13.5	55.7	2.6	19121
Norfolk Island	nfk	113*	29.9	123	5.7	60209
North Korea	prk	24.7*	6.6	27.1	1.2	1700
Northern Mariana	mnp	48.1*	12.8	52.7	2.4	17303
North Macedonia	mkd	33.1	8.8	36.2	1.7	5965
Norway	nor	128	34.0	140	6.4	68340
Oman	omn	38.9	10.3	42.6	2.0	16708

Pakistan	pak	10.3	2.7	11.3	0.5	1322
Palau	plw	43.7*	11.6	47.8	2.2	14349
Palestinian territory	pse	27.0*	7.2	29.6	1.4	3234
Panama	pan	44.0	11.7	48.2	2.2	13293
Papua New Guinea	png	21.3	5.7	23.3	1.1	2446
Paraguay	pry	28.4	7.6	31.1	1.4	5353
Peru	per	35.5	9.4	38.9	1.8	6064
Philippines	phl	24.0	6.4	26.3	1.2	3224
Poland	pol	64.2	17.1	70.3	3.2	15817
Portugal	prt	91.6	24.4	100	4.6	22242
Puerto Rico	pri	79.8	21.2	87.4	4.0	31427
Qatar	qat	70.5	18.8	77.2	3.5	52316
Republic of The Congo	cog	23.4	6.2	25.6	1.2	2011
Reunion	reu	55.1*	14.7	60.3	2.8	21937
Romania	rou	40.7	10.8	44.6	2.0	13047
Russia	rus	35.9	9.6	39.3	1.8	10194
Rwanda	rwa	19.9	5.3	21.7	1.0	774
Saint Helena, Ascens	shn	33.9*	9.0	37.1	1.7	7800
Saint Barthelemy	blm	80.7*	21.5	88.3	4.1	38994
Saint Kitts and Nevi	kna	50.0*	13.3	54.8	2.5	18554
Saint Lucia	lca	35.6*	9.5	39	1.8	8952
Saint Martin	maf	54.2*	14.4	59.3	2.7	21344
Saint Pierre and Miq	spm	74.5*	19.8	81.6	3.7	34900
Saint Vincent	vct	34.6*	9.2	37.9	1.7	8306
Samoa	wsm	28.2*	7.5	30.9	1.4	4043
San Marino	smr	90.2*	24.0	98.7	4.5	45321
Sao Tome and Princip	stp	25.4*	6.8	27.8	1.3	2155
Saudi Arabia	sau	60.3	16.0	66	3.0	20398
Senegal	sen	24.1	6.4	26.4	1.2	1492
Serbia	srb	37.6	10.0	41.2	1.9	7734
Seychelles	syc	35.3	9.4	38.6	1.8	12020
Sierra Leone	sle	23.5	6.3	25.7	1.2	493
Singapore	sgp	84.5	22.5	92.5	4.2	61274
Sint Maarten	sxm	66.0*	17.6	72.3	3.3	29223
Slovakia	svk	51.4	13.7	56.3	2.6	19552
Slovenia	svn	61.2	16.3	67	3.1	25558
Solomon Islands	slb	25.5*	6.8	27.9	1.3	2222
Somalia	som	23.0*	6.1	25.2	1.2	557
South Sudan	ssd	23.8*	6.3	26	1.2	1071
South Africa	zaf	33.6	8.9	36.7	1.7	5753
South Korea	kor	52.3	13.9	57.3	2.6	31721

Spain	esp	81.5	21.7	89.3	4.1	26984
Sri Lanka	lka	25.7	6.8	28.2	1.3	3852
Sudan	sdn	13.8	3.7	15.1	0.7	608
Suriname	sur	21.5	5.7	23.6	1.1	4797
Svalbard and Jan Mayen	sjm	128*	34.0	140	6.4	68340
Sweden	swe	79.5	21.1	87	4.0	52838
Switzerland	che	137	36.5	150	6.9	85898
Syrian Arab Republic	syr	23.0*	6.1	25.2	1.2	537
Taiwan	twn	74.8	19.9	81.9	3.8	28571
Tajikistan	tjk	20.7	5.5	22.7	1.0	852
Tanzania	tza	23.2	6.2	25.4	1.2	1104
Thailand	tha	47.3	12.6	51.7	2.4	7002
Timor-Leste	tls	27.4	7.3	30	1.4	1664
Togo	tgo	21.7	5.8	23.8	1.1	887
Tokelau	tkl	31.2*	8.3	34.1	1.6	6004
Tonga	ton	29.1*	7.7	31.8	1.5	4606
Trinidad and Tobago	tto	42.7*	11.4	46.8	2.1	13706
Tunisia	tun	25.7	6.8	28.2	1.3	3498
Turkey	tur	31.3	8.3	34.2	1.6	8639
Turkmenistan	tkm	29.8	7.9	32.6	1.5	7330
Turks and Caicos	tca	53.5*	14.2	58.6	2.7	20882
Tuvalu	tuv	29.2*	7.8	32	1.5	4675
Uganda	uga	23.1	6.1	25.3	1.2	847
Ukraine	ukr	31.1	8.3	34.1	1.6	3752
United Arab Emirates	are	60.9	16.2	66.6	3.1	37629
United Kingdom	gbr	94.1	25.0	103	4.7	40217
United States	usa	123	32.8	135	6.2	63529
Uruguay	ury	60.3	16.0	66.1	3.0	15650
US Virgin Islands	vir	81.3*	21.6	89	4.1	39411
Uzbekistan	uzb	11.1	2.9	12.1	0.6	1759
Vanuatu	vut	26.6*	7.1	29.1	1.3	2918
Venezuela	ven	24.5*	6.5	26.9	1.2	1566
Vietnam	vnm	10.6	2.8	11.6	0.5	3586
Wallis and Futuna	wlf	27.9*	7.4	30.5	1.4	3800
Western Sahara	esh	25.9*	6.9	28.4	1.3	2500
Yemen	yem	23.0*	6.1	25.2	1.2	579
Zambia	zmb	17.0	4.5	18.6	0.9	957
Zimbabwe	zwe	12.5	3.3	13.7	0.6	1373

*Extrapolated from GDP

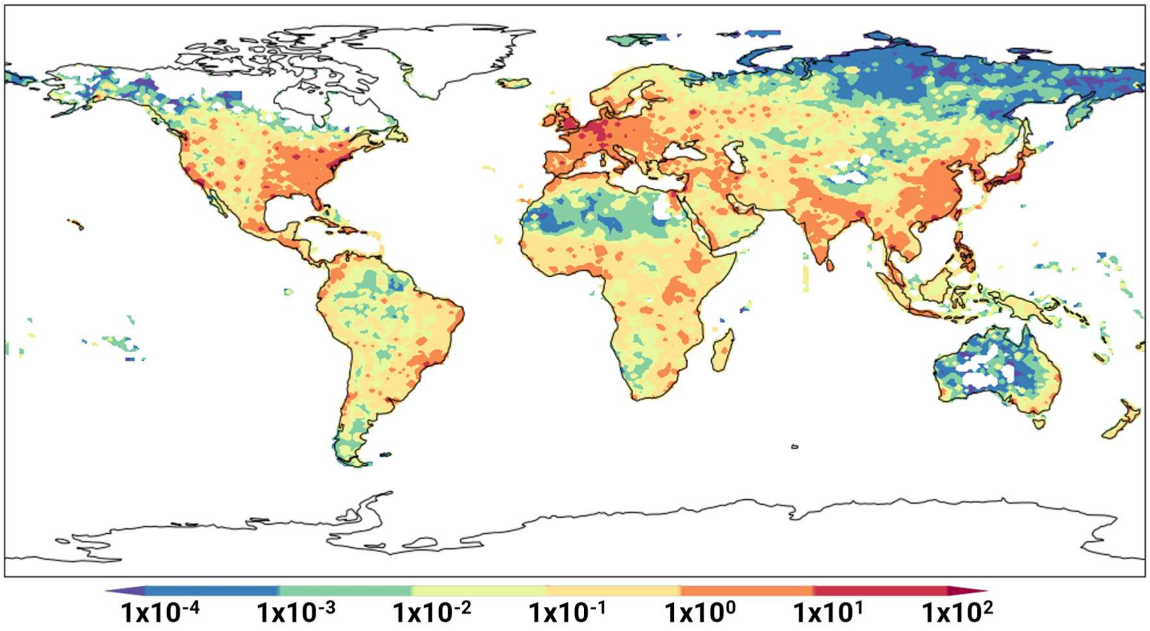

Figure S1: Map of predicted global emissions of D5 for 2020 in kg km^{-2} yr^{-1} at the model resolution (0.9x1.25°). Note the log scale. White areas have no emissions.

Halogen and Radical Chemistry

Due to this work's focus on the effect of chlorine on atmospheric VMS, it was important for the model to have an accurate representation of halogen chemistry. This was important not only because Cl concentrations directly impacted predicted VMS concentrations via oxidation, but also because halogens can indirectly affect predicted OH concentrations as discussed in the "Direct and Indirect Impacts of Short-Lived Halogens" section of the main text. In the base version of CESM2.2, there were no surface emissions of chlorine containing species. However, lower boundary conditions for 12 chlorine contain species (primarily CFCs and HCFCs) were included (Table S4). There were also 14 additional chlorine species which were formed or consumed by atmospheric reactions but did not have lower boundary conditions or surface emissions (Table S5). In total, the base model included 90 atmospheric reactions involving at least 1 chlorine containing species. 47 of these reactions resulted in the formation of chlorine radicals, 35 of which involved the decomposition of chlorine containing compounds by photolysis or reactions with singlet oxygen ($O(^1D)$). 19 of these species which photolyzed or reacted with $O(^1D)$ were CFCs or HCFCs. There were 19 reactions which consumed chlorine radicals, primarily consisting of the oxidation of non-chlorine containing compounds.

Table S4: Chlorine emissions and lower boundary conditions included in CESM2 and CESM2-SLH

Model Species Name	Global Yearly Average (2020) Lower Boundary Condition (Mole Fraction)	Global Yearly (2020) Surface Emissions (kg yr^{-1})	Model Inclusion
CCL4	$7.52X10^{-11}$	N/A	Both
CF2CLBR	$3.10X10^{-12}$	N/A	Both
CFC113	$6.83X10^{-11}$	N/A	Both
CH3CCL3	$1.08X10^{-12}$	N/A	Both
CH3CL	$5.63X10^{-10}$	N/A	Both
CFC114	$1.61X10^{-11}$	N/A	Both
HCFC22	$2.45X10^{-10}$	N/A	Both
CFC115	$8.66X10^{-12}$	N/A	Both
HCFC141B	$2.59X10^{-11}$	N/A	Both
HCFC142B	$2.20X10^{-11}$	N/A	Both
CF2CL2	$4.95X10^{-10}$	N/A	Both
CFCL3	$2.18X10^{-10}$	N/A	Both
CH2CL2	N/A	$9.88X10^{8}$	CESM2-SLH Only
C2CL4	N/A	$8.77X10^{7}$	CESM2-SLH Only
CH2BRCL	N/A	$6.89X10^{6}$	CESM2-SLH Only
CHBR2CL	N/A	$1.36X10^{7}$	CESM2-SLH Only
CHBRCL2	N/A	$1.60X10^{7}$	CESM2-SLH Only
CH2ICL	N/A	$1.53X10^{8}$	CESM2-SLH Only

Table S5: Chlorine containing species included in CESM2 and CESM2-SLH

Model Species Name	Formula	Present In CESM2	Present In CESM2-SLH
BRCL	BrCl	Yes	Yes
C2CL4	C2Cl4	No	Yes
C2H4CL2	C2H4Cl2	No	Yes
CCL4	CCl_4	Yes	Yes
CFC11	$CFCl_3$	Yes	Yes
CFC113	CCl_2FCClF_2	Yes	Yes
CFC114	$CClF_2CClF_2$	Yes	Yes
CFC115	$CClF_2CF_3$	Yes	Yes
CFC12	CF_2Cl_2	Yes	Yes
CH2BRCL	CH_2BrCl	No	Yes
CH2CL2	CH_2Cl_2	No	Yes
CH2ICL	CH_2ICl	No	Yes
CH3CCL3	CH_3CCl_3	Yes	Yes
CH3CL	CH_3Cl	Yes	Yes
CHBR2CL	$CHBr_2Cl$	No	Yes
CHBRCL2	$CHBrCl_2$	No	Yes
CHCL2O2	$CHCl_2O_2$	No	Yes
CHCL3	$CHCl_3$	No	Yes
CL	Cl	Yes	Yes
CL2	Cl_2	Yes	Yes
CL2O2	Cl_2O_2	Yes	Yes
CLNO2	$ClNO_2$	Yes	Yes
CLO	ClO	Yes	Yes
CLONO2	$ClONO_2$	Yes	Yes
CLY	ClY	Yes	Yes
COCL2	$COCl_2$	No	Yes
COFCL	COFCl	Yes	Yes
HCFC141B	CH_3CCl_2F	Yes	Yes
HCFC142B	CH_3CClF_2	Yes	Yes
HCFC22	CHF_2Cl	Yes	Yes
HCL	HCl	Yes	Yes
HOCL	HOCl	Yes	Yes
ICL	ICl	No	Yes
ncl_a1	NaCl	Yes	Yes
ncl_a2	NaCl	Yes	Yes
ncl_a3	NaCl	Yes	Yes
OCLO	OClO	Yes	Yes

Due to the underprediction of Cl in the base version of the model, the recently published "Short Lived Halogen" (SLH) version of CESM2 (CESM2-SLH) was used for this modeling effort.[11] In addition to the lower boundary conditions present in the base model, CESM2-SLH included surface emissions for 6 chlorine containing species (Table S4). For the year 2020, these emissions added an additional 9.4×10^8 kg of chlorine to the atmosphere. A total of 11 chlorine containing species, primarily halogenated hydrocarbons and oxides, were added in CESM2-SLH to the original 26 present in the base model (Table S5). CESM2-SLH also added 66 new reactions involving chlorine containing species for a total of 155. Twent-three of these new reactions formed chlorine radicals while 7 consumed chlorine radicals. 11 of these new radical forming reactions consisted of the photolysis of the added halogenated hydrocarbons and oxides. The remainder were oxidation reactions involving these new species and hydroxyl radicals. Likewise, these new species were oxidized by chlorine radicals resulting in the formation of HCl. These Cl oxidation reactions comprised most of the added Cl consuming reactions. The remainder of the 36 added reactions which did not directly form or consume chlorine radicals were primarily heterogeneous and ice-phase reactions including the decomposition of halogen nitrates and the interaction of hypohalous acids with halogen hydrides. Predicted tropospheric concentrations of OH and Cl from the 6 model configurations are shown below (Table S6&7). In addition, diagnostic plots showing the change in tropospheric Cl concentrations between the T:1,CL:1 and the T:1,CL:0.01 model run are shown (Figure S2).

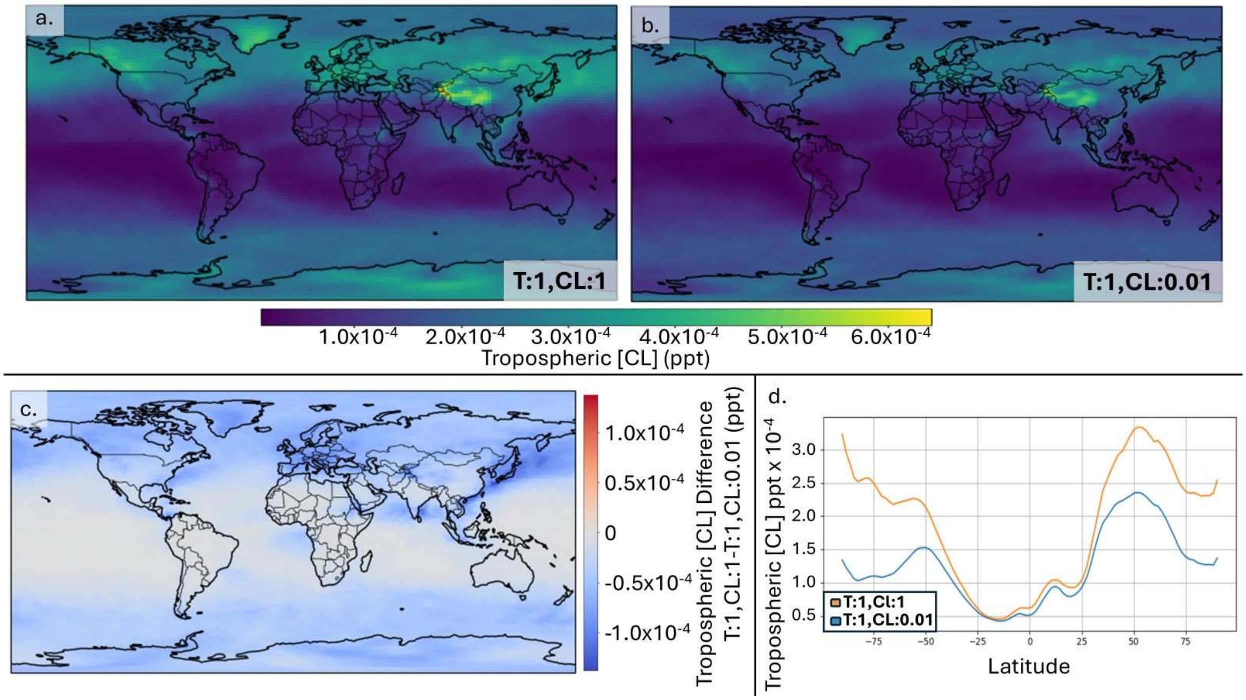

Figure S2: Diagnostic plots showing the predicted tropospheric concentration of Cl from the T:1,CL:1 and T:1,CL0.01 model runs. a. (Top Left) Map of predicted tropospheric Cl concentrations in ppt from the T:1,CL:1 model run in ppt. b. (Top Right) Map of predicted tropospheric Cl concentrations in ppt from the T:1,CL:0.01 model run in ppt. c. (Bottom Left) Map of the difference (ppt) in predicted tropospheric concentration of Cl between the T:1,Cl:1 model run and the T:1,CL:0.01 model run. d. (Bottom Right) Predicted zonal means (ppt) of the tropospheric concentration of Cl from both runs.

Table S6: Predicted tropospheric (>200 hPa) concentrations of OH (ppt) by region, season, and model.

Region	Season	T:0,CL:0	T:1,CL:0	T:0,CL:1	T:1,CL:1	T:1,CL:0.01	T:1,CL:1,SLH:0
Global	Full Year	0.077	0.078	0.078	0.077	0.078	0.086
Global	Dec-Feb	0.071	0.071	0.071	0.071	0.072	0.080
Global	Mar-May	0.073	0.074	0.073	0.073	0.074	0.082
Global	Jun-Aug	0.090	0.090	0.090	0.090	0.091	0.098
Global	Sep-Nov	0.075	0.075	0.075	0.075	0.076	0.083
NH Polar	Full Year	0.034	0.035	0.034	0.034	0.035	0.044
NH Polar	Dec-Feb	0.0014	0.0015	0.0014	0.0014	0.0015	0.0019
NH Polar	Mar-May	0.035	0.035	0.035	0.035	0.036	0.049
NH Polar	Jun-Aug	0.088	0.089	0.088	0.088	0.090	0.11
NH Polar	Sep-Nov	0.013	0.013	0.013	0.013	0.013	0.016
NH Mid-Latitudes	Full Year	0.081	0.081	0.081	0.081	0.082	0.092
NH Mid-Latitudes	Dec-Feb	0.025	0.026	0.025	0.025	0.026	0.031
NH Mid-Latitudes	Mar-May	0.079	0.080	0.079	0.079	0.081	0.095
NH Mid-Latitudes	Jun-Aug	0.15	0.15	0.15	0.15	0.16	0.17
NH Mid-Latitudes	Sep-Nov	0.067	0.067	0.067	0.067	0.068	0.073
Tropics	Full Year	0.10	0.10	0.10	0.10	0.10	0.11
Tropics	Dec-Feb	0.095	0.095	0.095	0.095	0.096	0.10
Tropics	Mar-May	0.099	0.099	0.099	0.099	0.10	0.11
Tropics	Jun-Aug	0.11	0.11	0.11	0.11	0.11	0.11
Tropics	Sep-Nov	0.10	0.10	0.10	0.10	0.10	0.11
SH Mid-Latitudes	Full Year	0.045	0.045	0.046	0.045	0.046	0.055
SH Mid-Latitudes	Dec-Feb	0.081	0.082	0.082	0.082	0.083	0.099
SH Mid-Latitudes	Mar-May	0.034	0.034	0.034	0.034	0.035	0.040
SH Mid-Latitudes	Jun-Aug	0.016	0.016	0.016	0.016	0.017	0.019
SH Mid-Latitudes	Sep-Nov	0.049	0.049	0.049	0.049	0.050	0.061
SH Polar	Full Year	0.023	0.023	0.023	0.023	0.023	0.032
SH Polar	Dec-Feb	0.055	0.056	0.056	0.055	0.056	0.074
SH Polar	Mar-May	0.0076	0.0077	0.0077	0.0077	0.0078	0.0094
SH Polar	Jun-Aug	0.0010	0.0010	0.0010	0.0010	0.0011	0.0014
SH Polar	Sep-Nov	0.027	0.027	0.027	0.027	0.027	0.042

Table S7: Predicted tropospheric concentrations of Cl (ppt) by region, season, and model.

Region	Season	T:0,CL:0	T:1,CL:0	T:0,CL:1	T:1,CL:1	T:1,CL:0.01	T:1,CL:1,SLH:0
Global	Full Year	$1.6x10^{-4}$	$1.5x10^{-4}$	$1.6x10^{-4}$	$1.6x10^{-4}$	$1.3x10^{-4}$	$2.4x10^{-5}$
Global	Dec-Feb	$1.6x10^{-4}$	$1.6x10^{-4}$	$1.6x10^{-4}$	$1.6x10^{-4}$	$1.3x10^{-4}$	$2.5x10^{-5}$
Global	Mar-May	$1.7x10^{-4}$	$1.7x10^{-4}$	$1.7x10^{-4}$	$1.7x10^{-4}$	$1.4x10^{-4}$	$2.6x10^{-5}$
Global	Jun-Aug	$1.5x10^{-4}$	$1.5x10^{-4}$	$1.5x10^{-4}$	$1.5x10^{-4}$	$1.3x10^{-4}$	$2.5x10^{-5}$
Global	Sep-Nov	$1.4x10^{-4}$	$1.4x10^{-4}$	$1.4x10^{-4}$	$1.4x10^{-4}$	$1.2x10^{-4}$	$2.1x10^{-5}$
NH Polar	Full Year	$2.7x10^{-4}$	$2.7x10^{-4}$	$2.7x10^{-4}$	$2.8x10^{-4}$	$2.2x10^{-4}$	$4.5x10^{-5}$
NH Polar	Dec-Feb	$1.2x10^{-4}$	$1.2x10^{-4}$	$1.2x10^{-4}$	$1.2x10^{-4}$	$8.8x10^{-5}$	$1.7x10^{-5}$
NH Polar	Mar-May	$4.5x10^{-4}$	$4.5x10^{-4}$	$4.5x10^{-4}$	$4.6x10^{-4}$	$3.4x10^{-4}$	$8.9x10^{-5}$
NH Polar	Jun-Aug	$4.1x10^{-4}$	$4.0x10^{-4}$	$4.1x10^{-4}$	$4.2x10^{-4}$	$3.8x10^{-4}$	$6.4x10^{-5}$
NH Polar	Sep-Nov	$1.1x10^{-4}$	$1.1x10^{-4}$	$1.1x10^{-4}$	$1.1x10^{-4}$	$9.0x10^{-5}$	$9.6x10^{-6}$
NH Mid-Latitudes	Full Year	$2.8x10^{-4}$	$2.8x10^{-4}$	$2.8x10^{-4}$	$2.9x10^{-4}$	$2.4x10^{-4}$	$3.6x10^{-5}$
NH Mid-Latitudes	Dec-Feb	$2.6x10^{-4}$	$2.6x10^{-4}$	$2.6x10^{-4}$	$2.5x10^{-4}$	$2.0x10^{-4}$	$2.9x10^{-5}$
NH Mid-Latitudes	Mar-May	$4.0x10^{-4}$	$4.0x10^{-4}$	$4.0x10^{-4}$	$4.1x10^{-4}$	$3.3x10^{-4}$	$5.2x10^{-5}$
NH Mid-Latitudes	Jun-Aug	$3.2x10^{-4}$	$3.2x10^{-4}$	$3.2x10^{-4}$	$3.3x10^{-4}$	$2.9x10^{-4}$	$4.6x10^{-5}$
NH Mid-Latitudes	Sep-Nov	$1.5x10^{-4}$	$1.5x10^{-4}$	$1.5x10^{-4}$	$1.5x10^{-4}$	$1.3x10^{-4}$	$1.6x10^{-5}$
Tropics	Full Year	$7.7x10^{-5}$	$7.7x10^{-5}$	$7.7x10^{-5}$	$7.7x10^{-5}$	$6.7x10^{-5}$	$1.5x10^{-5}$
Tropics	Dec-Feb	$7.2x10^{-5}$	$7.3x10^{-5}$	$7.3x10^{-5}$	$7.3x10^{-5}$	$6.5x10^{-5}$	$1.4x10^{-5}$
Tropics	Mar-May	$7.6x10^{-5}$	$7.6x10^{-5}$	$7.6x10^{-5}$	$7.7x10^{-5}$	$6.7x10^{-5}$	$1.5x10^{-5}$
Tropics	Jun-Aug	$8.6x10^{-5}$	$8.5x10^{-5}$	$8.6x10^{-5}$	$8.6x10^{-5}$	$7.2x10^{-5}$	$1.7x10^{-5}$
Tropics	Sep-Nov	$7.4x10^{-5}$	$7.4x10^{-5}$	$7.4x10^{-5}$	$7.4x10^{-5}$	$6.2x10^{-5}$	$1.5x10^{-5}$
SH Mid-Latitudes	Full Year	$1.7x10^{-4}$	$1.7x10^{-4}$	$1.7x10^{-4}$	$1.7x10^{-4}$	$1.4x10^{-4}$	$2.3x10^{-5}$
SH Mid-Latitudes	Dec-Feb	$2.4x10^{-4}$	$2.2x10^{-4}$	$2.3x10^{-4}$	$2.3x10^{-4}$	$2.0x10^{-4}$	$3.5x10^{-5}$
SH Mid-Latitudes	Mar-May	$1.0x10^{-4}$	$1.0x10^{-4}$	$1.0x10^{-4}$	$1.0x10^{-4}$	$9.4x10^{-5}$	$1.0x10^{-5}$
SH Mid-Latitudes	Jun-Aug	$1.0x10^{-4}$	$1.0x10^{-4}$	$1.0x10^{-4}$	$1.0x10^{-4}$	$8.4x10^{-5}$	$1.6x10^{-5}$
SH Mid-Latitudes	Sep-Nov	$2.4x10^{-4}$	$2.4x10^{-4}$	$2.4x10^{-4}$	$2.4x10^{-4}$	$1.8x10^{-4}$	$3.0x10^{-5}$
SH Polar	Full Year	$2.4x10^{-4}$	$2.4x10^{-4}$	$2.5x10^{-4}$	$2.4x10^{-4}$	$2.0x10^{-4}$	$4.2x10^{-5}$
SH Polar	Dec-Feb	$4.2x10^{-4}$	$4.0x10^{-4}$	$4.2x10^{-4}$	$3.9x10^{-4}$	$3.4x10^{-4}$	$8.0x10^{-5}$
SH Polar	Mar-May	$8.5x10^{-5}$	$8.4x10^{-5}$	$8.3x10^{-5}$	$8.7x10^{-5}$	$7.8x10^{-5}$	$1.1x10^{-5}$
SH Polar	Jun-Aug	$4.6x10^{-5}$	$4.7x10^{-5}$	$4.6x10^{-5}$	$4.7x10^{-5}$	$3.6x10^{-5}$	$9.6x10^{-6}$
SH Polar	Sep-Nov	$4.3x10^{-4}$	$4.3x10^{-4}$	$4.3x10^{-4}$	$4.3x10^{-4}$	$3.4x10^{-4}$	$6.6x10^{-5}$

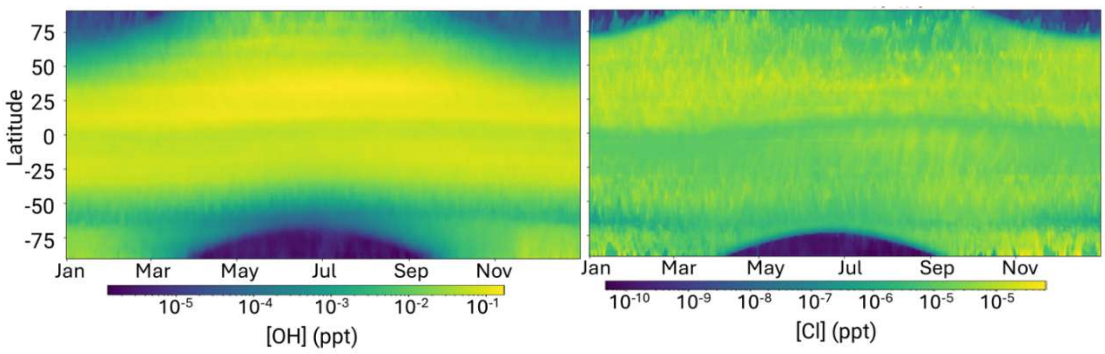

Figure S3: Time series of predicted zonal mean near-surface concentrations of OH (left) and Cl (right) in ppt.

VMS Chemistry

The NetCDF files containing all of the model outputs have been released to an open access data repository.[12] However, for convenience we have included here tables and figures of the predicted near-surface concentrations and lifetimes of VMS species by season, region, and model configuration.

Table S8: Predicted D4 near-surface concentrations (ng m^{-3}) by region, season, and model.

Region	Season	T:0,CL:0	T:1,CL:0	T:0,CL:1	T:1,CL:1	T:1,CL:0.01	T:1,CL:1,SLH:0
Global	Full Year	0.44	0.47	0.36	0.38	0.40	0.45
Global	Dec-Feb	0.67	0.71	0.54	0.57	0.59	0.70
Global	Mar-May	0.52	0.59	0.38	0.40	0.43	0.55
Global	Jun-Aug	0.26	0.27	0.24	0.25	0.26	0.26
Global	Sep-Nov	0.30	0.32	0.29	0.30	0.30	0.31
NH Polar	Full Year	0.68	0.78	0.38	0.43	0.47	0.72
NH Polar	Dec-Feb	1.3	1.3	0.82	0.91	0.97	1.3
NH Polar	Mar-May	1.2	1.4	0.48	0.54	0.65	1.3
NH Polar	Jun-Aug	0.078	0.12	0.053	0.065	0.071	0.085
NH Polar	Sep-Nov	0.20	0.23	0.17	0.20	0.20	0.22
NH Mid-Latitudes	Full Year	1.4	1.5	1.1	1.2	1.2	1.4
NH Mid-Latitudes	Dec-Feb	2.1	2.2	1.8	1.8	1.9	2.2
NH Mid-Latitudes	Mar-May	1.6	1.8	1.2	1.3	1.3	1.7
NH Mid-Latitudes	Jun-Aug	0.75	0.79	0.72	0.74	0.75	0.76
NH Mid-Latitudes	Sep-Nov	0.95	0.98	0.92	0.94	0.95	0.97
Tropics	Full Year	0.26	0.28	0.24	0.25	0.25	0.27
Tropics	Dec-Feb	0.37	0.40	0.32	0.34	0.35	0.39
Tropics	Mar-May	0.29	0.32	0.25	0.26	0.27	0.29
Tropics	Jun-Aug	0.19	0.20	0.19	0.19	0.19	0.19
Tropics	Sep-Nov	0.20	0.21	0.19	0.20	0.20	0.21
SH Mid-Latitudes	Full Year	0.056	0.065	0.049	0.054	0.055	0.059
SH Mid-Latitudes	Dec-Feb	0.029	0.034	0.027	0.028	0.029	0.029
SH Mid-Latitudes	Mar-May	0.037	0.040	0.035	0.037	0.037	0.037
SH Mid-Latitudes	Jun-Aug	0.086	0.096	0.078	0.086	0.086	0.093
SH Mid-Latitudes	Sep-Nov	0.072	0.088	0.057	0.064	0.068	0.078
SH Polar	Full Year	0.023	0.032	0.015	0.019	0.021	0.026
SH Polar	Dec-Feb	0.0046	0.010	0.0016	0.0027	0.0033	0.0038
SH Polar	Mar-May	0.0053	0.0079	0.0037	0.0050	0.0052	0.0056
SH Polar	Jun-Aug	0.037	0.046	0.030	0.036	0.037	0.042
SH Polar	Sep-Nov	0.045	0.062	0.026	0.033	0.037	0.052

Table S9: Predicted D5 near-surface concentrations (ng m^{-3}) by region, season, and model.

Region	Season	T:0,CL:0	T:1,CL:0	T:0,CL:1	T:1,CL:1	T:1,CL:0.01	T:1,CL:1,SLH:0
Global	Full Year	1.9	2.0	1.6	1.6	1.7	1.9
Global	Dec-Feb	2.9	3.1	2.3	2.4	2.5	3.1
Global	Mar-May	2.1	2.4	1.6	1.7	1.7	2.2
Global	Jun-Aug	1.1	1.1	1.1	1.1	1.1	1.1
Global	Sep-Nov	1.3	1.4	1.3	1.3	1.3	1.3
NH Polar	Full Year	2.7	3.1	1.4	1.6	1.7	2.9
NH Polar	Dec-Feb	5.5	5.9	3.2	3.6	3.8	5.8
NH Polar	Mar-May	4.3	5.4	1.5	1.8	2.1	4.8
NH Polar	Jun-Aug	0.21	0.29	0.17	0.19	0.20	0.22
NH Polar	Sep-Nov	0.71	0.84	0.6	0.68	0.71	0.80
NH Mid-Latitudes	Full Year	6.0	6.4	5.1	5.3	5.4	6.2
NH Mid-Latitudes	Dec-Feb	9.7	10	7.9	8.2	8.5	10
NH Mid-Latitudes	Mar-May	6.7	7.5	5.1	5.4	5.7	7.1
NH Mid-Latitudes	Jun-Aug	3.3	3.4	3.2	3.3	3.3	3.3
NH Mid-Latitudes	Sep-Nov	4.2	4.4	4.1	4.2	4.2	4.3
Tropics	Full Year	1.1	1.1	1.0	1.0	1.0	1.1
Tropics	Dec-Feb	1.5	1.6	1.3	1.4	1.4	1.6
Tropics	Mar-May	1.1	1.2	1.0	1.0	1.1	1.1
Tropics	Jun-Aug	0.83	0.85	0.81	0.83	0.83	0.84
Tropics	Sep-Nov	0.87	0.89	0.85	0.86	0.87	0.88
SH Mid-Latitudes	Full Year	0.22	0.24	0.20	0.21	0.21	0.23
SH Mid-Latitudes	Dec-Feb	0.12	0.13	0.12	0.12	0.12	0.12
SH Mid-Latitudes	Mar-May	0.15	0.16	0.15	0.15	0.16	0.16
SH Mid-Latitudes	Jun-Aug	0.34	0.38	0.31	0.34	0.34	0.37
SH Mid-Latitudes	Sep-Nov	0.26	0.31	0.21	0.23	0.24	0.28
SH Polar	Full Year	0.067	0.091	0.042	0.053	0.057	0.076
SH Polar	Dec-Feb	0.0068	0.017	0.0027	0.0043	0.005	0.0049
SH Polar	Mar-May	0.013	0.019	0.009	0.012	0.012	0.014
SH Polar	Jun-Aug	0.12	0.15	0.092	0.11	0.11	0.14
SH Polar	Sep-Nov	0.13	0.18	0.065	0.084	0.097	0.15

Table S10: Predicted D6 near-surface concentrations (ng m^{-3}) by region, season, and model.

Region	Season	T:0,CL:0	T:1,CL:0	T:0,CL:1	T:1,CL:1	T:1,CL:0.01	T:1,CL:1,SLH:0
Global	Full Year	0.089	0.095	0.075	0.077	0.08	0.092
Global	Dec-Feb	0.14	0.15	0.11	0.12	0.12	0.15
Global	Mar-May	0.097	0.11	0.075	0.078	0.081	0.10
Global	Jun-Aug	0.054	0.055	0.052	0.053	0.053	0.054
Global	Sep-Nov	0.064	0.066	0.061	0.062	0.063	0.065
NH Polar	Full Year	0.12	0.14	0.056	0.064	0.071	0.13
NH Polar	Dec-Feb	0.26	0.28	0.14	0.15	0.17	0.28
NH Polar	Mar-May	0.18	0.23	0.059	0.068	0.081	0.21
NH Polar	Jun-Aug	0.0078	0.010	0.0068	0.0076	0.0079	0.0083
NH Polar	Sep-Nov	0.029	0.035	0.024	0.028	0.029	0.033
NH Mid-Latitudes	Full Year	0.29	0.31	0.25	0.26	0.26	0.30
NH Mid-Latitudes	Dec-Feb	0.48	0.51	0.39	0.4	0.42	0.51
NH Mid-Latitudes	Mar-May	0.31	0.35	0.25	0.26	0.27	0.33
NH Mid-Latitudes	Jun-Aug	0.16	0.16	0.16	0.16	0.16	0.16
NH Mid-Latitudes	Sep-Nov	0.21	0.21	0.20	0.21	0.21	0.21
Tropics	Full Year	0.051	0.053	0.048	0.049	0.049	0.052
Tropics	Dec-Feb	0.068	0.072	0.061	0.063	0.065	0.071
Tropics	Mar-May	0.052	0.055	0.048	0.049	0.05	0.053
Tropics	Jun-Aug	0.041	0.041	0.040	0.040	0.041	0.041
Tropics	Sep-Nov	0.042	0.043	0.041	0.042	0.042	0.042
SH Mid-Latitudes	Full Year	0.0099	0.011	0.0091	0.0096	0.0097	0.01
SH Mid-Latitudes	Dec-Feb	0.0057	0.006	0.0056	0.0057	0.0057	0.0057
SH Mid-Latitudes	Mar-May	0.0074	0.0076	0.0072	0.0074	0.0074	0.0075
SH Mid-Latitudes	Jun-Aug	0.016	0.017	0.014	0.015	0.015	0.017
SH Mid-Latitudes	Sep-Nov	0.011	0.013	0.0093	0.010	0.010	0.012
SH Polar	Full Year	0.0023	0.0032	0.0014	0.0017	0.0019	0.0027
SH Polar	Dec-Feb	0.00015	0.00037	0.000069	0.0001	0.00011	$9.70x10^{-5}$
SH Polar	Mar-May	0.00041	0.00059	0.00028	0.00036	0.00037	0.00043
SH Polar	Jun-Aug	0.0045	0.0057	0.0033	0.004	0.0041	0.0053
SH Polar	Sep-Nov	0.0041	0.0061	0.0019	0.0025	0.0029	0.0049

Table S11: Predicted VMS lifetimes (days) by season and model

Model	Period	D4	D5	D6
T:0,CL:0	Full Year	8.0	5.2	3.8
T:0,CL:0	Mar-May	6.7	4.0	2.7
T:0,CL:0	June-Aug	3.8	2.2	1.5
T:0,CL:0	Sep-Nov	7.2	4.5	3.2
T:0,CL:0	Dec-Feb	14.5	10.1	7.8
T:1,CL:0	Full Year	10.2	6.6	4.8
T:1,CL:0	Mar-May	9.0	5.4	3.7
T:1,CL:0	June-Aug	5.3	3.0	2.0
T:1,CL:0	Sep-Nov	9.4	5.8	4.1
T:1,CL:0	Dec-Feb	17.6	12.4	9.6
T:0,CL:1	Full Year	5.4	3.3	2.3
T:0,CL:1	Mar-May	4.4	2.5	1.7
T:0,CL:1	June-Aug	3.3	1.9	1.3
T:0,CL:1	Sep-Nov	5.7	3.5	2.5
T:0,CL:1	Dec-Feb	8.3	5.3	3.7
T:1,CL:1	Full Year	6.7	4.0	2.7
T:1,CL:1	Mar-May	5.5	3.1	2.1
T:1,CL:1	June-Aug	4.3	2.4	1.6
T:1,CL:1	Sep-Nov	7.1	4.3	2.9
T:1,CL:1	Dec-Feb	9.8	6.2	4.4
T:1,CL:1, SLH:0	Full Year	9.2	5.9	4.4
T:1,CL:1, SLH:0	Mar-May	7.7	4.6	3.2
T:1,CL:1, SLH:0	June-Aug	4.8	2.8	1.8
T:1,CL:1, SLH:0	Sep-Nov	8.6	5.4	3.9
T:1,CL:1, SLH:0	Dec-Feb	15.8	11.1	8.6
T:1,CL:0.01	Full Year	7.1	4.3	3.0
T:1,CL:0.01	Mar-May	6.0	3.4	2.3
T:1,CL:0.01	June-Aug	4.4	2.5	1.7
T:1,CL:0.01	Sep-Nov	7.5	4.5	3.1
T:1,CL:0.01	Dec-Feb	10.8	6.8	4.9

Table S12: Gross predicted D5 deposition velocity, flux, and integrated deposition (the total mass deposited in the specified region during the listed time period), by latitude and season from the T:1,CL:1 model. Note that due to the model constraints described in the *Methods* section of the main paper these values cannot be interpreted as net deposition.

		Dec-Feb	Mar-May	Jun-Aug	Sep-Nov	Annual
Arctic (66.5° - 90°)	Velocity (cm s^{-1})	$1.2X10^{-3}$	$4.6X10^{-3}$	$3.1X10^{-2}$	$9.1X10^{-3}$	$1.5X10^{-2}$
	Flux (μg m^{-2} yr^{-1})	1.6	0.51	0.87	2.6	1.3
	Integrated Deposition (kg)	$2.6X10^{3}$	$4.4X10^{3}$	$1.3X10^{4f}$	$8.2X10^{3}$	$2.9X10^{4}$
Mid-Latitudes (NH) (23.5° - 66.5°)	Velocity (cm s^{-1})	$1.3X10^{-2}$	$2.6X10^{-2}$	$4.5X10^{-2}$	$2.4X10^{-2}$	$3.2X10^{-2}$
	Flux (μg m^{-2} yr^{-1})	66	65	69	73	69
	Integrated Deposition (kg)	$2.2X10^{6}$	$2.3X10^{6}$	$2.4X10^{6}$	$2.2X10^{6}$	$9.1X10^{6}$
Northern Tropics (0° - 23.5°)	Velocity (cm s^{-1})	$2.2X10^{-2}$	$2.4X10^{-2}$	$2.8X10^{-2}$	$2.7X10^{-2}$	$2.6X10^{-2}$
	Flux (μg m^{-2} yr^{-1})	25	20	21	25	22
	Integrated Deposition (kg)	$5.1X10^{5}$	$5.4X10^{5}$	$6.4X10^{5}$	$6.4X10^{5}$	$2.3X10^{6}$
Southern Tropics (-23.5° - 0°)	Velocity (cm s^{-1})	$3.1X10^{-2}$	$2.9X10^{-2}$	$2.4X10^{-2}$	$2.6X10^{-2}$	$2.6X10^{-2}$
	Flux (μg m^{-2} yr^{-1})	12	13	11	9.4	11
	Integrated Deposition (kg)	$3.3X10^{5}$	$2.8X10^{5}$	$2.4X10^{5}$	$3.1X10^{5}$	$1.2X10^{6}$
Mid-Latitudes (SH) (-66.5° to 23.5°)	Velocity (cm s^{-1})	$1.1X10^{-2}$	$9.7X10^{-3}$	$9.4X10^{-3}$	$1.1X10^{-2}$	$1.0X10^{-2}$
	Flux (μg m^{-2} yr^{-1})	3.0	3.5	4.3	3.3	3.7
	Integrated Deposition (kg)	$1.2X10^{5}$	$1.4X10^{5}$	$1.1X10^{5}$	$1.0X10^{5}$	$4.7X10^{5}$
Antarctic (-90°- -66.5°)	Velocity (cm s^{-1})	$5.2X10^{-3}$	$1.7X10^{-3}$	$8.2X10^{-4}$	$1.9X10^{-3}$	$1.5X10^{-3}$
	Flux (μg m^{-2} yr^{-1})	0.0062	0.013	0.037	0.012	0.021
	Integrated Deposition (kg)	$6.6X10^{1}$	$1.9X10^{2}$	$6.1X10^{1}$	$3.2X10^{1}$	$3.5X10^{2}$

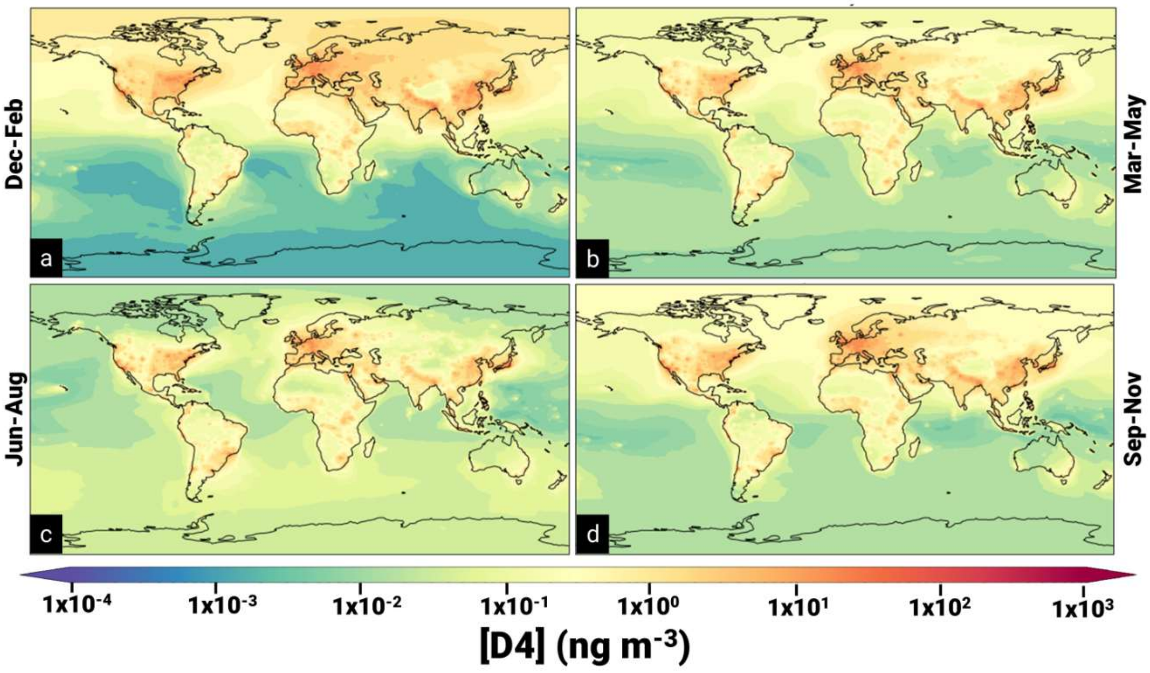

Figure S4: Predicted average seasonal near-surface concentrations of D4 from the "T:1,CL:1" model in ng m-3.

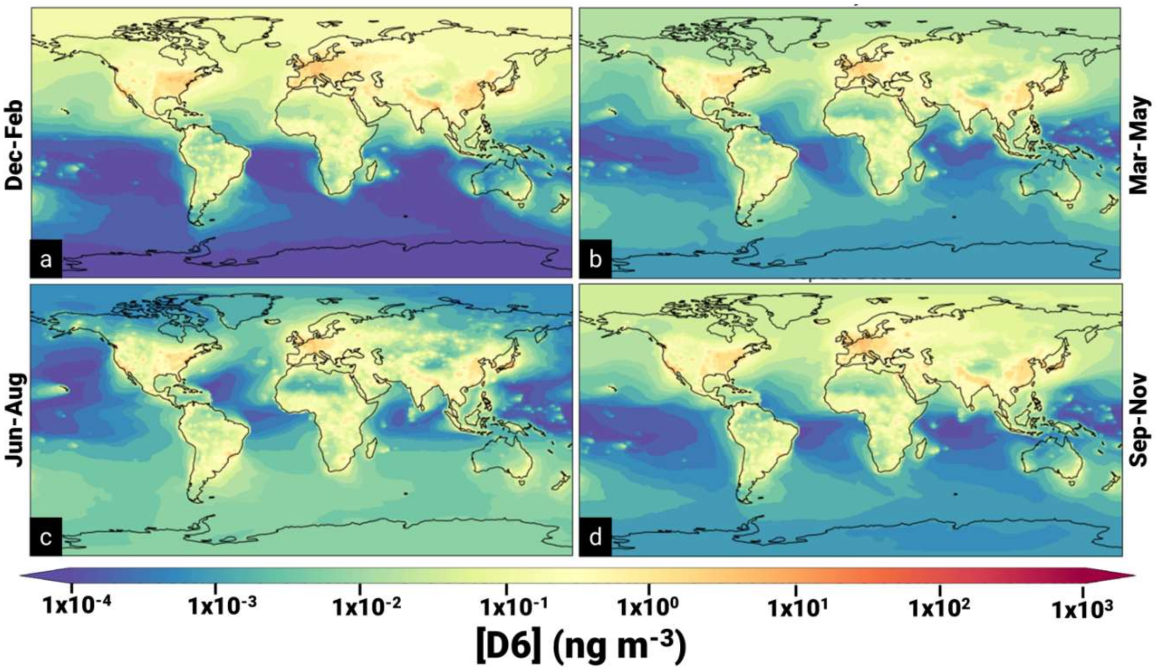

Figure S5: Predicted average seasonal near-surface concentrations of D6 from the "T:1,CL:1" model in ng m-3.

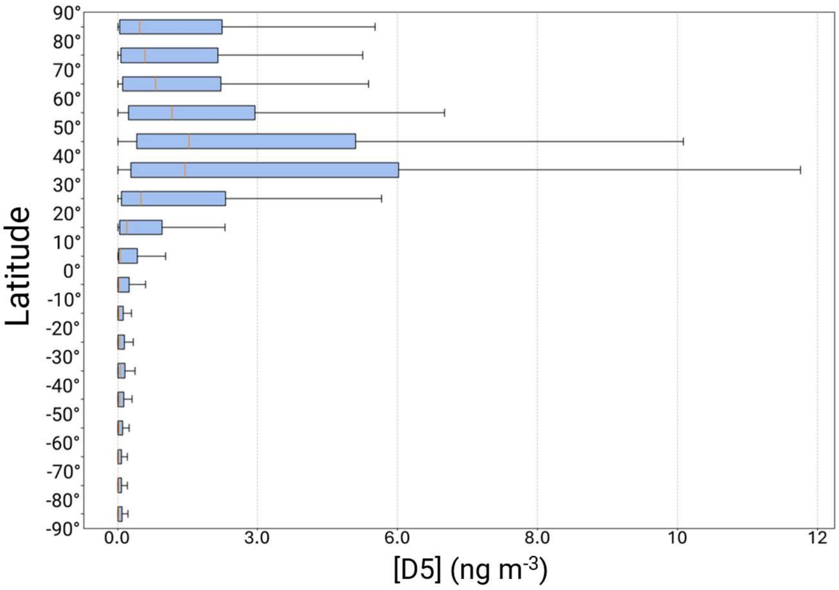

Figure S6: Predicted average near-surface concentrations of D5 (ng m-3) binned by latitude. Y axis labels represent the start and end of each latitude bin. Box and whisker plots represent the range of average daily near-surface concentrations for the latitude band of interest for all of 2020. The solid orange line represents the median, the shaded blue box represents the 1st and 3rd quartile, and the whiskers represent the 2.5th and 97.5th percentile of the data.

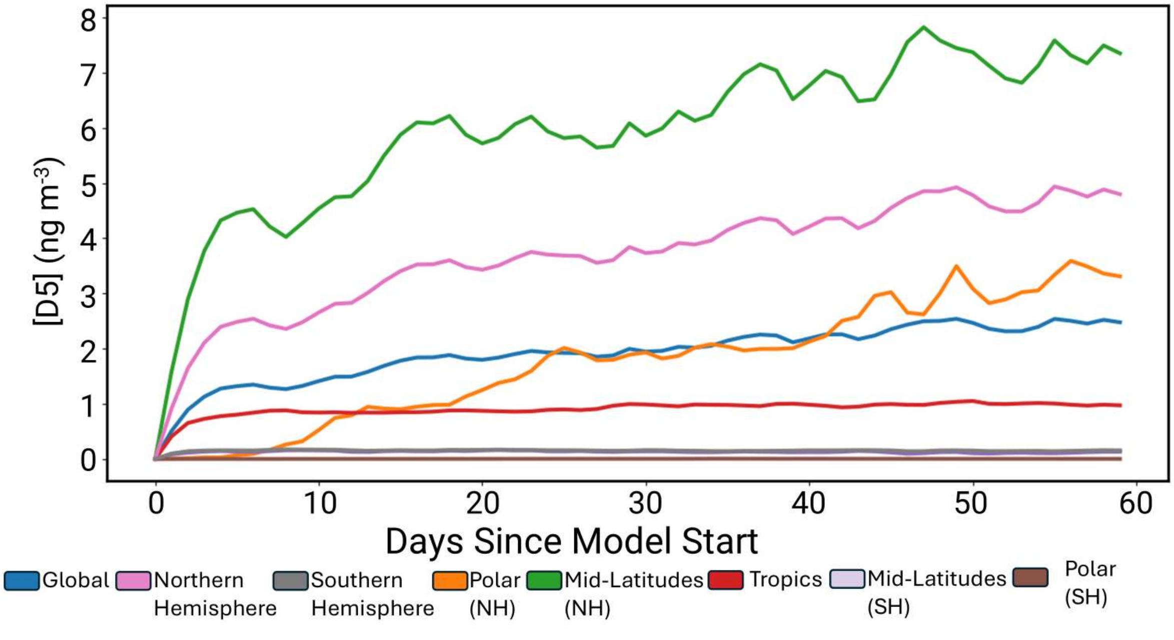

Figure S7: Timeseries of predicted regional D5 near-surface concentrations from the "T:1,CL:1" model during the 60-day model spin-up period.

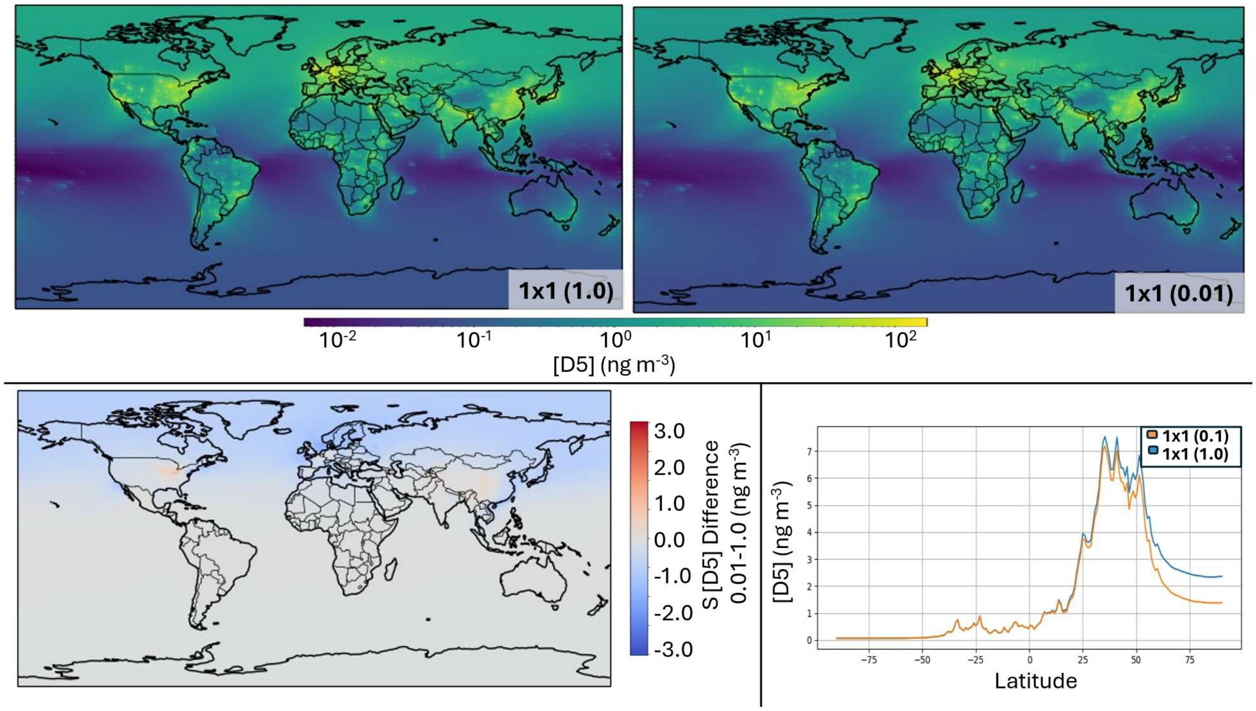

Figure S8: Diagnostic plots showing the predicted near-surface concentration of D5 from the T:1,CL:1 and T:1,CL0.01 model runs. A. (Top Left) Map of near-surface D5 concentrations in ppt from the T:1,CL:1 model run in ng m-3. B. (Top Right) Map of surface predicted D5 concentrations in ng m^{-3} from the T:1,CL:0.01 model run in ppt. C. (Bottom Left) Map of the difference (ng m-3) in the predicted near-surface concentration of D5 between the T:1,Cl:1 model run and the T:1,CL:0.01 model run. D. (Bottom right) Zonal means (ng m-3) of the predicted near-surface concentration of D5 from both runs.

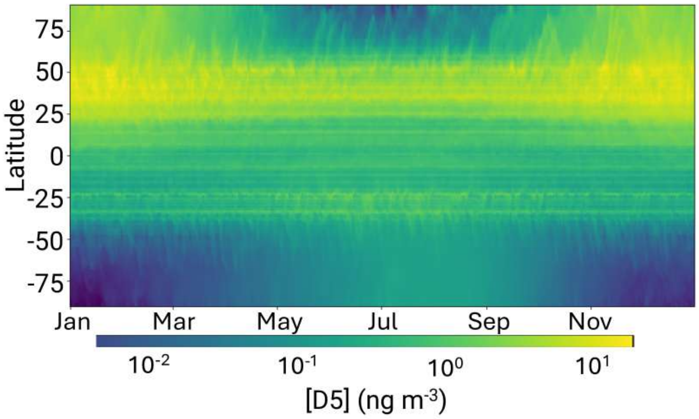

Figure S9: Time series of predicted zonal mean near-surface concentrations of D5 in ng m^{-3}.

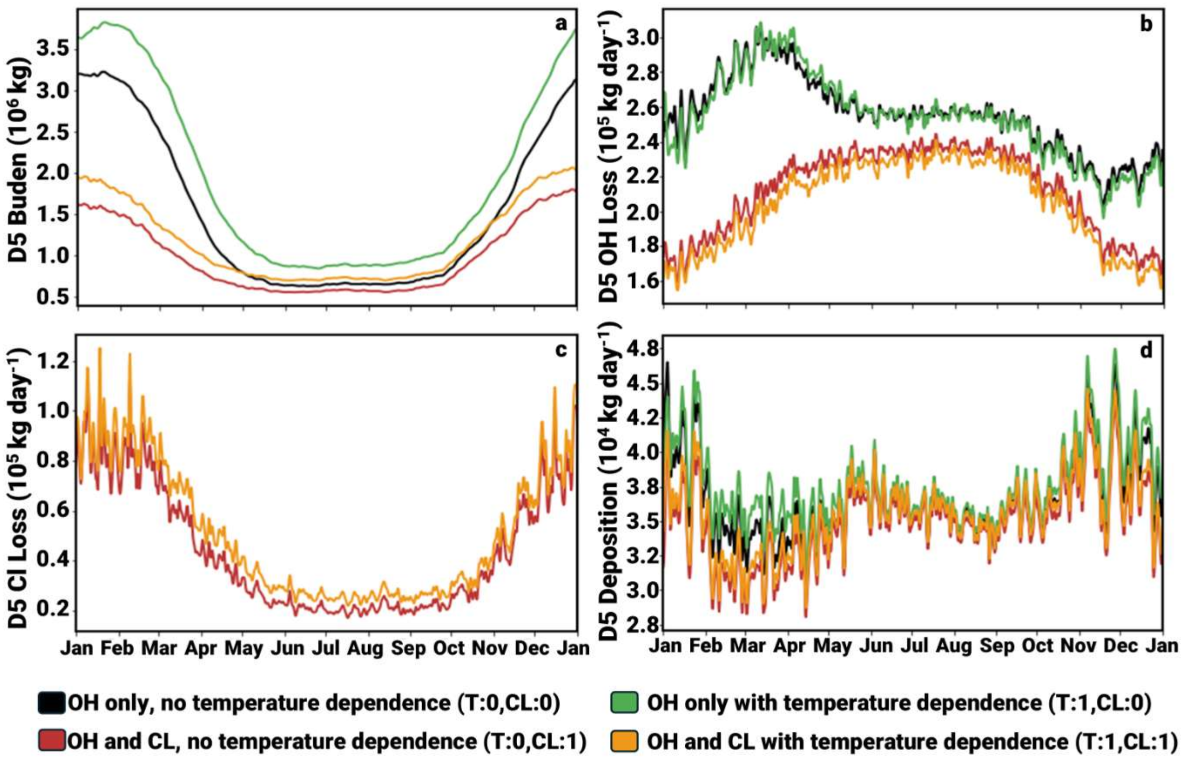

Figure S10: Time series of daily a: (top left) predicted total atmospheric burden of D5, b: (top right) total global loss of D5 to OH, c: (bottom left) total global loss of D5 to Cl, and d: (bottom right) total global loss of D5 to deposition from the four core model configurations. Tick marks indicate the first day of each month.

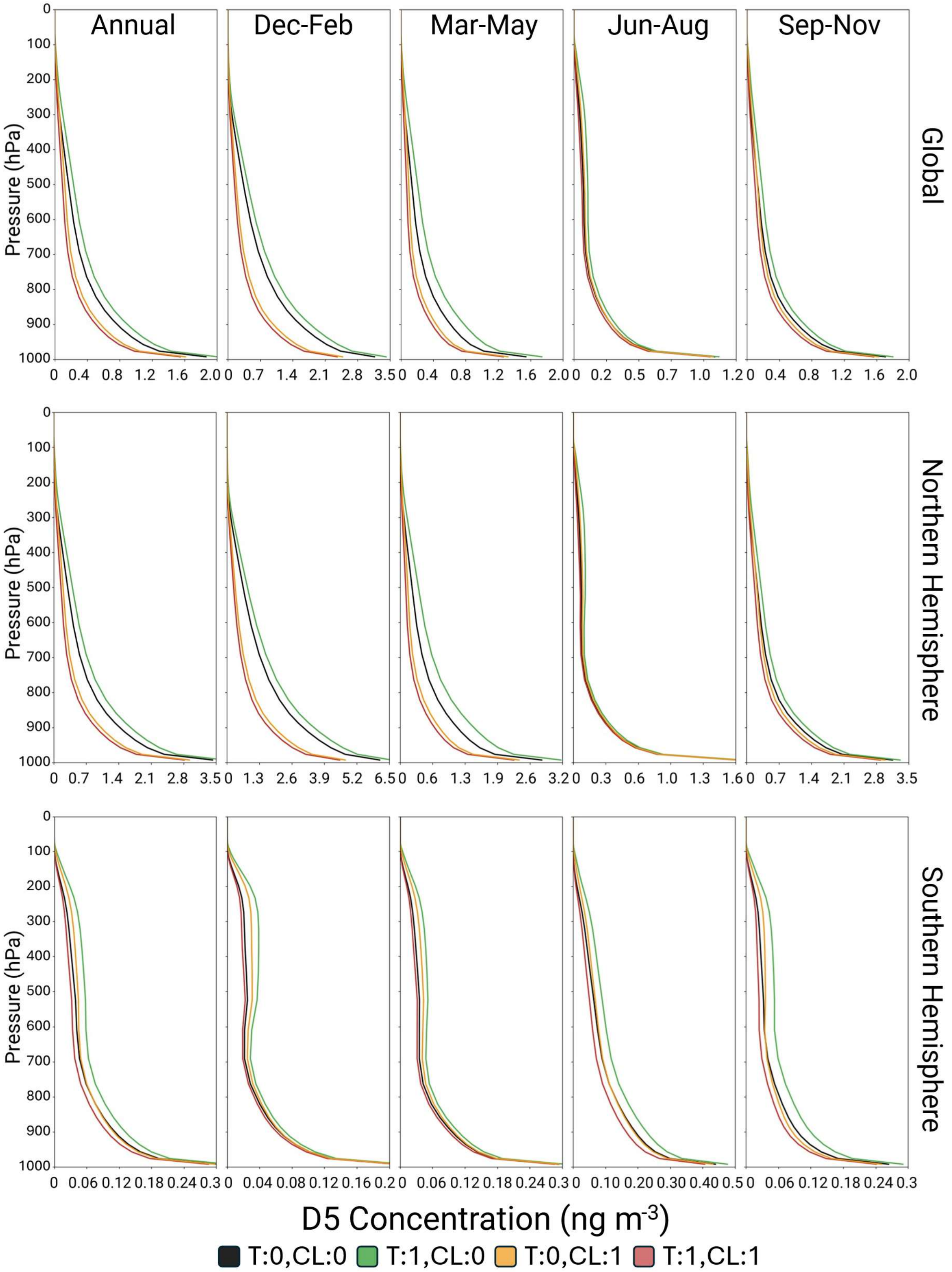

Figure S11: Averaged vertical profiles of global and hemispheric predicted D5 concentrations from the four core model runs by season and for the full calendar year in ng m^{-3}.

Model Comparison to Measurement Data

Here we present the name, coordinates, sampling method, and citations for the 184 study sites identified for comparison to the T:1,CL:1 model (Table S13). In addition, we provide time series comparisons of D5 concentrations predicted by the T:1,CL:1 model and measurements reported in the literature for all sites where measurement data were reported by two or more studies. We note that data are plotted based on calendar date regardless of the year that the concentrations were measured or modeled (e.g. 1/5/2017 and 1/5/2019 are plotted in the same position on the X-axis). Model outputs and measurements from active samplers are plotted as individual dots since each model output represents a daily average and active samples were typically composited over 12 to 48 hours. Measurements from passive samplers (which were typically deployed for several months) are shown as solid bars encompassing the period that the samplers were deployed. Model-observation linear regressions between the predicted and measured concentrations were analyzed and the mean fractional bias (MFB) Eq. (S1), mean fractional error (MFE) Eq. (S2), normalized mean bias (NMB) Eq. (S3), normalized mean error (NME) Eq. (S4), and mean absolute percent error (MAPE) Eq. (S5) were calculated (Table S14-17). In these equations $\overline{P_i}$ represents the mean of the model predicted near-surface concentrations at site i for a given season (active measurements) or sample (passive measurements), $\overline{O_i}$ represents the mean of the measured concentrations at site *i* for a given season or sample, and *N* represents the total number of sites.

Table S13: Study sites used for comparison to the model, their coordinates, sampling method and source.

Location	Lat	Lon	Study	Sampling Method
Alert (1)	82.5	-63.5	Genualdi et al. 2011[13]	Passive
Alert (2)	82.5	-63.5	Rauert et al. 2018[14]	Passive
Alert (3)	82.5	-62.3	Wania et al. 2023[15]	Passive
Alert (4)	82.5	-63.5	Saini et al. 2024[16]	Passive
Algonquin	45.6	-78.5	Wania et al. 2023[15]	Passive
Andoya	69.1	15.8	Wania et al. 2023[15]	Passive
Arauca	7.0	-70.7	Saini et al. 2024[16]	Passive
Barcelona	41.4	2.2	Companioni-Damas et al. 2014[17]	Active
Barrow (1)	71.3	-156.6	Genualdi et al. 2011[13]	Passive
Barrow (2)	71.3	-156.6	Rauert et al. 2018[14]	Passive
Barrow (3)	71.3	-156.6	Saini et al. 2024[16]	Passive
Beijing (1)	39.9	116.3	Jiang et al. 2022[18]	Active
Beijing (2)	40.2	116.0	Jiang et al. 2022[18]	Active
Birkenes	58.4	8.3	Wania et al. 2023[15]	Passive
Blaebjerg	56.3	8.4	Wania et al. 2023[15]	Passive
Borden	44.3	-79.9	Wania et al. 2023[15]	Passive
Boulder	40.0	-105.3	Coggon et al. 2018[19]	Active
Bratt's Lake (1)	50.2	-104.7	Genualdi et al. 2011[13]	Passive
Bratt's Lake (2)	50.2	-104.7	Rauert et al. 2018[14]	Passive
Bratt's Lake (3)	50.2	-104.7	Saini et al. 2024[16]	Passive
Bukit Kototabang	0.2	100.3	Saini et al. 2024[16]	Passive

Cape Grim (1)	40.7	144.7	Genualdi et al. 2011[13]	Passive
Cape Grim (2)	40.7	144.7	Rauert et al. 2018[14]	Passive
Cedar Rapids	42.0	-91.7	Yucuis et al. 2013[20]	Active
Chacaltaya	-16.4	-68.1	Saini et al. 2024[16]	Passive
Chicago	41.8	-87.6	Yucuis et al. 2013[20]	Active
Coral Harbour	37.1	-6.6	Saini et al. 2024[16]	Passive
Danum Valley	5.0	117.8	Saini et al. 2024[16]	Passive
Darwin	-12.4	130.9	Saini et al. 2024[16]	Passive
Doñana National Park	41.3	-72.1	Saini et al. 2024[16]	Passive
Downsview (1)	43.8	-50.1	Genualdi et al. 2011[13]	Passive
Downsview (2)	43.8	-79.5	Wania et al. 2023[15]	Passive
Egbert / CARE Station	44.2	-79.8	Saini et al. 2024[16]	Passive
Fraserdale (1)	49.9	-81.6	Genualdi et al. 2011[13]	Passive
Fraserdale (2)	49.9	-81.6	Rauert et al. 2018[14]	Passive
Fraserdale (3)	49.9	-81.6	Wania et al. 2023[15]	Passive
Fraserdale (4)	49.9	-81.6	Saini et al. 2024[16]	Passive
Gortinak	70.2	28.7	Wania et al. 2023[15]	Passive
Gosan, Jeju Island	33.3	126.2	Saini et al. 2024[16]	Passive
Groton (1)	41.3	-72.1	Genualdi et al. 2011[13]	Passive
Groton (2)	41.3	-72.1	Rauert et al. 2018[14]	Passive
Hanoi (1)	21.0	105.9	Anh et al. 2021[21]	Active
Hanoi (2)	21.0	105.9	Anh et al. 2021[21]	Active
Hanoi (3)	21.0	105.9	Anh et al. 2021[21]	Active
Hanoi (4)	21.0	105.9	Anh et al. 2021[21]	Active
Hanoi (5)	21.0	105.9	Anh et al. 2021[21]	Active
Hanoi (6)	21.0	105.9	Anh et al. 2021[21]	Active
Hanoi (7)	21.0	105.9	Anh et al. 2021[21]	Active
Hanoi (8)	21.0	105.9	Anh et al. 2021[21]	Active
Hanoi (9)	21.0	105.9	Anh et al. 2021[21]	Active
Hanoi (10)	21.0	105.9	Anh et al. 2021[21]	Active
Hanoi (11)	21.0	105.9	Anh et al. 2021[21]	Active
Hanoi (12)	21.0	105.9	Anh et al. 2021[21]	Active
Hanoi (13)	21.0	105.8	Anh et al. 2021[21]	Active
Hanoi (14)	21.0	105.9	Anh et al. 2021[21]	Active
Hanoi (15)	21.0	105.9	Anh et al. 2021[21]	Active
Hanoi (16)	21.0	105.9	Anh et al. 2021[21]	Active
Hanoi (17)	21.0	105.9	Anh et al. 2021[21]	Active
Hanoi (18)	21.0	105.8	Anh et al. 2021[21]	Active
Hanoi (19)	21.0	105.8	Anh et al. 2021[21]	Active
Hanoi (20)	21.0	105.9	Anh et al. 2021[21]	Active
Hanoi (21)	21.0	105.8	Anh et al. 2021[21]	Active
Hanoi (22)	21.0	105.8	Anh et al. 2021[21]	Active
Hanoi (23)	21.0	105.8	Anh et al. 2021[21]	Active

Hanoi (24)	21.0	105.8	Anh et al. 2021[21]	Active
Hanoi (25)	21.0	105.8	Anh et al. 2021[21]	Active
Hanoi (26)	21.0	105.8	Anh et al. 2021[21]	Active
Hanoi (27)	21.0	105.8	Anh et al. 2021[21]	Active
Hanoi (28)	21.0	105.8	Anh et al. 2021[21]	Active
Hanoi (29)	21.0	105.8	Anh et al. 2021[21]	Active
Hanoi (30)	21.0	105.8	Anh et al. 2021[21]	Active
Hanoi (31)	21.0	105.8	Anh et al. 2021[21]	Active
Hanoi (32)	21.0	105.8	Anh et al. 2021[21]	Active
Hanoi (33)	21.0	105.8	Anh et al. 2021[21]	Active
Hanoi (34)	21.0	105.8	Anh et al. 2021[21]	Active
Hanoi (35)	21.0	105.9	Anh et al. 2021[21]	Active
Hanoi (36)	21.0	105.9	Anh et al. 2021[21]	Active
Hanoi (37)	21.0	105.8	Anh et al. 2021[21]	Active
Hanoi (38)	21.0	105.8	Anh et al. 2021[21]	Active
Hilo (1)	19.5	-155.6	Genualdi et al. 2011[13]	Passive
Hilo (2)	19.5	-155.6	Rauert et al. 2018[14]	Passive
Itatiaia	-22.4	-44.7	Saini et al. 2024[16]	Passive
Izana	64.2	-83.3	Saini et al. 2024[16]	Passive
Izmir (1)	39.3	27.1	Okan et al. 2021[22]	Passive
Izmir (10)	38.4	27.2	Okan et al. 2021[22]	Passive
Izmir (11)	38.4	27.0	Okan et al. 2021[22]	Passive
Izmir (12)	38.5	27.1	Okan et al. 2021[22]	Passive
Izmir (13)	38.5	27.1	Okan et al. 2021[22]	Passive
Izmir (14)	38.5	27.1	Okan et al. 2021[22]	Passive
Izmir (15)	38.4	27.1	Okan et al. 2021[22]	Passive
Izmir (16)	38.4	27.1	Okan et al. 2021[22]	Passive
Izmir (17)	38.4	27.2	Okan et al. 2021[22]	Passive
Izmir (18)	38.5	27.2	Okan et al. 2021[22]	Passive
Izmir (2)	39.3	27.1	Okan et al. 2021[22]	Passive
Izmir (3)	39.3	27.1	Okan et al. 2021[22]	Passive
Izmir (4)	38.4	27.2	Okan et al. 2021	Passive
Izmir (5)	38.4	27.2	Okan et al. 2021[22]	Passive
Izmir (6)	38.4	27.2	Okan et al. 2021[22]	Passive
Izmir (7)	38.4	27.1	Okan et al. 2021[22]	Passive
Izmir (8)	38.4	27.2	Okan et al. 2021[22]	Passive
Izmir (9)	38.4	27.2	Okan et al. 2021[22]	Passive
Karvatn	59.9	8.7	Wania et al. 2023[15]	Passive
Košetice (1)	49.6	15.1	Genualdi et al. 2011[13]	Passive
Košetice (2)	49.6	15.1	Rauert et al. 2018[14]	Passive
Košetice (3)	49.6	15.1	Saini et al. 2024[16]	Passive
Kunming (1)	24.9	102.8	Jiang et al. 2022[18]	Active
Kunming (2)	24.7	102.8	Jiang et al. 2022[18]	Active

Kuujiuaq	58.1	-68.5	Wania et al. 2023[15]	Passive
Lijian (1)	26.9	100.2	Jiang et al. 2022[18]	Active
LiJian (2)	26.9	100.1	Jiang et al. 2022[18]	Active
Little Fox Lake	61.4	-135.6	Genualdi et al. 2011[13]	Passive
Little Fox Lake (2)	61.4	-135.6	Rauert et al. 2018[14]	Passive
Little Fox Lake (3)	61.4	-135.6	Saini et al. 2024[16]	Passive
Malin Head (1)	53.4	-7.3	Genualdi et al. 2011[13]	Passive
Malin Head (2)	55.4	-7.3	Saini et al. 2024[16]	Passive
Malin Head (3)	53.4	-7.3	Rauert et al. 2018[14]	Passive
Manila	14.7	121.1	Saini et al. 2024[16]	Passive
Manizales	5.1	-75.4	Saini et al. 2024[16]	Passive
Mauna Loa Obs	19.5	-155.6	Saini et al. 2024[16]	Passive
Mendoza Province	-32.7	-68.4	Saini et al. 2024[16]	Passive
Mount Revelstoke	51.1	-118.1	Rauert et al. 2018[14]	Passive
New York City (1)	40.8	-73.9	Brunet et al. 2024[23]	Active
New York City (2)	40.8	-73.9	Coggon et al. 2021[24]	Active
Ny-Ålesund (1)	78.9	11.9	Genualdi et al. 2011[13]	Passive
Ny-Ålesund (2)	78.9	11.9	Rauert et al. 2018[14]	Passive
Ny-Ålesund (4)	78.9	11.9	Saini et al. 2024[16]	Passive
Paris (1)	48.9	2.4	Genualdi et al. 2011[13]	Passive
Paris (2)	48.8	2.4	Verma et al. 2024[25]	Active
Paris (3)	48.9	2.4	Rauert et al. 2018[14]	Passive
Paris (4)	48.9	2.4	Saini et al. 2024[16]	Passive
Pierre Auger Observatory	-35.1	-65.6	Saini et al. 2024[16]	Passive
Point Reyes (1)	38.0	-122.8	Genualdi et al. 2011[13]	Passive
Point Reyes (2)	38.0	-122.8	Saini et al. 2024[16]	Passive
Point Reyes (3)	38.0	-122.8	Rauert et al. 2018[14]	Passive
Ragged Point	13.2	-59.4	Saini et al. 2024[16]	Passive
Rural Sweden (1)	59.3	15.5	McLachlan et al. 2010[26]	Active
Rural Sweden (2)	58.8	17.2	Kierkegaard et al. 2013[27]	Active
Sable Island (1)	43.9	-60.0	Genualdi et al. 2011[13]	Passive
Sable Island (2)	43.6	-60.0	Rauert et al. 2018[14]	Passive
Saitama (1)	35.8	139.8	Horii et al. 2021[28]	Active
Saitama (2)	35.9	139.7	Horii et al. 2021[28]	Active
Saitama (3)	35.8	139.6	Horii et al. 2021[28]	Active
Saitama (4)	35.9	139.5	Horii et al. 2021[28]	Active
Saitama (5)	35.9	139.3	Horii et al. 2021[28]	Active
Saitama (6)	36.1	139.6	Horii et al. 2021[28]	Active
Saitama (7)	36.1	139.7	Horii et al. 2021[28]	Active
Saitama (8)	36.2	139.3	Horii et al. 2021[28]	Active
Saitama (9)	36.0	139.2	Horii et al. 2021[28]	Active
São Jose dos Ausentes	-28.6	-49.8	Saini et al. 2024[16]	Passive
São Luis do Maranha	-2.6	-44.2	Saini et al. 2024[16]	Passive

St. Lawrence Island	63.7	-170.5	Saini et al. 2024[16]	Passive
Stórhöfði (1)	63.4	-20.3	Genualdi et al. 2011[13]	Passive
Stórhöfði (2)	63.4	-20.3	Rauert et al. 2018[14]	Passive
Stórhöfði (3)	63.4	-20.3	Saini et al. 2024[16]	Passive
Sydney (1)	28.0	-82.2	Genualdi et al. 2011[13]	Passive
Sydney (2)	28.0	-82.2	Rauert et al. 2018[14]	Passive
Sydney (3)	28.0	-82.2	Saini et al. 2024[16]	Passive
Temple Basin	-42.9	171.6	Saini et al. 2024[16]	Passive
Toronto (1)	43.8	-79.5	Ahrens et al. 2014	Active
Toronto (2)	43.8	-79.5	Rauert et al. 2018[14]	Passive
Toronto (3)	43.7	-79.4	Wania et al. 2023[15]	Passive
Toronto (4)	43.8	-79.5	Coggon et al. 2018	Active
Toronto (5)	43.8	-79.5	Saini et al. 2024[16]	Passive
Tudor Hill (1)	32.4	-64.6	Genualdi et al. 2011[13]	Passive
Tudor Hill (2)	32.4	-64.6	Rauert et al. 2018[14]	Passive
Tula	-14.2	-170.6	Saini et al. 2024[16]	Passive
Tustervatn	65.8	13.9	Wania et al. 2023[15]	Passive
Ucluelet (1)	48.9	-125.5	Genualdi et al. 2011[13]	Passive
Ucluelet (2)	48.9	-135.5	Rauert et al. 2018[14]	Passive
Ucluelet (3)	48.9	-125.5	Saini et al. 2024[16]	Passive
Uetliberg	47.4	8.5	Buser et al. 2013	Active
Warsaw Caves	44.5	-78.1	Saini et al. 2024[16]	Passive
Weilerswist	50.7	6.8	Wania et al. 2023[15]	Passive
West Branch	41.7	-91.3	Yucuis et al. 2013[20]	Active
Whistler (1)	50.1	-122.9	Genualdi et al. 2011[13]	Passive
Whistler (2)	50.1	-122.9	Rauert et al. 2018[14]	Passive
Whistler (3)	50.1	-123.0	Saini et al. 2024[16]	Passive
Yaba	6.5	3.4	Saini et al. 2024[16]	Passive
Zeppelin (1)	78.5	11.5	Krogseth et al. 2013[29]	Active
Zeppelin (2)	78.9	11.9	Wania et al. 2023[15]	Passive
Zeppelin (3)	79.0	12.0	Warner et al. 2020[30]	Active
Zhangjiagang (1)	31.9	120.6	Jiang et al. 2022[18]	Active
Zhangjiagang (2)	31.9	120.8	Jiang et al. 2022[18]	Active
Zurich	47.4	8.5	Buser et al. 2013[31]	Active

$$(Eq.\ S1)\ MFB = 100 * \left(\frac{2}{N} * \sum \frac{(\overline{P_i} - \overline{O_i})}{(\overline{P_i} + \overline{O_i})}\right)$$

$$(Eq.S2)\ MFE = 100 * \left(\frac{2}{N} * \sum \frac{|\overline{P_i} - \overline{O_i}|}{(\overline{P_i} + \overline{O_i})}\right)$$

$$(Eq.\ S3)\ NMB = \frac{\sum(\overline{P_i} - \overline{O_i})}{\sum \overline{O_i}}$$

$$(Eq.S4)\ NME = \frac{\sum|\overline{P_i} - \overline{O_i}|}{\sum \overline{O_i}}$$

$$(Eq.S5)\ MAPE = 100 * \left(\frac{1}{N} * \sum \left|\frac{(\overline{P_i} - \overline{O_i})}{\overline{O_i}}\right|\right)$$

Table S14: Statistical metrics from the comparison of D4, D5, and D6 near-surface concentrations predicted by the T:0,Cl:0 model and atmospheric measurements reported in the literature.

	D4		D5		D6	
	Active	Passive	Active	Passive	Active	Passive
Mean Measurement (ng m^{-3})	30	26	75	63	14	6.6
Mean Model (ng m^{-3})	7.7	3.3	38	16	2.0	0.83
Geometric Mean Measurement (ng m^{-3})	11	14	23	20	6.6	1.9
Geometric Mean Model (ng m^{-3})	4.2	1.2	20	4.9	0.98	0.23
R^2	0.53	0.037	0.53	0.38	0.41	0.19
MFB	-0.69	-1.3	-0.13	-0.91	-1.2	-1.2
MFE	1	1.4	0.81	1.1	1.3	1.3
NMB	-0.74	-0.87	-0.49	-0.75	-0.86	-0.87
NME	0.79	0.89	0.62	0.81	0.86	0.9
MAPE	100%	86%	190%	95%	80%	95%

Table S15: Statistical metrics from the comparison of D4, D5, and D6 near-surface concentrations predicted by the T:1,Cl:0 model and atmospheric measurements reported in the literature.

	D4		D5		D6	
	Active	Passive	Active	Passive	Active	Passive
Mean Measurement (ng m^{-3})	30	26	75	63	14	6.6
Mean Model (ng m^{-3})	7.8	3.4	39	16	2.0	0.85
Geometric Mean Measurement (ng m^{-3})	11	14	23	20	6.6	1.9
Geometric Mean Model (ng m^{-3})	4.2	1.4	21	5.5	1	0.26
R^2	0.41	0.035	0.53	0.37	0.41	0.19
MFB	-0.67	-1.3	-0.12	-0.85	-1.2	-1.1
MFE	1	1.4	0.82	1	1.3	1.3
NMB	-0.74	-0.87	-0.49	-0.74	-0.86	-0.87
NME	0.79	0.89	0.61	0.8	0.86	0.9
MAPE	100%	85%	200%	96%	81%	95%

Table S16: Statistical metrics from the comparison of D4, D5, and D6 near-surface concentrations predicted by the T:0,Cl:1 model and atmospheric measurements reported in the literature.

	D4		D5		D6	
	Active	Passive	Active	Passive	Active	Passive
Mean Measurement (ng m^{-3})	30	26	75	63	14	6.6
Mean Model (ng m^{-3})	7.5	3.1	37	15	1.9	0.78
Geometric Mean Measurement (ng m^{-3})	11	14	23	20	6.6	1.9
Geometric Mean Model (ng m^{-3})	3.9	0.93	19	3.7	0.91	0.18
R^2	0.41	0.036	0.56	0.38	0.41	0.19
MFB	-0.74	-1.4	-0.19	-1	-1.3	-1.2
MFE	1	1.5	0.8	1.2	1.3	1.4
NMB	-0.75	-0.88	-0.51	-0.77	-0.86	-0.88
NME	0.79	0.9	0.61	0.82	0.87	0.9
MAPE	100%	88%	170%	97%	80%	97%

Table S17: Statistical metrics from the comparison of D4, D5, and D6 near-surface concentrations predicted by the T:1,Cl:1 model and atmospheric measurements reported in the literature.

	D4		D5		D6	
	Active	Passive	Active	Passive	Active	Passive
Mean Measurement (ng m^{-3})	30	26	75	63	14	6.5
Mean Model (ng m^{-3})	7.5	3.1	37	15	1.9	0.79
Geometric Mean Measurement (ng m^{-3})	11	14	23	20	6.6	1.9
Geometric Mean Model (ng m^{-3})	4.0	1.0	19	4.0	0.93	0.19
R^2	0.41	0.036	0.56	0.38	0.54	0.36
MFB	-0.72	-1.4	-0.17	-1.0	-1.3	-1.2
MFE	1.0	1.5	0.81	1.2	1.3	1.4
NMB	-0.75	-0.88	-0.50	-0.76	-0.86	-0.88
NME	0.79	0.90	0.62	0.81	0.86	0.90
MAPE	102%	87%	180%	96%	80%	97%

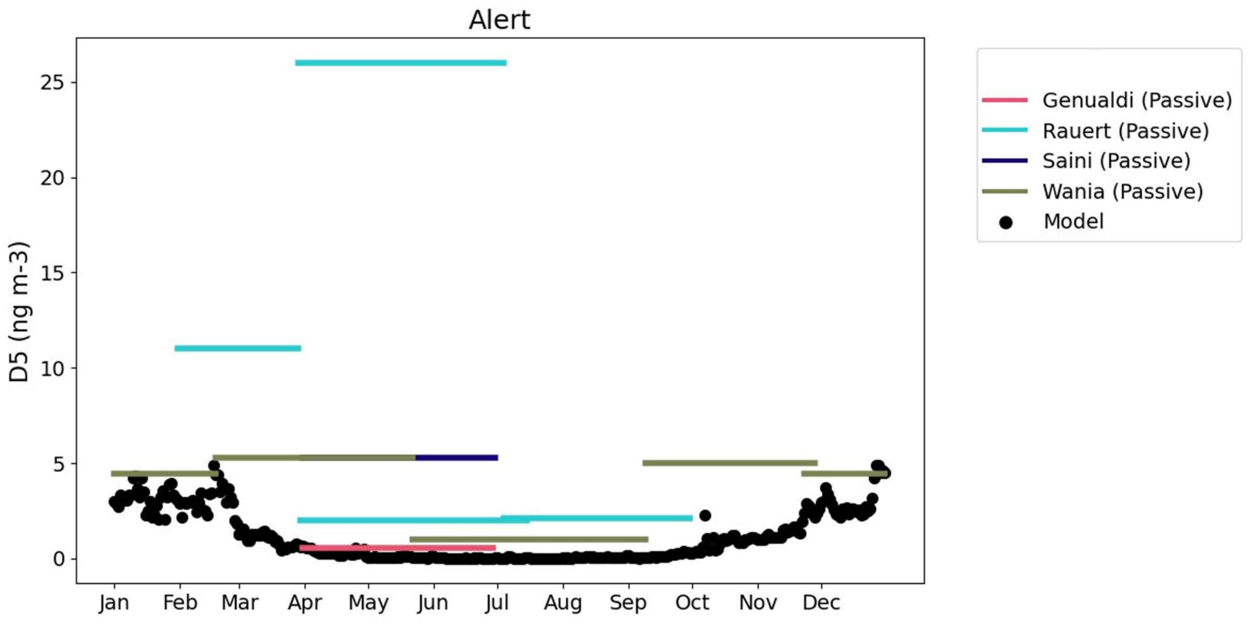

Figure S12: Comparison of predicted D5 near-surface concentrations from the T:1,Cl:1 model and measurements from Genualdi et al. 2011, Rauert et al. 2018, Saini et al. 2023, and Wania et al. 2023 at Alert, Nunavut, Canada (82.5, -63.5).[13-16]

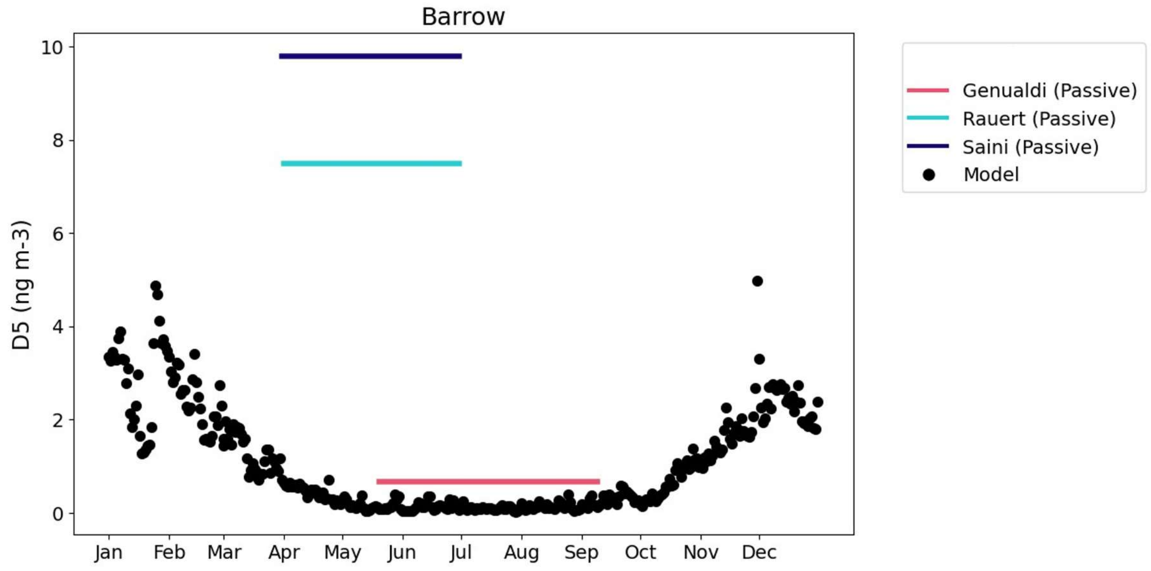

Figure S13: Comparison of predicted D5 near-surface concentrations from the T:1,Cl:1 model and measurements from Genualdi et al. 2011, Rauert et al. 2018, and Saini et al. 2023 at Barrow (now known as Utqiagvik), Alaska, USA (82.5, -63.5). [13, 14, 16]

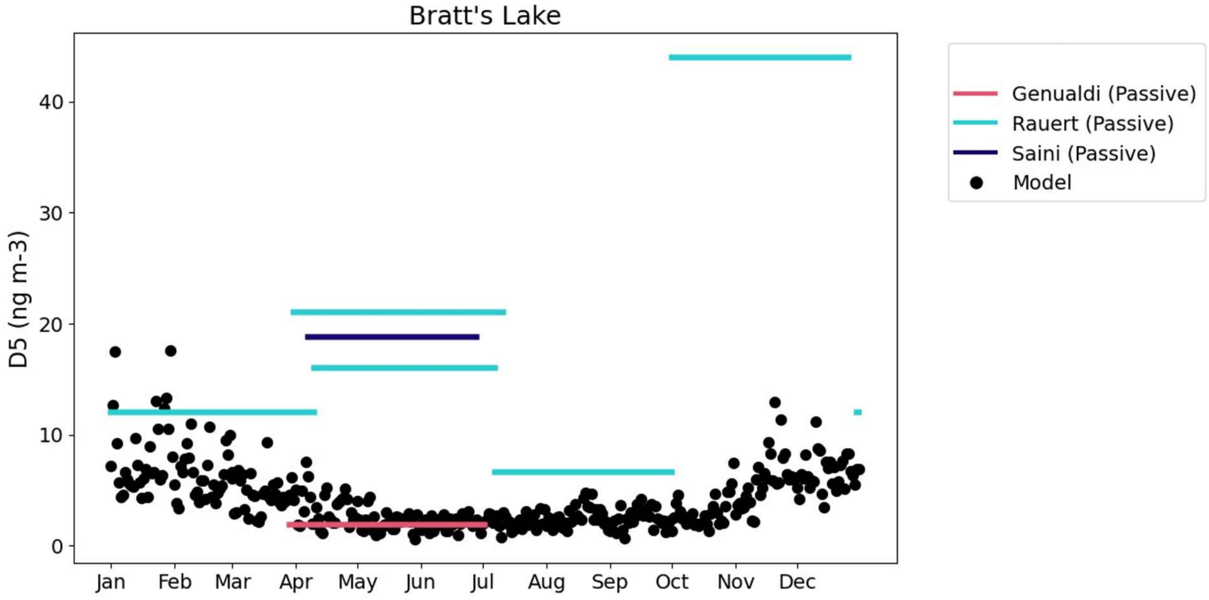

Figure S14: Comparison of predicted D5 near-surface concentrations from the T:1,Cl:1 model and measurements from Genualdi et al. 2011, Rauert et al. 2018, and Saini et al. 2023 at Bratt's Lake, Saskatchewan, Canada (50.2, -104.7).[13, 14, 16]

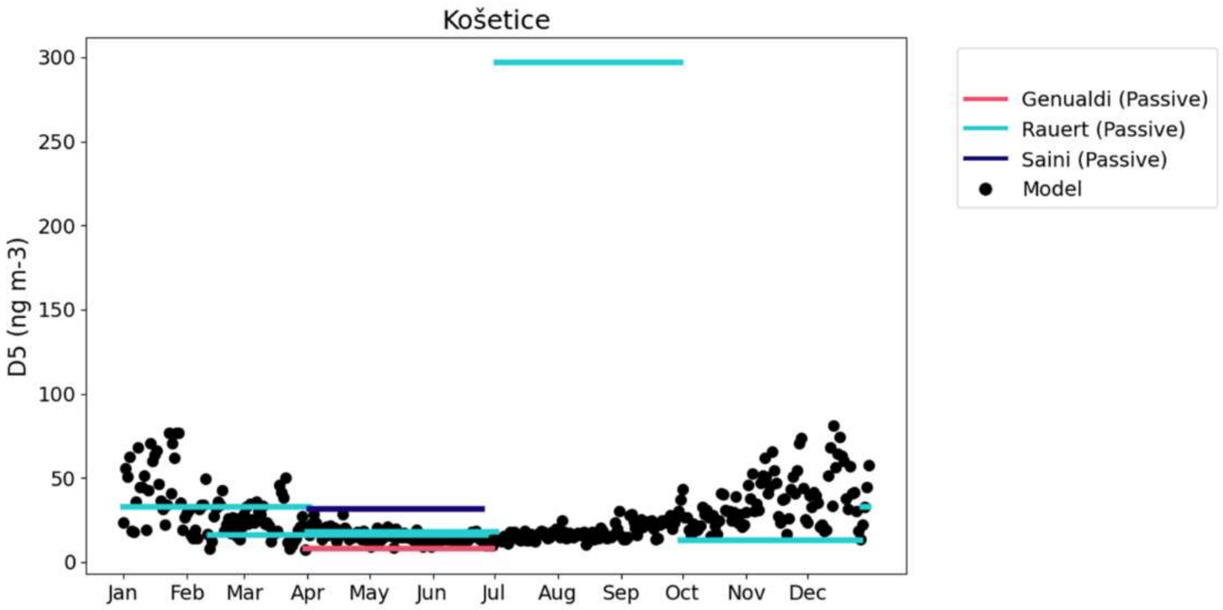

Figure S15: Comparison of predicted D5 near-surface concentrations from the T:1,Cl:1 model and measurements from Genualdi et al. 2011, Rauert et al. 2018, and Saini et al. 2023 at Košetice, Czechia (49.6, 15.1). [13, 14, 16]

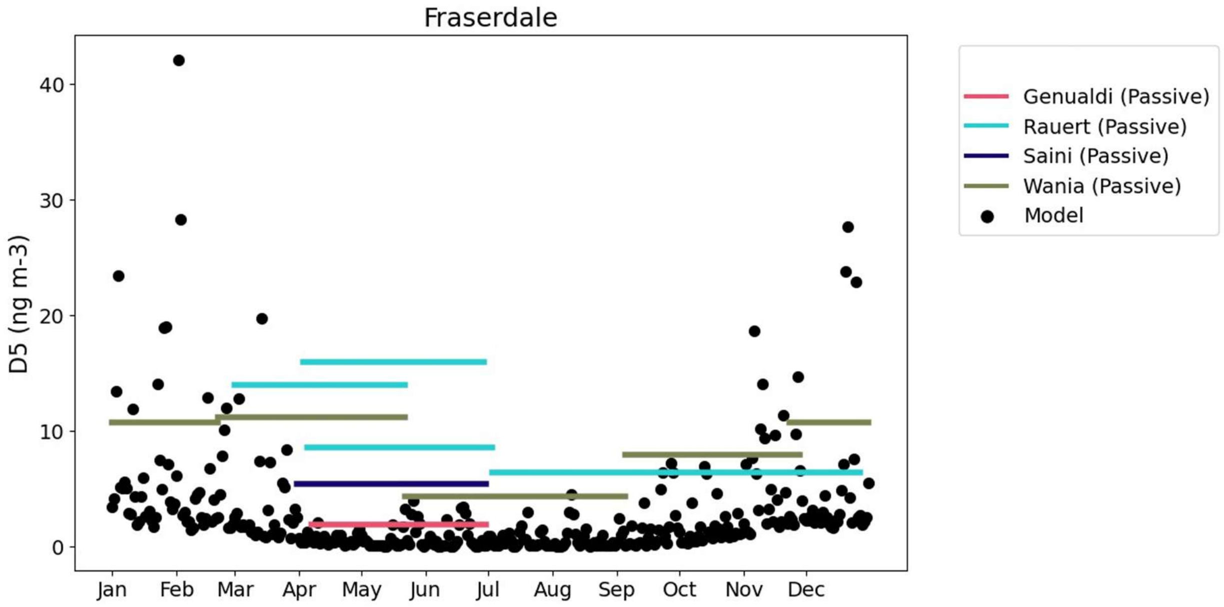

Figure S16: Comparison of predicted D5 near-surface concentrations from the T:1,Cl:1 model and measurements from Genualdi et al. 2011, Rauert et al. 2018, Saini et al. 2023, and Wania et al. 2023 at Fraserdale, Ontario, Canada (49.9, -81.6). [13-16]

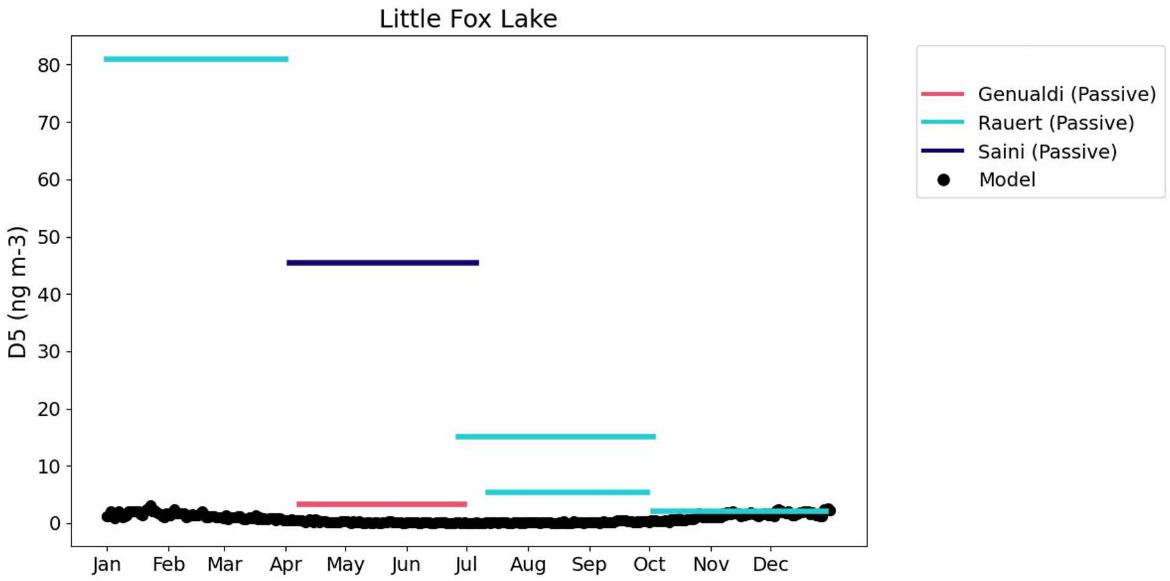

Figure S17: Comparison of predicted D5 near-surface concentrations from the T:1,Cl:1 model and measurements from Genualdi et al. 2011, Rauert et al. 2018, and Saini et al. 2023 at Little Fox Lake, Yukon, Canada (61.4, -135.6). [13-16]

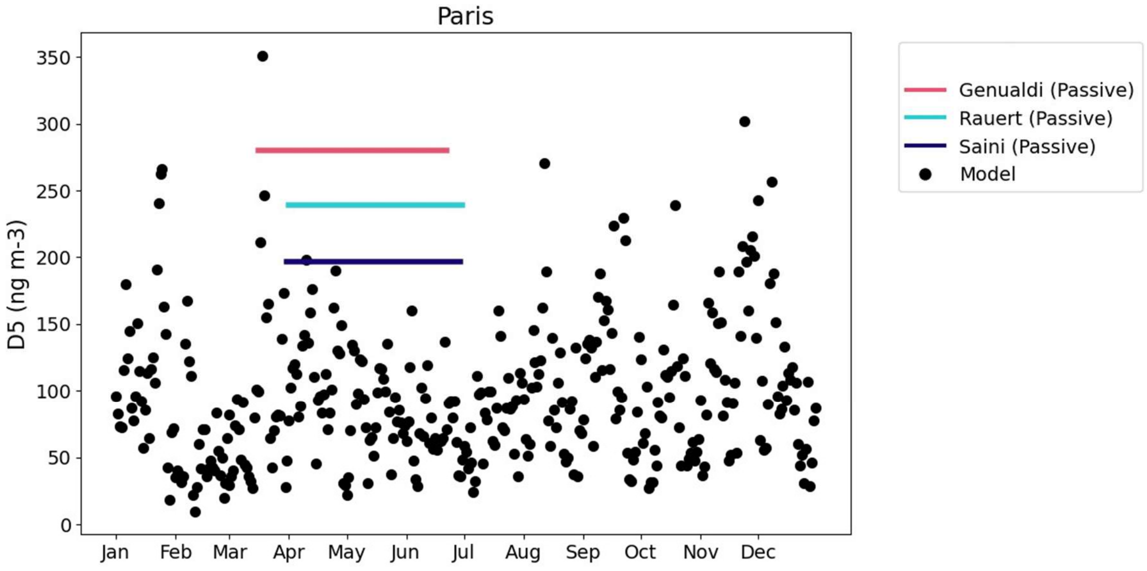

Figure S18: Comparison of predicted D5 near-surface concentrations from the T:1,Cl:1 model and measurements from Genualdi et al. 2011, Rauert et al. 2018, and Saini et al. 2023 at Paris, France (48.9, 2.4). [13, 14, 16, 25]

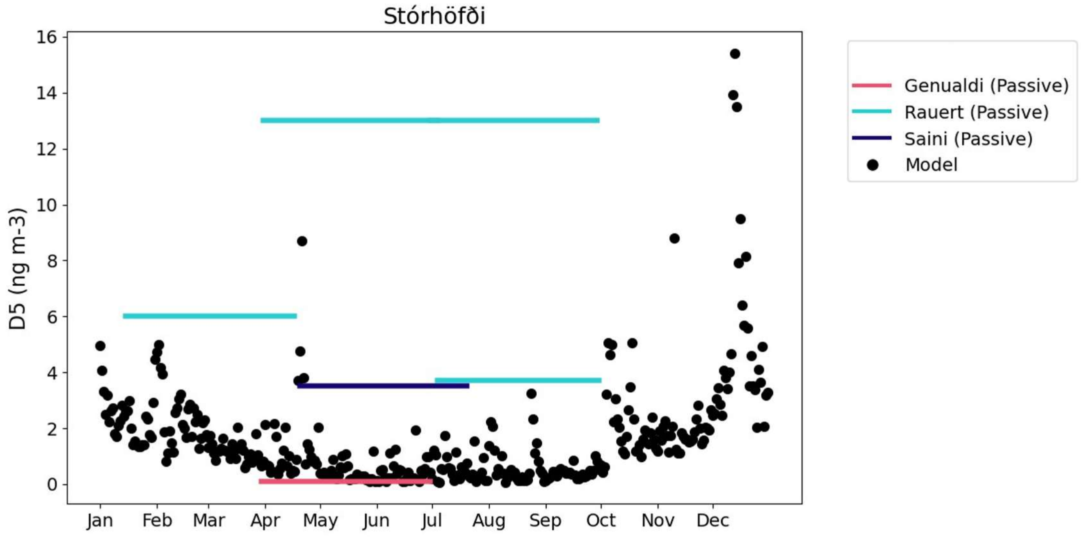

Figure S19: Comparison of predicted D5 near-surface concentrations from the T:1,Cl:1 model and measurements from Genualdi et al. 2011, Rauert et al. 2018, and Saini et al. 2023 at Stórhöfði, Iceland (63.4, -20.3). [13, 14, 16]

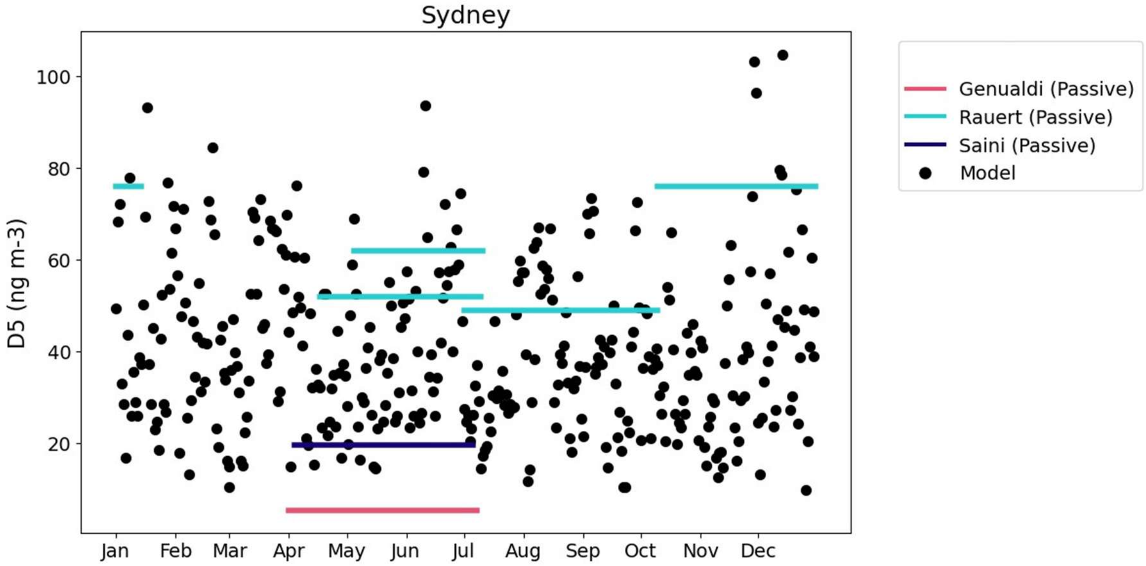

Figure S20: Comparison of predicted D5 near-surface concentrations from the T:1,Cl:1 model and measurements from Genualdi et al. 2011, Rauert et al. 2018, and Saini et al. 2023 at Sydney, Florida, USA (28, -82.2). [13, 14, 16]

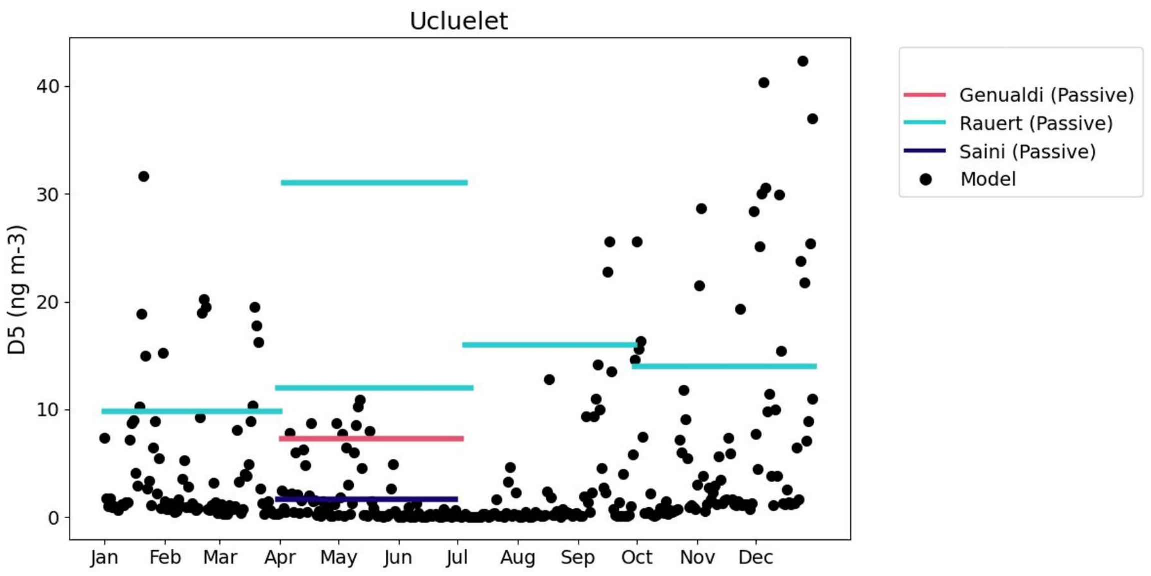

Figure S21: Comparison of predicted D5 near-surface concentrations from the T:1,Cl:1 model and measurements from Genualdi et al. 2011, Rauert et al. 2018, and Saini et al. 2023 at Ucluelet, British Columbia, Canada. [13, 14, 16]

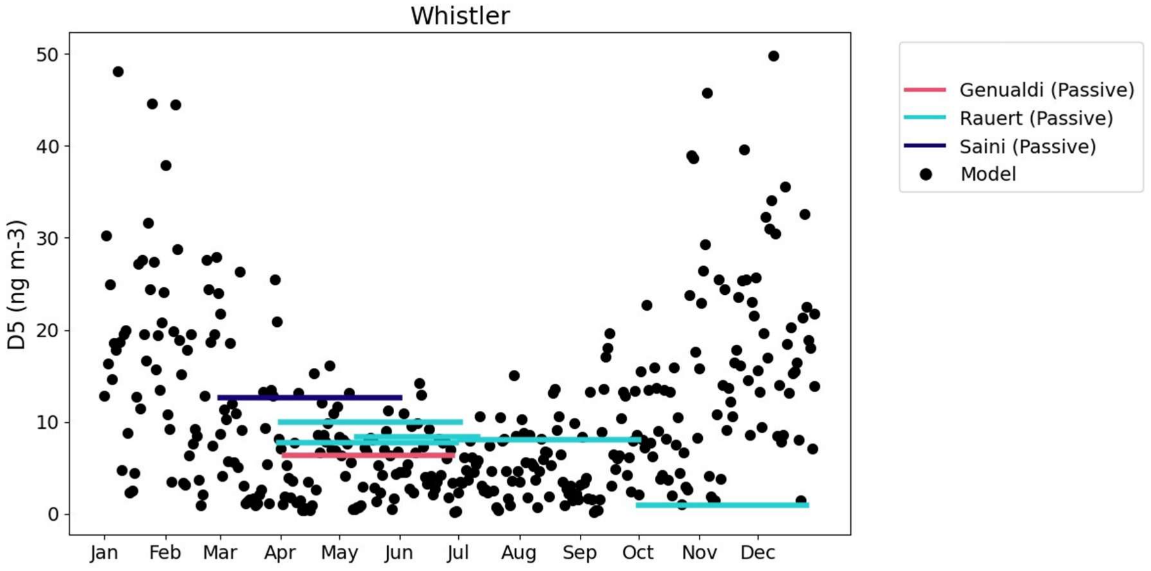

Figure S22: Comparison of predicted D5 near-surface concentrations from the T:1,Cl:1 model and measurements from Genualdi et al. 2011, Rauert et al. 2018, and Saini et al. 2023 at Whistler, British Columbia, Canada (50.1, -122.9). [13, 14, 16]

Instructions For Running the Model.

Should future researchers desire to run or alter our model, we have released the necessary files required to do so to an open access data repository.[12] We also include here instructions on how to setup the model and conduct the model runs used in this study. Items in *italics* denote command lines while items in "quotations" are file names or file pathways.

1. While CESM models are publicly available and can be run on any system with the necessary computing power, we recommend running the model using the National Center for Atmospheric Research's own high performance computing system "Derecho" if you are able to obtain access and computing time. While most of the instructions included here are applicable to running the model on any system, they are written with the assumption that you have access to Derecho.
2. Using the SSH client of your choice, ssh into "derecho.hpc.ucar.edu" and login with your credentials.
3. If you have not previously used the derecho system, create a new directory for your cases using the command: *mkdir /glade/work/$USER/derecho_cases* where $USER is your username assigned in the NCAR system.
4. Navigate to the new directory using the command:
   cd /glade/work/$USER/derecho_cases
5. Clone cesm2.2-asdbranch_slh from github using the command:
   git clone https://github.com/RafaPedroFernandez/CESM cesm2.2-asdbranch_slh
6. Navigate into the cloned model with the command:
   cd cesm2.2-asdbranch_slh
7. Check out externals using the following commands in sequence:
   git checkout cesm2.2-asdbranch_slh
   ./manage_externals/checkout_externals
8. Create a new case using the command below where $User is your username within the NCAR system, *$Project* is the project you wish to assign for computing time, and $CaseName is your desired case name. We recommend using a unique and descriptive name which details the model, compset, and resolution used and the date that the run was conducted.

   /glade/work/$User/derecho_cases/cesm2.2-asdbranch_slh/cime/scripts/create_newcase --case /glade/work/$User/derecho_cases/$CaseName --res f09_f09_mg17 --compset FCnudgedslhvbs --project $Project --run-unsupported

9. Make necessary XML changes. To rerun the cases described in this work, we recommend using all of the XML changes listed in Table S exactly as they appear. However, should users wish to run alternate cases (in particular for a different time period than those used in these studies) they may wish to alter some the changes to variables such as RUN_STARTDATE and STOP_N. The XML changes are currently configured to start the model 60 days prior to the start of 2020 and run through the end of that year. Should users choose to investigate a different time period, we recommend starting the model at least 60 days prior to your period of interest to allow the model to spinup. We also note that several of these XML changes reference other files already located on the Derecho system. If these files are no longer available in the given location or the model is being run on a different system you will need to obtain the new location or your own copies of these files and change the listed pathways accordingly. Because they are not the intellectual property of the

authors of this work, we have not included these files in the data repository. If you have difficulty accessing some of these files, please contact the corresponding author for help. All of these changes can be made using a command of the structure below where $Variable is the XML variable being change and $Value is the value being assigned to it:
./xmlchange $Variable=$Value

Table S18: Recommended Model XML Changes

Variable	Value
SSTICE_DATA_FILENAME	/glade/campaign/acom/acom-climate/WACCM-FORECAST/cesm2_sstice/sst_HadOIBl_bc_0.9x1.25_1850_2023_c220203.nc
SSTICE_YEAR_END	2023
SSTICE_GRID_FILENAME	/glade/campaign/cesm/cesmdata/inputdata/share/domains/domain.ocn.fv0.9x1.25_gx1v7.151020.nc
SSTICE_YEAR_ALIGN	1850
SSTICE_YEAR_START	1850
--subgroup case.run JOB_WALLCLOCK_TIME	12:00:00
STOP_OPTION	ndays
STOP_N	30
RESUBMIT	15
RUN_STARTDATE	2019-11-02
CALENDAR	GREGORIAN
START_TOD	00000
RUN_REFDATE	2019-11-02
RUN_REFTOD	00000
PIO_VERSION	1
NTASKS	1024
RUN_TYPE	startup

10. Download the following files from the data repository and create new directories for them as desired such as “emissions” and “mechanisms.”
    a. user_nl_cam
    b. user_nl_clm
    c. seq_drydep_mod.F90
    d. The 4 chem_mech.in files
        i. T0CL0_chem_mech.in
        ii. T1CL0_chem_mech.in
        iii. T0CL1_chem_mech.in
        iv. T1CL1_chem_mech.in
    e. The 3 VMS emissions files
        i. emisssions-D4_surface_0.9x1.25_20241217.nc
        ii. emisssions-D5_surface_0.9x1.25_20241217.nc
        iii. emisssions-D6_surface_0.9x1.25_20241217.nc
11. Select the desired chemical mechanism using the XML change command below where $path is the full pathway to your desired chemical mechanism file.

./xmlchange CAM_CONFIG_OPTS="-phys cam6 -chem trop_strat_mam4_vbs -chem trop_strat_mam4_slhvbs -age_of_air_trcs -usr_mech_infile $path"

There are 4 possible chemical mechanisms which are available in the data repository and can be selected. "T0CL0_chem_mech.in" includes only OH oxidation and does not have temperature dependent reaction rates. "T0Cl1_chem_mech.in" includes both OH and CL oxidation but does not have temperature dependent reaction rates. "T1CL0_chem_mech.in" includes only OH oxidation but has temperature dependent reactions rates. "T1CL1_chem_mech.in" includes both OH and CL oxidation and uses temperature dependent reaction rates for the OH reactions. Except for the changes to the VMS chemistry, these four mechanism files are identical.

12. Setup the case using the following command: *./case.setup*
13. Replace the user_nl_cam file in the case directory with the user_nl_cam available in the data repository and open it with a text editor for modification.
14. Like many of the XML changes, this file references several files already available on Derecho. If these files are not accessible, you will need to obtain a copy or their new pathways and change the pathway to this file accordingly. We also note that this file contains pathways to initial conditions and emissions files which may not contain data for all time periods of interest. If you have altered the start date of the run or its duration, you made need to obtain alternate input files which match your period of interest.
15. Navigate to srf_emis_specifier section of the user_nl_cam file and locate the lines starting "D4", "D5", and "D6" at the end of this section. Replace the $pathtoemissions text with the complete pathway to the appropriate emissions files for D4, D5, and D6 downloaded from the data repository.
16. Replace the user_nl_clm in the case directory with the user_nl_clm file available in the data repository. As above, this file also references an input available on the derecho system which will need to be obtained if not available.
17. Place the seq_drydep_mod.F90 file obtained from the data repository and place it in "$case/SourceMods/src.share" folder where "$case" is the pathway to the created case.
18. Next, build the case using the following command. This process may take 20 to 30 minutes to complete.
    qcmd -A $Project-- ./case.build
19. Finally, submit the case using the command. The case may take several hours to run.
    ./case.submit
20. Upon completion of the case, netcdf files containing the model outputs for the atmospheric compartment can be found in "derecho/scratch/$User/archive/$Case/atm/hist/" where $User is your username in the NCAR system and $Case is the name of the case. The files listed here will contain the name of the case followed by the text "h0" (denoting that they are monthly averages) or "h1" (denoting that they are daily averages). Hourly averages or other temporal resolutions may be saved by altering the user_nl_cam file prior to running the case although saving the model outputs at higher than daily resolution will require a considerable amount of memory.
21. Further information about what these changes and files do and how to alter them can be found at the following web pages.

https://wiki.ucar.edu/display/camchem/Run+CESM+with+Chemistry+on+Derecho

https://wiki.ucar.edu/display/camchem/Updating+Gas-Phase+Chemistry

References

(1) *Estimation Programs Interface Suite™ for Microsoft® Windows*; United States Environmental Protection Agency: Washington, DC, USA., 2012. (accessed 6/13/2024).

(2) Voronkov, M. G.; Klyuchnikov, V. A.; Mironenko, E. V.; Shvets, G. N.; Danilova, T. F.; Khudobin, Y. I. Thermochemistry of organosilicon compounds: V. Thermochemical properties of perorganyloligocyclosiloxanes. *Journal of Organometallic Chemistry* **1991**, *406* (1), 91-97. DOI: 10.1016/0022-328X(91)83174-3.

(3) Stephenson, R. M.; Malanowski, S.; Stephenson, R. M.; Malanowski, S. Properties of organic compounds. *Handbook of the Thermodynamics of Organic Compounds* **1987**, 1-471.

(4) *Percepta Platform PhysChem Module v14.00*; Advanced Chemistry Development, Inc. (ACD/Labs): Toronto, ON, Canada, (accessed 6/22/2024).

(5) 2,4,4,6,6,8,8,10,10-Nonamethyl-1,3,5,7,9,2,4,6,8,10-pentoxapentasilecan-2-ol. ChemSpider: 2024.

(6) 2,4,4,6,6,8,8,10,10,12,12-Undecamethylcyclohexasiloxan-2-ol. ChemSpider: 2024.

(7) Alton, M. W.; Browne, E. C. Atmospheric Chemistry of Volatile Methyl Siloxanes: Kinetics and Products of Oxidation by OH Radicals and Cl Atoms. *Environmental Science & Technology* **2020**, *54* (10), 5992-5999. DOI: 10.1021/acs.est.0c01368.

(8) Bernard, F. o.; Papanastasiou, D. K.; Papadimitriou, V. C.; Burkholder, J. B. Temperature dependent rate coefficients for the gas-phase reaction of the OH radical with linear (L2, L3) and cyclic (D3, D4) permethylsiloxanes. *The Journal of Physical Chemistry A* **2018**, *122* (17), 4252-4264. DOI: 10.1021/acs.jpca.8b01908.

(9) Link, M. F.; Pothier, M. A.; Vermeuel, M. P.; Riches, M.; Millet, D. B.; Farmer, D. K. In-Canopy Chemistry, Emissions, Deposition, and Surface Reactivity Compete to Drive Bidirectional Forest-Atmosphere Exchange of VOC Oxidation Products. *ACS ES&T Air* **2024**, *1* (4), 305-315. DOI: 10.1021/acsestair.3c00074.

(10) Janechek, N. J.; Hansen, K. M.; Stanier, C. O. Comprehensive atmospheric modeling of reactive cyclic siloxanes and their oxidation products. *Atmospheric Chemistry and Physics* **2017**, *17* (13), 8357-8370. DOI: 10.5194/acp-17-8357-2017.

(11) Fernandez, R. P.; Cuevas, C. A.; Villamayor, J.; Feinberg, A.; Kinnison, D. E.; Vitt, F.; Bossolasco, A.; Barrera, J. A.; Li, Q.; Saiz-Lopez, A. Short-Lived Halogen Emissions and Chemistry in the Community Earth System Model v2 (CESM2-SLH). *In Preperation* **2025**.

(12) Brunet, C. E. M., S.; Roozitalab, B.; Gibson, N. K.; Fernandez, R. P.; Saiz-Lopez, A.; Hornbuckle, K.C.; Stanier, C.O. Dataset for Modeled Impacts of Chlorine Oxidation and Temperature Dependence on the Atmospheric Lifetime and Concentrations of Volatile Methyl Siloxanes. Iowa Research Online, 2025. DOI: 10.25820/data.007521

(13) Genualdi, S.; Harner, T.; Cheng, Y.; MacLeod, M.; Hansen, K. M.; van Egmond, R.; Shoeib, M.; Lee, S. C. Global Distribution of Linear and Cyclic Volatile Methyl Siloxanes in Air. *Environmental Science & Technology* **2011**, *45* (8), 3349-3354. DOI: 10.1021/es200301j.

(14) Rauert, C.; Shoieb, M.; Schuster, J. K.; Eng, A.; Harner, T. Atmospheric concentrations and trends of poly- and perfluoroalkyl substances (PFAS) and volatile methyl siloxanes (VMS) over 7 years of sampling in the Global Atmospheric Passive Sampling (GAPS) network. *Environmental Pollution* **2018**, *238*, 94-102. DOI: 10.1016/j.envpol.2018.03.017.

(15) Wania, F.; Warner, N. A.; McLachlan, M. S.; Durham, J.; Miøen, M.; Lei, Y. D.; Xu, S. Seasonal and latitudinal variability in the atmospheric concentrations of cyclic volatile methyl siloxanes in the Northern

Hemisphere. *Environmental Science: Processes & Impacts* **2023**, *25* (3), 496-506. DOI: 10.1039/D2EM00467D.

(16) Saini, A.; Chinnadurai, S.; Schuster, J. K.; Eng, A.; Harner, T. Per-and polyfluoroalkyl substances and volatile methyl siloxanes in global air: Spatial and temporal trends. *Environmental Pollution* **2023**, *323*, 121291. DOI: 10.1016/j.envpol.2023.121291.

(17) Companioni-Damas, E.; Santos, F.; Galceran, M. Linear and cyclic methylsiloxanes in air by concurrent solvent recondensation–large volume injection–gas chromatography–mass spectrometry. *Talanta* **2014**, *118*, 245-252. DOI: 10.1016/j.talanta.2013.10.020.

(18) Jiang, Y.; Guo, J.; Zhou, Y.; Zhang, B.; Zhang, J. Occurrence and Behavior of Methylsiloxanes in Urban Environment in Four Cities of China. *International Journal of Environmental Research and Public Health* **2022**, *19* (21), 13869. DOI: 10.3390/ijerph192113869.

(19) Coggon, M. M.; McDonald, B. C.; Vlasenko, A.; Veres, P. R.; Bernard, F.; Koss, A. R.; Yuan, B.; Gilman, J. B.; Peischl, J.; Aikin, K. C.; et al. Diurnal Variability and Emission Pattern of Decamethylcyclopentasiloxane (D5) from the Application of Personal Care Products in Two North American Cities. *Environmental Science & Technology* **2018**, *52* (10), 5610-5618. DOI: 10.1021/acs.est.8b00506.

(20) Yucuis, R. A.; Stanier, C. O.; Hornbuckle, K. C. Cyclic siloxanes in air, including identification of high levels in chicago and distinct diurnal variation. *Chemosphere* **2013**, *92* (8), 905-910. DOI: 10.1016/j.chemosphere.2013.02.051.

(21) Anh, H. Q.; Nguyen, H. M. N.; Do, T. Q.; Tran, K. Q.; Minh, T. B.; Tran, T. M. Air pollution caused by phthalates and cyclic siloxanes in Hanoi, Vietnam: Levels, distribution characteristics, and implications for inhalation exposure. *Science of The Total Environment* **2021**, *760*, 143380. DOI: 10.1016/j.scitotenv.2020.143380.

(22) Okan, F.; Odabasi, M.; Yaman, B.; Dumanoglu, Y. Development of a New Passive Sampling Method for the Measurement of Atmospheric Linear and Cyclic Volatile Methyl Siloxanes. *Environmental Science & Technology* **2021**, *55* (8), 4522-4531. DOI: 10.1021/acs.est.1c00227.

(23) Brunet, C. E.; Marek, R. F.; Stanier, C. O.; Hornbuckle, K. C. Concentrations of Volatile Methyl Siloxanes in New York City Reflect Emissions from Personal Care and Industrial Use. *Environmental Science & Technology* **2024**, *58* (20), 8835-8845. DOI: 10.1021/acs.est.3c10752.

(24) Coggon, M. M.; Gkatzelis, G. I.; McDonald, B. C.; Gilman, J. B.; Schwantes, R. H.; Abuhassan, N.; Aikin, K. C.; Arend, M. F.; Berkoff, T. A.; Brown, S. S. Volatile chemical product emissions enhance ozone and modulate urban chemistry. *Proceedings of the National Academy of Sciences* **2021**, *118* (32), e2026653118. DOI: 10.1073/pnas.2026653118.

(25) Verma, R.; Perrier, S.; Michoud, V.; Di Biagio, C.; Gratien, A.; Hawkins, L.; D'Anna, B.; Kammer, J.; Monod, A.; Cantrell, C. *Presence of siloxanes in the ambient air of urban Paris during the ACROSS field campaign*; Copernicus Meetings, 2023.

(26) McLachlan, M. S.; Kierkegaard, A.; Hansen, K. M.; van Egmond, R.; Christensen, J. H.; Skjøth, C. A. Concentrations and Fate of Decamethylcyclopentasiloxane (D5) in the Atmosphere. *Environmental Science & Technology* **2010**, *44* (14), 5365-5370. DOI: 10.1021/es100411w.

(27) Kierkegaard, A.; McLachlan, M. S. Determination of linear and cyclic volatile methylsiloxanes in air at a regional background site in Sweden. *Atmospheric Environment* **2013**, *80*, 322-329. DOI: 10.1016/j.atmosenv.2013.08.001.

(28) Horii, Y.; Ohtsuka, N.; Minomo, K.; Takemine, S.; Motegi, M.; Hara, M. Distribution characteristics of methylsiloxanes in atmospheric environment of Saitama, Japan: Diurnal and seasonal variations and

emission source apportionment. *Science of The Total Environment* **2021**, *754*, 142399. DOI: 10.1016/j.scitotenv.2020.142399.

(29) Krogseth, I. S.; Kierkegaard, A.; McLachlan, M. S.; Breivik, K.; Hansen, K. M.; Schlabach, M. Occurrence and Seasonality of Cyclic Volatile Methyl Siloxanes in Arctic Air. *Environmental Science & Technology* **2013**, *47* (1), 502-509. DOI: 10.1021/es3040208.

(30) Warner, N. A.; Nikiforov, V.; Krogseth, I. S.; Bjørneby, S. M.; Kierkegaard, A.; Bohlin-Nizzetto, P. Reducing sampling artifacts in active air sampling methodology for remote monitoring and atmospheric fate assessment of cyclic volatile methylsiloxanes. *Chemosphere* **2020**, *255*, 126967. DOI: 10.1016/j.chemosphere.2020.126967.

(31) Buser, A. M.; Kierkegaard, A.; Bogdal, C.; Macleod, M.; Scheringer, M.; Hungerbühler, K. Concentrations in ambient air and emissions of cyclic volatile methylsiloxanes in Zurich, Switzerland. *Environmental Science and Technology* **2013**, *47* (13), 7045-7051. DOI: 10.1021/es3046586.
